# Assisted delivery of antisense therapeutics in animal models of heritable neurodegenerative and neuromuscular disorders: a systematic review and meta-analysis

**DOI:** 10.1038/s41598-018-22316-7

**Published:** 2018-03-08

**Authors:** M. Leontien van der Bent, Omar Paulino da Silva Filho, Judith van Luijk, Roland Brock, Derick G. Wansink

**Affiliations:** 1 Department of Cell Biology, Radboud Institute for Molecular Life Sciences (RIMLS), Radboud university medical center, Nijmegen, The Netherlands; 2 Department of Biochemistry, Radboud Institute for Molecular Life Sciences (RIMLS), Radboud university medical center, Nijmegen, The Netherlands; 3 CAPES Foundation, Ministry of Education of Brazil, Brasília, Brazil; 4 Systematic Review Centre for Laboratory Animal Experimentation (SYRCLE), Department of Health Evidence, Radboud university medical center, Nijmegen, The Netherlands

## Abstract

Antisense oligonucleotide (AON)-based therapies hold promise for a range of neurodegenerative and neuromuscular diseases and have shown benefit in animal models and patients. Success in the clinic is nevertheless still limited, due to unfavourable biodistribution and poor cellular uptake of AONs. Extensive research is currently being conducted into the formulation of AONs to improve delivery, but thus far there is no consensus on which of those strategies will be the most effective. This systematic review was designed to answer in an unbiased manner which delivery strategies most strongly enhance the efficacy of AONs in animal models of heritable neurodegenerative and neuromuscular diseases. In total, 95 primary studies met the predefined inclusion criteria. Study characteristics and data on biodistribution and toxicity were extracted and reporting quality and risk of bias were assessed. Twenty studies were eligible for meta-analysis. We found that even though the use of delivery systems provides an advantage over naked AONs, it is not yet possible to select the most promising strategies. Importantly, standardisation of experimental procedures is warranted in order to reach conclusions about the most efficient delivery strategies. Our best practice guidelines for future experiments serve as a step in that direction.

## Introduction

A number of neurodegenerative and neuromuscular disorders are due to known genetic defects. Examples include spinal muscular atrophy (SMA), Duchenne muscular dystrophy (DMD) and familial amyotrophic lateral sclerosis (ALS). Neurodegenerative and neuromuscular disorders are notoriously difficult to treat due to poor accessibility of the affected tissues and because multiple organs need to be reached by a drug. Classical small molecule drugs can alleviate symptoms to a certain degree. One example is Riluzole, which is used for the treatment of ALS^1^. However, as the genetic defects that cause these disorders are known, drugs can be designed to specifically target the mutations. The most complete cure would be obtained by repairing the gene, for example using CRISPR-Cas9 technology, but practical and safety concerns still surround the use of gene therapy, both regarding the use of viral vectors for their delivery^2–4^ and the risk of off-target mutations^5,6^. Thus, a less permanent and less controversial option is to target the mutated messenger RNA (mRNA).

mRNAs can be targeted in a sequence-specific manner by antisense oligonucleotides (AONs). For the purpose of this review, we define AONs as short stretches of single-stranded DNA or RNA or chemically modified versions thereof. This definition also encompasses virally delivered small nuclear RNAs (snRNAs) and AONs encoded by plasmids. siRNAs, on the other hand, are excluded from our analysis, as these are double-stranded and have a markedly different mechanism of action. Depending on their chemistry, AONs can either sterically block (e.g. to modulate splicing) or cause degradation of the target mRNA through RNase H activity. Comprehensive reviews of the use of AONs in neurodegenerative disorders have been published e.g.^7,8^.

Sophisticated chemical modifications of the oligonucleotide backbone or the bases in synthetic AONs also improve endonuclease resistance and affinity for the target mRNA, and serve to reduce toxic effects of AONs. Common modifications include the use of phosphorothioate (PS) linkages in the backbone, often together with sugar modifications such as 2′ hydroxyl methylation or methoxyethylation (2′OMe and MOE, respectively) or constraints such as the 2′-O, 4′-C methylene bridge in locked nucleic acids (LNA). Uncharged variations on the regular structure of oligonucleotides, among which phosphorodiamidate morpholino (PMO) and peptide nucleic acid (PNA) chemistry, are also used^7^.

AONs are and have been tested in clinical trials for several neurodegenerative and neuromuscular diseases, including SMA, familial ALS, DMD, Myotonic Dystrophy type 1 (DM1), Familial Amyloid Polyneuropathy (FAP) and Huntington’s Disease (HD) (clinicaltrials.gov, June 2017). Although the pathological mechanisms of these diseases are rather different, AON therapy is promising for all of them. In the cases of SMA and DMD, antisense therapy is aimed at splice correction, which leads to increased production of functional proteins^7–9^. In ALS, DM1, FAP and HD, on the other hand, antisense treatment is designed to reduce target mRNAs levels or accessibility^7–10^. In ALS, FAP and HD this then results in lower expression of the disease-causing mutant protein. In DM1, AONs block mRNA-protein binding or induce removal of the toxic mRNA molecule that is the cause of the disorder.

Despite the potential of AONs, significant hurdles remain in translating promising preclinical data into effective therapy. One of the greatest limitations for this class of compounds is that they are rapidly cleared from the circulation so that only a fraction of the AONs reaches tissues other than the kidney and liver. Therefore, high doses need to be administered to reach effective concentrations in target tissues such as skeletal muscle and the central nervous system. Tissue specificity can be increased to some extent by using specific administration routes. For example, intrathecal injection increases the delivery efficiency of therapeutic compounds to the central nervous system. This strategy has been employed successfully for SMA using the antisense drug Nusinersen, which was recently approved by the FDA^11–14^ reviewed by^15,16^. Another intrathecally administered antisense drug, which targets SOD1 (IONIS-SOD1_Rx_) for the treatment of ALS^17^, is now in phase 1/2a clinical trial (www.ionispharma.com, December 2017). Unfortunately, local delivery is not a viable option in a multi-organ or systemic disease. Therefore, there is a pressing need for better delivery strategies, as has also been stressed recently by a group of researchers in the field^18^.

Carriers that more efficiently deliver AONs to the target tissues are under wide preclinical investigation^19^. Some of these carriers can be administered systemically, which allows for the targeting of a variety of tissues with a single injection. The carrier strategies that are applied the most can be broadly subdivided into four categories: polymeric, peptide, lipid and viral delivery systems. In the first three categories, AONs are either covalently attached to a carrier or assembled into nanoparticles. In the viral delivery strategies, AONs are encoded by a viral genome.

To our knowledge, clinical studies have thus far been performed mainly with naked AONs. This may at least partly be attributed to the lag time between definition of a study protocol and analysis of the clinical outcome. When the current clinical studies were initiated, the delivery technologies that show promise today were still in their infancy. With limited efficacy being reported in first clinical studies on systemically delivered naked AONs for treatment of DMD (commented on by e.g.^20,21^) and DM1 (myotonic.org, January 2017), the importance of delivery strategies is becoming increasingly clear. However, any decision towards this unexplored terrain carries an enormous risk of failure. Therefore, the question arises which delivery strategies may hold the greatest promise, but also whether sufficient preclinical data has been presented to make such a decision. By investigating a range of disorders with comparable – though not identical – target tissues, we reasoned that we would be able to gain the most comprehensive overview of the state of the field, even if certain promising delivery agents have only been used in one specific disease context so far.

Some excellent reviews have been published on assisted delivery of AONs using cell-penetrating peptides^22–24^, lipid nanoparticles^25^ and adeno-associated virus (AAV) vectors^26^, as well as on the preclinical and clinical use of AONs for neurodegenerative^7,27^ and neuromuscular disorders^28,29^. So far, however, these were classical, narrative reviews. As a consequence, an objective evaluation of the added value of the various delivery strategies has been missing. In contrast, a systematic review, as presented here, formulates a clear-cut research hypothesis. Such a systematic review is an excellent tool to evaluate the studies that have been performed in a manner that is as unbiased as possible through the use of transparent and reproducible methodology, which is defined prior to the execution of the research^30,31^.

In this systematic review, we investigated which delivery strategies are the most promising for AON treatment of heritable neuromuscular and neurodegenerative diseases. To this end, we analysed animal intervention studies that use agents for the delivery of AONs in genetic animal models of these diseases. In total, 95 studies complied with our inclusion criteria. These studies cover a range of AON modifications, as well as various delivery strategies. Study characteristics, biodistribution and toxicity are discussed. In addition, a meta-analysis^31^ was performed to assess the efficacy of the different delivery strategies. Finally, we address reporting quality and present a guideline for preclinical studies on antisense drugs, which will contribute to improved comparability of studies.

## Results

### Study search and selection

A comprehensive literature search, based on the SYRCLE step-by-step guide^32^, was performed with the following main components: 1) intervention (antisense treatment and delivery vector), 2) disease models (heritable neuromuscular and neurodegenerative disorders), and 3) animal population. A detailed description of our literature search strategy, as well as the in- and exclusion criteria can be found in the Methods and Supplementary File S3.

**Figure 1:**
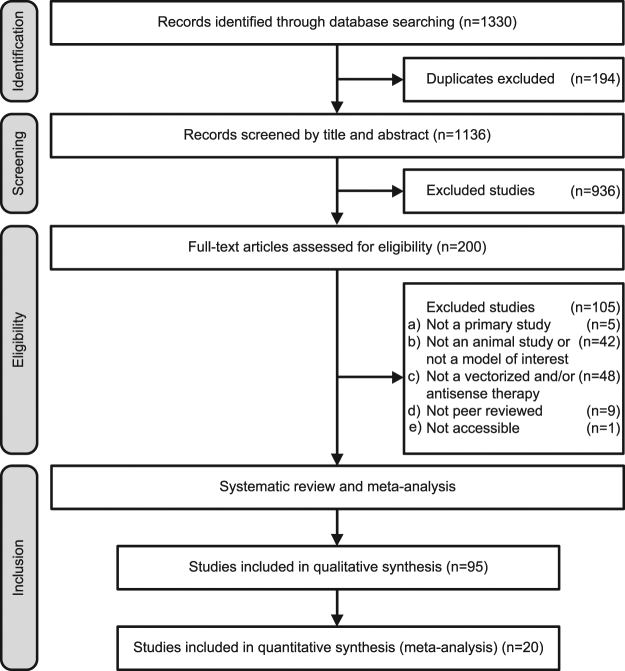
PRISMA flow diagram. Overview of the number of studies that were in- or excluded in each phase of the study selection procedure, as outlined in the PRISMA Statement (Preferred Reporting Items for Systematic Reviews and Meta-Analyses)^65^.

The results of the various stages of the comprehensive search are shown in Fig. 1. The search resulted in 1330 retrieved articles, of which 15% (194/1330) were duplicates or triplicates. Studies were excluded from this systematic review if they were a) not a primary study, b) not an animal study and/or not a genetic animal model of a heritable neuromuscular or neurodegenerative disorder, c) not a vectorized antisense treatment, d) not a peer reviewed article, or e) not accessible. Screening and subsequent eligibility assessment based on these exclusion criteria resulted in inclusion of 95 studies in the systematic review. We were able to include 20 of these studies in our meta-analysis on the basis of the following inclusion criteria: i) direct comparison between vectorized and non-vectorized AONs, and ii) quantification of the mean number or percentage of dystrophin-positive fibres in animal models of DMD, including statistical analysis (SD or SEM). A reference list of the included studies is presented in Supplementary File S1.

### Study characteristics

**Figure 2:**
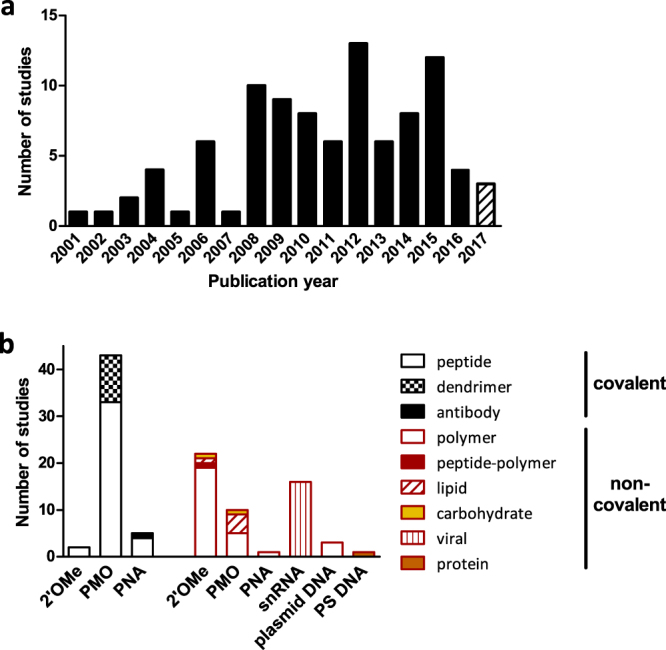
Characteristics of the included studies. (**a**) Number of studies per year. (**b**) Delivery strategies used for each AON chemistry. In case a study investigated multiple AON chemistries or delivery vectors, it was included multiple times in this graph.

Study characteristics were extracted for all of the 95 included studies. The number of studies that investigated delivery strategies for AON delivery increased steadily from 2001 to 2008, after which it has remained at approximately the same level (Fig. 2a). An overview of the characteristics of each study is presented in Supplementary Table S1.

#### Animal models

Out of the 95 included studies, 81 were based on models of DMD (85%), eight on SMA (8%) and two on DM1 (2%). Additionally, single studies for FAP, HD, facioscapulohumeral muscular dystrophy and Fukuyama congenital muscular dystrophy were included.

Mouse models were used in 91 studies and four studies reported the use of dog models for DMD. Two-thirds of the studies (65/95) neglected to report animal sex. In 23 studies (24%), male mice were used, two studies used female mice and five studies used both male and female mice. The fact that most studies appear to use male mice can probably be explained by the fact that DMD is an X-linked recessive disorder and therefore rarely affects females.

#### AON chemistry and delivery characteristics

Seven types of AON chemistries were used in the included articles. Five of these were synthetic AONs. Phosphorodiamidate morpholino oligomers (PMO) were used in the majority of studies (48/95; 51%). The next most common chemistry was 2′-O-methylated RNA (2′OMe; 20%), either with or without a phosphorothioate (PS) backbone (17 and 3 studies, respectively). One of these studies used 2′OMePS with 5-methylcytosine. Four studies used peptide nucleic acid (PNA) type AONs (4%). Two studies used all three of these chemistries (PMO, 2′OMePS and PNA) and two studies compared PMO and 2′OMePS. A single study used DNA with some PS linkages. For the non-synthetic AONs, 14 studies used virally encoded snRNAs (15%) and three studies investigated plasmid-encoded AONs (3%). Two studies used both virally encoded snRNA and synthetic 2′OMePS or PMO.

The type of AON chemistry largely drives the type of delivery systems that can be used. The neutral PMO and PNA chemistries are more easily covalently conjugated to a carrier, whereas the negatively charged 2′OMePS type AONs and plasmid DNA are more amenable to formation of non-covalent polyplexes with cationic polymers or peptides. This is also reflected in the studies that we reviewed.

Out of 95, 47 studies (49%) used carriers that were covalently coupled to the oligonucleotides (Fig. 2b). In this class, 36 studies used peptides as carriers, ten studies used octa-guanidine dendrimers and one study used an antibody conjugate. Peptides were covalently coupled to PMO oligonucleotides in 32 studies, to PNA in three studies, and 2′OMePS in one study. One study compared the efficacy of peptides covalently bound to all three of these AON chemistries. Octa-guanidine dendrimers were always covalently coupled to PMO oligonucleotides. This combination is also called vivo-morpholino. One study used an antibody in combination with a PNA-type AON.

Non-covalent delivery strategies also correspond to 49% (47/95) of the included studies (Fig. 2b). Polymers were explored for 2′OMePS and 2′OMe (17/23), PMO (3/23) and plasmid DNA (3/23) delivery, thus in a total of 23/95 studies (24%). Two studies compared the use of polymers for 2′OMePS and PMO, or 2′OMePS, PMO and PNAs; in another study, polyethylene glycol-polyethylenimine (PEG-PEI) co-polymers with or without peptide conjugate were used for the delivery of 2′OMePS AONs. Five studies used a lipid carrier, of which four for PMO and one for 2′OMe AONs. AONs were delivered using a recombinant AAV in 16 studies, in one of which mice were pre-treated with peptide conjugated PMOs. Single studies used carbohydrates for PMO and 2′OMePS delivery, or a protein for delivery of a DNA oligonucleotide with a partial phosphorothioate backbone.

#### Route of administration

Different routes of administration were used. In total, 41% of the studies (39/95) investigated local injections, against 42% investigating systemic administration (40/95). The remaining 17% investigated both types of administration (16/95).

For local administration, 43 studies (45%) used intramuscular injection and seven studies reported direct injection into the brain, by intracerebroventricular (5%) or intrahippocampal injection (2%). Single studies reported transendocardial injection and liver injection. For systemic administration, 36 studies (38%) used intravenous injection, nine studies used intraperitoneal injection (9%) and two studies used a subcutaneous route of administration (2%). In four studies, multiple systemic routes of administration were compared or combined and in one study oral administration was investigated.

### Biodistribution

**Figure 3:**
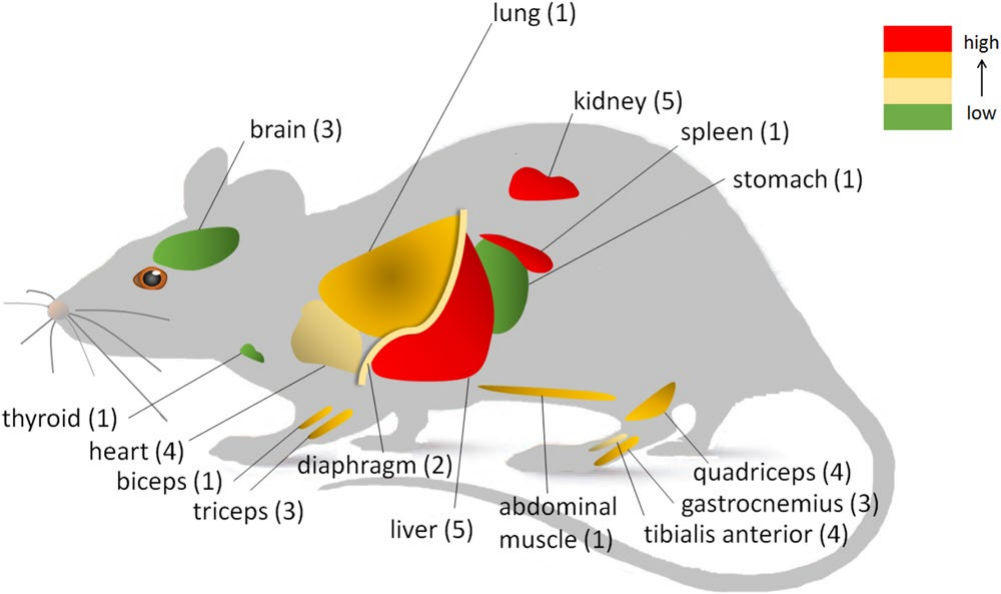
AON biodistribution using various delivery strategies. Schematic overview of *in vivo* biodistribution, based on quantitative data described in^33–37^. Colours indicate AON levels reached in various organs. Numbers between brackets indicate the number of studies that reported on each organ.

For any drug, uptake and clearance by off-target organs such as the liver and kidneys is a major concern. Biodistribution was assessed in ten of the studies that were included in this systematic review, which is equal to 18% of the studies that investigated systemic administration. Five of these studies performed a quantitative analysis. In general, off-target organs accounted for most of the uptake of AONs, even with the use of a delivery strategy (Fig. 3). However our analysis showed that uptake of AONs in target organs was usually more efficient when administered with a delivery vector.

Two of the studies that provided quantitative data examined peptide-conjugated PMOs (PPMOs). Burki *et al*. found higher uptake of PPMOs in all examined tissues. Furthermore, there seemed to be a shift towards less kidney and more liver uptake for the PPMO compared to the naked PMO^33^. Yin *et al*. compared two versions of a PPMO, each conjugated to two peptides. The order in which the two peptides were conjugated appeared to be important, with one variant showing more efficient uptake in muscles, and slightly less uptake in liver than the other variant^34^.

Han *et al*. described that addition of glucose-fructose to the formulation enhanced uptake of both PMO and 2′OMe AONs in muscles. For 2′OMe, the distribution to the liver and kidney did not change compared to saline. For PMO, there seemed to be more uptake by the kidney, although this was not significant^35^. Jirka *et al*. found significantly higher uptake of a peptide-conjugated 2′OMePS AON compared to naked AON in heart, an organ that is generally difficult to reach. In all other tissues that they examined, the uptake of the peptide-conjugated AON was also increased compared to naked AON^36^.

Finally, Lee *et al*. investigated an antibody-conjugated PNA for which they observed the highest concentrations in spleen, liver, kidney and lung (in that order) of a mouse model of HD. They found that the antibody enhanced uptake in the brain of healthy mice compared to unconjugated PNA. Interestingly, uptake of the antibody-PNA conjugate was higher in transgenic mice than in healthy littermates. The authors argued that this is due to sequence-specific sequestration by the target mRNA^37^.

### Toxicity

**Figure 4:**
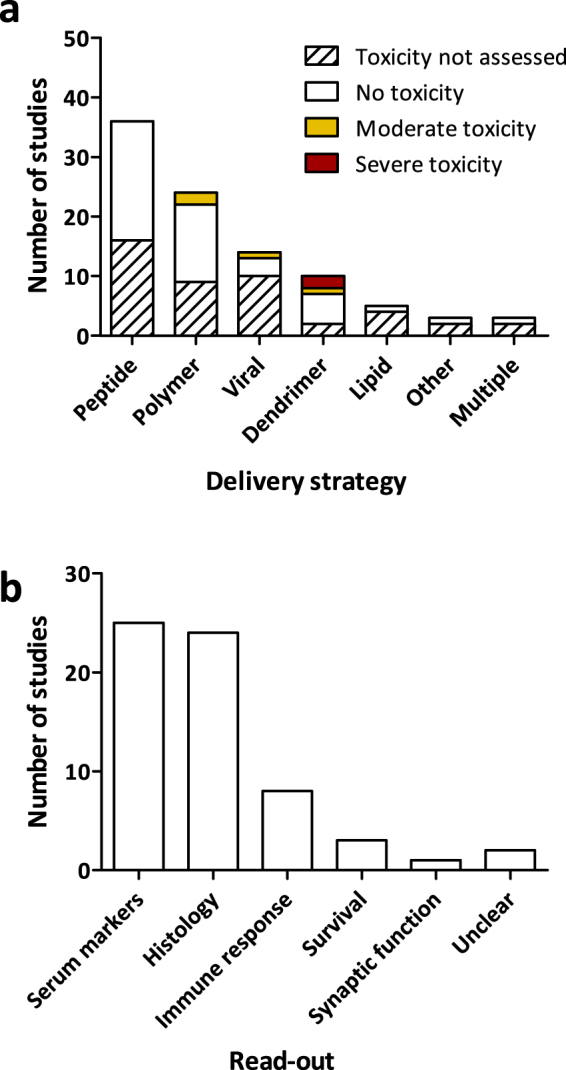
Toxicity assessment. (**a**) Reported toxicity for the various delivery strategies as specified by number of studies. (**b**) Use of different read-outs to assess toxicity. Some studies used multiple read-outs.

Fifty percent of the papers discussed toxicity of the antisense treatment (48/95). In the majority of cases, no overt signs of toxicity were observed (Fig. 4a). Parameters that were assessed include histological examination for tissue damage in for example the liver, levels of liver and kidney inflammatory markers – such as alkaline phosphatase (ALP) and blood urea nitrogen (BUN) – in the serum, and production of specific antibodies against the therapeutic compound (Fig. 4b).

For four delivery strategies, toxicity was reported. For AAVs, an immune response was reported in one article^38^. This immune response was elicited upon re-administration of the vector. Other than that, no toxic effects were noted for AAVs. Octa-guanidine dendrimer-coupled PMOs (vivo-morpholinos) were reported to cause severe toxicity when administered via intracerebroventricular injection in newborn mice^39,40^. Twice weekly intravenous administration of 20 mg/kg vivo-morpholinos to four-week-old mice also led to toxicity. Reducing the dose to 15 mg/kg for the first two weeks, however, eliminated this toxic effect^41^. The remaining studies that used vivo-morpholinos administered lower doses and did not observe overt toxicity. One study using a peptide-conjugated PMO performed a dose escalation and reported a LD_50_ of 85 mg/kg. No toxicity was observed for the lower doses of 6 and 30 mg/kg^42^. Finally, PEI was described to have local toxic effects upon intramuscular injection, specifically high molecular weight PEI^43,44^. For copolymers with PEI or Tween-85 grafted PEI, no obvious toxicity was observed.

### Reporting quality and risk of bias assessment

**Figure 5:**
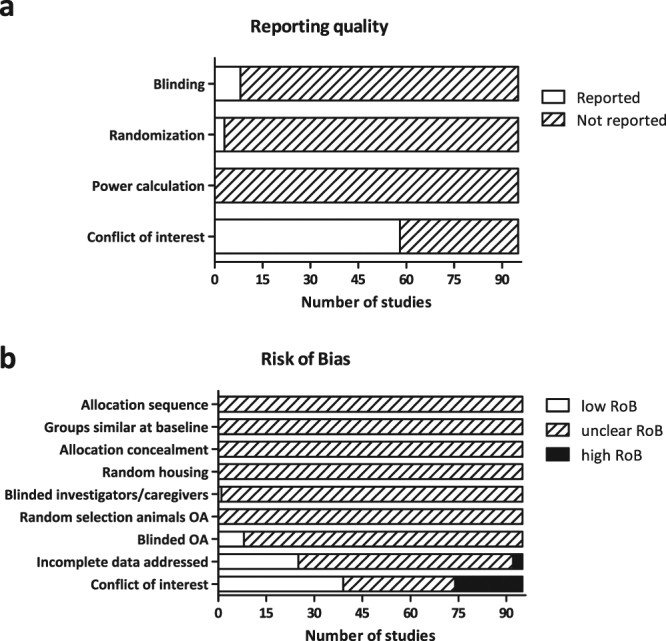
Reporting quality and risk of bias assessment. (**a**) The reporting quality and (**b**) risk of bias were assessed for all included studies. Allocation refers to the assignment of an animal to a treatment group. Random selection of animals refers to the order in which animals are picked for the outcome assessment (OA).

The study quality and risk of bias assessment, based on the SYRCLE risk of bias tool for animal studies^45^, is shown in Fig. 5. As additional item, conflict of interest (both reporting and risk of bias) was included. An overview of the risk of bias and reporting quality assessment for each individual study is presented in Supplementary Table S2.

Of the 95 included studies, three studies reported that their experiments were randomised at any level and eight studies mentioned that parts of their experiments were blinded. Even then, blinding was only addressed at the level of the outcome assessment. None of the studies showed a power calculation or otherwise explained the size of their experimental groups. Competing financial interests were reported more frequently, with 58 out of 95 studies (61%) stating whether there was a conflict of interest. In 21 studies (22%), the risk of bias was classified as high due to a potential conflict of interest (Fig. 5b). This was the case if a potential conflict of interest was reported by the authors, or if there was no statement about conflicts of interest, whereas at least one of the authors was affiliated with a company that develops AON-based therapy. The risk of bias on most of the remaining items was unclear, which can be attributed for a large part to the low reporting quality.

### Meta-analysis

Prior to performing the comprehensive search, it was not clear which disorders would be represented sufficiently to be used in a meta-analysis. Therefore, in the systematic review protocol, a number of possible outcome measures were mentioned. In the end, only DMD was well represented in our data set and only one outcome measure was frequently described and quantified: the number or percentage of dystrophin-positive fibres. Therefore, only this outcome measure qualified for meta-analysis.

**Figure 6:**
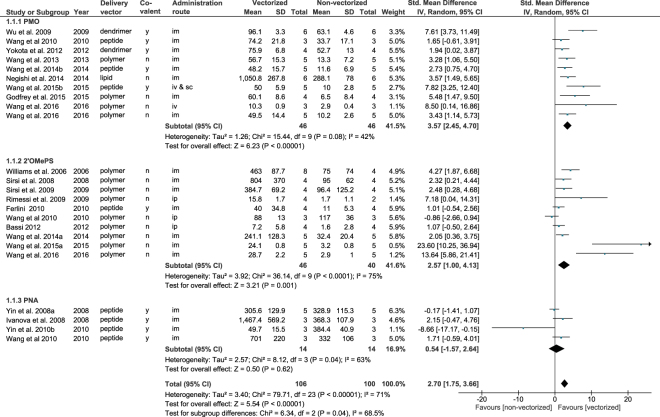
Forest plot of the effect of vectorization of AONs on dystrophin restoration in DMD models. Results of the meta-analysis of studies that compared dystrophin restoration after treatment with vectorized and non-vectorized AONs in genetic animal models of DMD. All values were obtained from tibialis anterior or, if data for this muscle was not reported, quadriceps. If multiple vectors were assessed in the same experiment, the vector with the highest mean was used. An overall beneficial effect of vectorization was observed. For PNA-type AONs, this effect was not significant. Data are presented as Standardized Mean Difference and 95% confidence interval (CI). im = intramuscular, iv = intravenous, sc = subcutaneous, ip = intraperitoneal.

Twenty studies quantified the amount of dystrophin-positive fibres and compared vectorized with naked AONs. For these studies, the reported values were extracted and subsequently used to calculate the effect size expressed as the standardised mean difference (SMD; Fig. 6). By pooling these effect sizes in a random-effects model, we found that vectorization increases the effect of AONs on dystrophin restoration in animal models of DMD (SMD: 2.70 with a 95% confidence interval (CI) of [1.75, 3.66]). The effects per subgroup show that significant effects are found for PMO (SMD: 3.57, CI: [2.45, 4.70]) and 2′OMe chemistry AONs (SMD: 2.57, CI: [1.00, 4.13]). Only for PNA-type AONs, no statistically significant effect of vectorization was observed (SMD: 0.54, 95% CI: [−1.57, 2.64]).

## Discussion

Over the past years, the development of antisense-based therapeutics has seen an increase in research efforts in a variety of neuromuscular and neurodegenerative disorders, such as Duchenne muscular dystrophy^46^, myotonic dystrophy type 1^10^, spinal muscular atrophy^47^, spinocerebellar ataxia type 2^48^ and type 3 (also known as Machado-Joseph disease)^49,50^, Huntington’s disease^51,52^, spinal and bulbar muscular atrophy^53,54^ and ALS and frontotemporal dementia^55,56^. Excitingly, multiple antisense drugs are in advanced clinical trials and antisense therapies for Duchenne muscular dystrophy and spinal muscular atrophy have been approved by the FDA. Unfortunately, not all clinical trials have had equally promising results, especially when systemic delivery is needed, for example in Duchenne muscular dystrophy^20,21^ and myotonic dystrophy type 1 (myotonic.org, January 2017). Most of this lack of efficacy can be attributed to low delivery efficiency of AONs after systemic delivery^18^. It is therefore more than timely to critically and objectively evaluate the potential benefit of delivery strategies.

In this systematic review, we identified 95 studies that used vectorized antisense therapeutics in animal models of neuromuscular and neurodegenerative disorders. Although our search aimed to identify studies in a range of disease models, we unexpectedly found that the vast majority (81 out of 95) of these studies were performed on mouse models of Duchenne Muscular Dystrophy. Nevertheless, we decided to maintain the broad scope of the review, in order to have the opportunity to also include findings from other neuromuscular and neurodegenerative disorders that would be relevant for other fields. Twenty studies that quantified the amount of dystrophin-positive fibres in DMD models after treatment with naked and vectorized AONs conformed with our inclusion criteria for a meta-analysis. Thus, we found that assisted delivery increases efficacy of antisense therapy, especially for 2′OMePS and PMO type oligonucleotides.

The strength and novelty of our systematic review derives from the fact that it is based on a well-defined hypothesis and search strategy and therefore provides an assessment of the results presented by the current literature that is as unbiased as possible. Using an elaborate search strategy, we strove to include literature on all available delivery strategies and AON chemistries in a range of disease models. To our knowledge, this is the first quantitative comparison of different delivery vectors for AONs. Unfortunately, low comparability between studies limited the possibilities for meta-analysis, as is further discussed below.

One question that begs to be answered is why, thus far, the focus of research has been mostly on DMD. One factor that likely contributes is the high efficiency of AON treatment in DMD models, which has been hypothesized by Hoffman and colleagues to be up to 100-fold higher than in other disease models due to two factors^57^. Firstly, they argue that the more permeable plasma membranes in DMD muscles may lead to up to 10-fold more efficient delivery of AONs. Secondly, the authors reason that it is easier to achieve effective upregulation of a target protein, as effected by exon skipping, than it is to have complete knockdown. These characteristics could explain the promising pre-clinical and clinical results that have been obtained for DMD, especially compared to previous endeavours such as Alicaforsen, which downregulates the immune adhesion molecule ICAM-1 in inflammatory diseases^58^. Another possible factor is that multiple well-characterized animal models of DMD exist^59^. Likely due to the same reasons, DMD research is also on the front line when it comes to the assisted delivery of antisense therapy. However, the number of studies on SMA (8/95) in which AONs also cause splice correction is also increasing. Hopefully, the insights provided by the DMD research will allow the rest of the neuromuscular and neurodegenerative field to follow.

Our meta-analysis of the DMD studies that compared naked with vectorized AONs and quantified dystrophin restoration clearly shows that delivery vectors enhance the efficacy of AON treatment. This holds true for systemic as well as local administration. Although only a single outcome measure could be used in this meta-analysis, this immunohistochemical assessment of the amount of dystrophin-positive fibres is probably the most indicative of functional dystrophin, as compared with other methods such as Western blotting, which rely solely on intensity measurements^60^. Interestingly, AON chemistry seems to dictate the extent to which a delivery vector provides added value. Specifically, PNA does not seem to benefit from peptide carriers. This might be explained partly by the fact that PNA-type AONs had a quite large effect when administered without delivery vehicle. Charge of the AON backbone is apparently not the driving force for the high efficacy of naked PNAs, as this effect is not observed for the PMO chemistry. The route of administration did not appear to influence the effect size for any of the chemistries. Unfortunately, the high degree of correlation between the type of oligonucleotide and the type of delivery vector used preclude conclusions about the type of delivery vector that is the most potent.

Based on the available data on biodistribution and toxicity, it is not possible to point out the most promising candidate delivery strategies either. Biodistribution was only reported in a handful of studies, and no single delivery vector appears to effectively decrease accumulation in off-target organs. In some cases, the liver/kidney ratio was changed by the use of a delivery vector, but none of these papers report a selective increase in uptake in the target organs. Although a number of studies reported on toxicity, it is likely that toxic doses were often not included in the study design. Additionally, most studies only included a limited set of toxicity markers. Therefore, it is not possible to gauge the therapeutic windows of the various vectorization strategies. The scarcity of biodistribution and toxicity data might in part be explained by the fact that we have chosen only to include experiments that were performed in genetic animal models of heritable neurodegenerative and neuromuscular disorders. We reasoned that this would be the closest to the clinical situation and that only in such models a clinically relevant effect could be measured. Therefore, we did not include studies and experiments that were performed on wild type mice, which might have presented more biodistribution data.

Based on the number of studies that have been performed with certain combinations of delivery vectors and AONs, it would appear that PMO conjugates with peptides or dendrimers are the most promising. This combination is closely followed by polymeric nanoparticles with 2′OMePS AONs and AAV-delivered snRNAs. However, the number of studies performed with any particular AON chemistry or class of delivery vectors is probably also influenced by other factors, such as sponsorship by holders of intellectual property. The fact that few studies directly compared different delivery strategies or aimed at reproducing studies that were done with a certain delivery vector makes it very difficult to draw conclusions about the most promising delivery vector at this point in time.

Favourable properties of covalent conjugates are their relatively easy production, their stability and the fact that they can be well characterised. Covalent conjugates are considered as a single active pharmaceutical ingredient (API), which means that only a single compound needs to be thoroughly tested for toxicity. On the other hand, if non-covalent carriers were used, these would be seen as excipients, meaning that they are not part of the API. Provided that they have been approved beforehand, this would eliminate the need for toxicology tests for the carrier. Except for the AAV vectors, certain polymers and the TAT peptide, however, mostly experimental carrier systems have been used.

Although the number of studies in the meta-analysis was quite substantial, the variety of different delivery vehicles, AON chemistries and other experimental factors such as route of administration and dosing regimen precluded any definitive conclusions about the most promising delivery vehicles. Furthermore, several factors limited the number of studies that could be included in the meta-analysis. One of the inclusion criteria for the meta-analysis was the use of naked AONs as a control group. We reasoned that only if this control was present, it would be possible to determine the added value of a delivery vector. However, this control group was only included in about 40 percent of the studies (40/95). The use of different (primary) outcome measures between studies on the same disease made it difficult to compare results. In addition, results such as the number of dystrophin-positive fibres or the degree of splice correction were often not quantified, and therefore not usable for meta-analysis. Finally, the use of a very disease-specific outcome measure, in this case dystrophin-positive fibres, made it very difficult to compare results between models of different diseases. If a more general, upstream outcome measure, such as splice correction, had been used and quantified more frequently, this would have increased the number of possible comparisons for the meta-analysis.

During our review, it became clear that the reporting quality of the majority of the studies was low. With an ongoing discussion on the predictive value of preclinical animal studies, the issue of reporting quality and reproducibility of preclinical animal studies has received considerable attention (e.g.^61,62^). Still, despite the fact that there are various guidelines for more rigorous reporting of methods and results (reviewed by^63^), they nevertheless still receive little attention. We feel that this is a critical factor that should be addressed in order to be able to better compare different studies, which will ultimately benefit the field as a whole.

Therefore, we would like to stress again the importance of adhering to such guidelines, and want to highlight the following points for better comparability of preclinical animal studies on antisense drugs specifically:

- Study design and reporting should conform with existing guidelines, such as the NINDS rigor guidelines and ARRIVE guidelines^61,64^. This includes randomisation, blinding, sample size calculations based on expected effect size and appropriate handling of the data
- Use of appropriate control groups, including a control group that receives naked AONs in cases when the effect of a delivery vector is investigated
- Use of a standard, broad set of outcome measures and quantification thereof. The following outcomes should be taken into account:
  - Biodistribution (including target tissues and common off-target tissues such as liver, kidney and spleen)
  - Direct effect of the AON (e.g. splice switch or knock-down of the target mRNA)
  - Downstream effect of the AON on the molecular level (e.g. protein expression, splicing of downstream targets)
  - Downstream effect of the AON on the physiological level (e.g. motor function, behavioural effects, survival).

In conclusion, our systematic review demonstrates that the application of AONs in heritable neuromuscular and neurodegenerative disorders is not ready yet to make a decision for the most promising delivery strategies. What has become clear is that PMOs conjugated to peptides or octaguanidine dendrimers have been studied most intensively, followed by polymeric nanoparticles with the charged 2′OMePS-type of AON and adeno-associated viruses encoding snRNAs. Hopefully, future studies in animal models of a wider range of neuromuscular and neurodegenerative disorders will shed more light on this issue. It is then crucial that these studies implement a rigorous study design and more transparent reporting and also compare delivery strategies.

## Methods

### Literature search strategy, inclusion and exclusion criteria

The present systematic review was structured to answer which delivery strategies are most effective to enhance the efficacy of AONs in animal models of heritable neurodegenerative and neuromuscular diseases. The structure of this study was pre-specified in a review protocol^30^ which is published on the SYRCLE website (https://issuu.com/radboudumc/docs/the_most_suitable_form_to_delivery_?e=28355229/48257130). The PRISMA checklist^65^ was used as a guideline for reporting (Supplementary File S2).

An extensive comprehensive search strategy was designed for Pubmed, Embase and Web of Science, based on the SYRCLE step-by-step guide^32^. The search strategy consisted of three main components: 1) intervention, which comprised antisense treatment and delivery vector, 2) disease models, which included all heritable neuromuscular and neurodegenerative disorders, and 3) animal population, using SYRCLE’s search filters for animal studies for Pubmed and Embase^66,67^. The full electronic search strategies are available in Supplementary File S3. The search was performed last on May 18, 2017 in PubMed (all years), Embase via Ovid (1974 to present) and the Web of Science Core Collection (1945 to present). No restrictions regarding language or publication date were applied.

All retrieved records from the three databases were combined in an EndNote X7 file (Thomson Reuters (Scientific) LLC, Philadelphia, USA) for automatic and manual removal of duplicates. The unique records were exported to EROS (Early Review Organising Software; Institute of Clinical Effectiveness and Health Policy, Buenos Aires, Argentina), to be screened by title and abstract by two independent reviewers (MLB and OPSF), based on predefined inclusion and exclusion criteria. Studies included by at least one reviewer during the title/abstract screening were screened full text for eligibility. Exclusion criteria were: a) not a primary study, b) not an animal study and/or not a genetic animal model of a heritable neuromuscular or neurodegenerative disorder, c) not a vectorized antisense treatment, d) not a peer reviewed article, and e) not accessible. Differences in eligibility classification between the reviewers were discussed until consensus was reached. Additional inclusion criteria for the meta-analysis were i) direct comparison between vectorized and non-vectorized AONs, and ii) quantification of the mean number or percentage of dystrophin positive fibres in animal models of DMD, including standard deviation (SD) or standard error of the mean (SEM).

### Study Characteristics and Risk of Bias analysis

Of all the eligible studies, the study characteristics were extracted by one of the two reviewers and crosschecked by the other reviewer (MLB or OPSF). The extracted data was compiled into the study characteristics table (Supplementary Table S1). The reliability of the included articles at the study level was assessed using an adaptation of SYRCLE’s risk of bias tool for animal studies^45^. Additional items were added to detect possible conflict of interests in the included studies. A complete overview of items and scores per study is given in Supplementary Table S2. This data was used only for the qualitative data synthesis.

### Meta-analysis

A meta-analysis was performed when at least two studies with the outcome measure of dystrophin-positive fibres in animal models for DMD were retrieved. Other outcome measures that were originally formulated in the protocol were not suited for meta-analysis. For each study, the mean and variance were extracted by one reviewer and were crosschecked by the other reviewer (MLB or OPSF). In case these quantitative data were not explicitly mentioned in the article, digital ruler software (A Ruler For Windows; http://www.arulerforwindows.com/) was used to extract the data from the presented graphs. If the SEM was reported, the SD was calculated using the reported number of animals per group.

The statistical analysis was performed using Review Manager 5.3 (Cochrane Reviews, London, UK). Subgroups were predefined and based on AON chemistry. Due to the high degree of correlation between AON chemistry and delivery vector, no separate subgroup analysis could be performed for delivery strategies. The remaining subgroup analyses that were originally formulated in the protocol could not be performed because of the low number of studies per subgroup and high correlation between factors. To avoid including the same control animals more than once, only the outcome of the vector that gave the highest mean effect was included if multiple delivery vectors were compared within an experiment. In most cases, these comparisons were between different variants of a certain delivery vector.

From the mean and SD values extracted from the studies, the standardised mean difference (SMD) and 95% confidence interval (CI) were calculated. We used the SMD as a measure for the effect size to control for the fact that different measurement scales were used to quantify the amount of dystrophin-positive fibres (number versus percentage). The SMD is the difference in effect between naked and vectorized AONs, divided by the pooled SD. A random effects inverse variance model was then used to calculate the summary effect estimates, because of the large variation between studies (e.g. administration route and dosing regimens)^31^. Heterogeneity was assessed using I^2^.

### Data availability

All data generated or analysed during this study are included in this published article and its Supplementary Information files.

## Electronic supplementary material

Supplementary Files S1-3

Supplementary Table S1

Supplementary Table S2

## References

[1] Miller RG, Mitchell JD, H MD, Moore DH (2012). Riluzole for amyotrophic lateral sclerosis (ALS)/ motor neuron disease (MND). Cochrane Database Syst Rev.

[2] Cesana, D., Volpin, M., Serina Secanechia, Y. N. & Montini, E. In *Safety and Efficacy of Gene-Based Therapeutics for Inherited Disorders* (ed. Brunetti-Pierri, N.) 9–35 (Springer International Publishing, 2017).

[3] Harmon, A. W. & Byrnes, A. P. In *Safety and Efficacy**of Gene-Based Therapeutics for Inherited Disorders* (ed. Brunetti-Pierri, N.) 37–60 (Springer International Publishing, 2017).

[4] Kuranda, K. & Mingozzi, F. In *Safety and Efficacy of Gene-Based Therapeutics for Inherited Disorders* (ed. Brunetti-Pierri, N.) 77–112 (Springer International Publishing, 2017).

[5] Pattanayak V (2013). High-throughput profiling of off-target DNA cleavage reveals RNA-programmed Cas9 nuclease specificity. Nat Biotechnol.

[6] Fu Y (2013). High-frequency off-target mutagenesis induced by CRISPR-Cas nucleases in human cells. Nat Biotechnol.

[7] Evers MM, Toonen LJa, van Roon-Mom WMC (2015). Antisense oligonucleotides in therapy for neurodegenerative disorders. Adv Drug Deliv Rev.

[8] Khorkova O, Wahlestedt C (2017). Oligonucleotide therapies for disorders of the nervous system. Nat Biotechnol.

[9] Kole, R., Krainer, A. R. & Altman, S. RNA therapeutics: beyond RNA interference and antisense oligonucleotides. *Nat Rev Drug Discov* **11**, (2012). 10.1038/nrd3625 PMC4743652 22262036

[10] Gao Z, Cooper TA (2013). Antisense oligonucleotides: rising stars in eliminating RNA toxicity in myotonic dystrophy. Hum Gene Ther.

[11] Chiriboga CA (2016). Results from a phase 1 study of nusinersen (ISIS-SMNRx) in children with spinal muscular atrophy. Neurology.

[12] Finkel RS (2016). Treatment of infantile-onset spinal muscular atrophy with nusinersen: a phase 2, open-label, dose-escalation study. Lancet.

[13] Haché M (2016). Intrathecal Injections in Children With Spinal Muscular Atrophy. J Child Neurol.

[14] Finkel RS (2017). Nusinersen versus Sham Control in Infantile-Onset Spinal Muscular Atrophy. N Engl J Med.

[15] Aartsma-Rus A (2017). FDA Approval of Nusinersen for Spinal Muscular Atrophy Makes 2016 the Year of Splice Modulating Oligonucleotides. Nucleic Acid Ther.

[16] Corey DR (2017). Nusinersen, an antisense oligonucleotide drug for spinal muscular atrophy. Nat Neurosci.

[17] Miller TM (2013). An antisense oligonucleotide against SOD1 delivered intrathecally for patients with SOD1 familial amyotrophic lateral sclerosis: A phase 1, randomised, first-in-man study. Lancet Neurol.

[18] Godfrey C (2017). Delivery is key: lessons learnt from developing splice-switching antisense therapies. EMBO Mol Med.

[19] Juliano RL (2016). The delivery of therapeutic oligonucleotides. Nucleic Acids Res.

[20] Lu Q, Cirak S, Partridge T (2014). What Can We Learn From Clinical Trials of Exon Skipping for DMD?. Mol Ther — Nucleic Acids.

[21] Kesselheim AS, Avorn J (2016). Approving a Problematic Muscular Dystrophy Drug. JAMA.

[22] Lebleu B (2008). Cell penetrating peptide conjugates of steric block oligonucleotides. Adv Drug Deliv Rev.

[23] Lehto T, Kurrikoff K, Langel Ü (2012). Cell-penetrating peptides for the delivery of nucleic acids. Expert Opin Drug Deliv.

[24] Boisguerin P (2015). Delivery of therapeutic oligonucleotides with cell penetrating peptides. Adv Drug Deliv Rev.

[25] Wang Y, Miao L, Satterlee A, Huang L (2015). Delivery of oligonucleotides with lipid nanoparticles. Adv Drug Deliv Rev.

[26] Danos O (2008). AAV vectors for RNA-based modulation of gene expression. Gene Ther.

[27] Zalachoras I, Evers MM, van Roon-Mom WMC, Aartsma-Rus AM, Meijer OC (2011). Antisense-Mediated RNA Targeting: Versatile and Expedient Genetic Manipulation in the Brain. Front Mol Neurosci.

[28] Douglas AGL, Wood MJA (2013). Splicing therapy for neuromuscular disease. Mol Cell Neurosci.

[29] Jirka S, Aartsma-Rus A (2015). An update on RNA-targeting therapies for neuromuscular disorders. Curr Opin Neurol.

[30] De Vries RBM (2015). A protocol format for the preparation, registration and publication of systematic reviews of animal intervention studies. Evidence-based Preclin Med.

[31] Vesterinen HM (2014). Meta-analysis of data from animal studies: A practical guide. J Neurosci Methods.

[32] Leenaars M (2012). A step-by-step guide to systematically identify all relevant animal studies. Lab Anim.

[33] Burki U (2015). Development and Application of an Ultrasensitive Hybridization-Based ELISA Method for the Determination of Peptide-Conjugated Phosphorodiamidate Morpholino Oligonucleotides. Nucleic Acid Ther.

[34] Yin H (2013). Context Dependent Effects of Chimeric Peptide Morpholino Conjugates Contribute to Dystrophin Exon-skipping Efficiency. Mol Ther — Nucleic Acids.

[35] Han G (2016). Hexose enhances oligonucleotide delivery and exon skipping in dystrophin-deficient mdx mice. Nat Commun.

[36] Jirka SMG (2014). Peptide Conjugation of 2′- O -methyl Phosphorothioate Antisense Oligonucleotides Enhances Cardiac Uptake and Exon Skipping in mdx Mice. Nucleic Acid Ther.

[37] Lee HJ, Boado RJ, Braasch Da, Corey DR, Pardridge WM (2002). Imaging Gene Expression in the Brain *In Vivo* in a Transgenic Mouse Model of Huntington’ s Disease with an Antisense Radiopharmaceutical and Drug-Targeting Technology. J Nucl Med.

[38] Vulin A (2012). Muscle Function Recovery in Golden Retriever Muscular Dystrophy After AAV1-U7 Exon Skipping. Mol Ther.

[39] Zhou H (2013). A Novel Morpholino Oligomer Targeting ISS-N1 Improves Rescue of Severe Spinal Muscular Atrophy Transgenic Mice. Hum Gene Ther.

[40] Nizzardo M (2014). Effect of Combined Systemic and Local Morpholino Treatment on the Spinal Muscular Atrophy Δ7 Mouse Model Phenotype. Clin Ther.

[41] Widrick JJ, Jiang S, Choi SJ, Knuth ST, Morcos PA (2011). An octaguanidine-morpholino oligo conjugate improves muscle function of mdx mice. Muscle Nerve.

[42] Wu B (2012). Long-Term Rescue of Dystrophin Expression and Improvement in Muscle Pathology and Function in Dystrophic mdx Mice by Peptide-Conjugated Morpholino. Am J Pathol.

[43] Bremmer-Bout M (2004). Targeted Exon Skipping in Transgenic hDMD Mice: A Model for Direct Preclinical Screening of Human-Specific Antisense Oligonucleotides. Mol Ther.

[44] Wang M (2014). Evaluation of Tris{[}2-(Acryloyloxy) Ethyl] Isocyanurate Cross-Linked Polyethylenimine as Antisense Morpholino Oligomer Delivery Vehicle in Cell Culture and Dystrophic mdx Mice. Hum Gene Ther.

[45] Hooijmans CR (2014). SYRCLE’s risk of bias tool for animal studies. BMC Med Res Methodol.

[46] Aartsma-Rus A (2017). Development of Exon Skipping Therapies for Duchenne Muscular Dystrophy: A Critical Review and a Perspective on the Outstanding Issues. Nucleic Acid Ther.

[47] Porensky PN, Burghes AH (2013). Antisense oligonucleotides for the treatment of spinal muscular atrophy. Hum Gene Ther.

[48] Scoles DR (2017). Antisense oligonucleotide therapy for spinocerebellar ataxia type 2. Nature.

[49] Moore LR (2017). Evaluation of Antisense Oligonucleotides Targeting ATXN3 in SCA3 Mouse Models. Mol Ther - Nucleic Acids.

[50] Toonen LJA, Rigo F, van Attikum H, van Roon-Mom WMC (2017). Antisense Oligonucleotide-Mediated Removal of the Polyglutamine Repeat in Spinocerebellar Ataxia Type 3 Mice. Mol Ther - Nucleic Acids.

[51] Kordasiewicz HB (2012). Sustained Therapeutic Reversal of Huntington’s Disease by Transient Repression of Huntingtin Synthesis. Neuron.

[52] Southwell AL (2014). *In vivo* evaluation of candidate allele-specific mutant huntingtin gene silencing antisense oligonucleotides. Mol Ther.

[53] Lieberman AP (2014). Peripheral Androgen Receptor Gene Suppression Rescues Disease in Mouse Models of Spinal and Bulbar Muscular Atrophy. Cell Rep.

[54] Sahashi K (2015). Silencing neuronal mutant androgen receptor in a mouse model of spinal and bulbar muscular atrophy. Hum Mol Genet.

[55] Lagier-Tourenne C (2013). Targeted degradation of sense and antisense C9orf72 RNA foci as therapy for ALS and frontotemporal degeneration. Proc Natl Acad Sci.

[56] Becker LA (2017). Therapeutic reduction of ataxin-2 extends lifespan and reduces pathology in TDP-43 mice. Nature.

[57] Hoffman EP (2011). Restoring Dystrophin Expression in Duchenne Muscular Dystrophy Muscle. Am J Pathol.

[58] Yacyshyn B (2007). A Randomized, Double-Masked, Placebo-Controlled Study of Alicaforsen, an Antisense Inhibitor of Intercellular Adhesion Molecule 1, for the Treatment of Subjects With Active Crohn’s Disease. Clin Gastroenterol Hepatol.

[59] Willmann R, Possekel S, Dubach-Powell J, Meier T, Ruegg MA (2009). Mammalian animal models for Duchenne muscular dystrophy. Neuromuscul Disord.

[60] Anthony K (2014). Dystrophin quantification: Biological and translational research implications. Neurology.

[61] Landis SC (2012). A call for transparent reporting to optimize the predictive value of preclinical research. Nature.

[62] Begley CG, Ellis LM (2012). Drug development: Raise standards for preclinical cancer research. Nature.

[63] Henderson, V. C., Kimmelman, J., Fergusson, D., Grimshaw, J. M. & Hackam, D. G. Threats to Validity in the Design and Conduct of Preclinical Efficacy Studies: A Systematic Review of Guidelines for *In Vivo* Animal Experiments. *PLoS Med* **10** (2013). 10.1371/journal.pmed.1001489 PMC3720257 23935460

[64] Kilkenny C, Browne WJ, Cuthill IC, Emerson M, Altman DG (2010). Improving Bioscience Research Reporting: The ARRIVE Guidelines for Reporting Animal Research. PLoS Biol.

[65] Moher, D. *et al*. Preferred reporting items for systematic reviews and meta-analyses: The PRISMA statement. *PLoS Med* **6** (2009). 10.1371/journal.pmed.1000097 PMC2707599 19621072

[66] Hooijmans CR, Tillema A, Leenaars M, Ritskes-Hoitinga M (2010). Enhancing search efficiency by means of a search filter for finding all studies on animal experimentation in PubMed. Lab Anim.

[67] de Vries RBM, Hooijmans CR, Tillema A, Leenaars M, Ritskes-Hoitinga M (2014). Updated version of the Embase search filter for animal studies. Lab Anim.

