# Supplementary material for: Assisted delivery of antisense therapeutics in animal models of heritable neurodegenerative and neuromuscular disorders: a systematic review and meta-analysis
Source: Sci Rep. 2018 Mar 8;8:4181. doi: 10.1038/s41598-018-22316-7 (PMC5843643; doi:10.1038/s41598-018-22316-7)
Supplement: Supplementary file 1 — Supplementary Files S1-3 [file 41598_2018_22316_MOESM1_ESM.pdf]

Assisted delivery of antisense therapeutics in animal models of heritable neurodegenerative and neuromuscular disorders: a systematic review and meta-analysis

M. Leontien van der Bent[1,2¶], Omar Paulino da Silva Filho[2,3¶], Judith van Luijk[4], Roland Brock[2&], Derick G. Wansink[1&*]

File S1. Reference list of the included studies.

1. Alter J, Sennoga CA, Lopes DM, Eckersley RJ, Wells DJ. Microbubble Stability is a Major Determinant of the Efficiency of Ultrasound and Microbubble Mediated in vivo Gene Transfer. Ultrasound Med Biol. 2009;35: 976–984. doi:10.1016/j.ultrasmedbio.2008.12.015
2. Aoki Y, Yokota T, Nagata T, Nakamura A, Tanihata J, Saito T, et al. Bodywide skipping of exons 45-55 in dystrophic mdx52 mice by systemic antisense delivery. Proc Natl Acad Sci. 2012;109: 13763–13768. doi:10.1073/pnas.1204638109
3. Bartoli M, Poupiot J, Goyenvalle A, Perez N, Garcia L, Danos O, et al. Noninvasive monitoring of therapeutic gene transfer in animal models of muscular dystrophies. Gene Ther. 2006;13: 20–28. doi:10.1038/sj.gt.3302594
4. Bassi E, Falzarano S, Fabris M, Gualandi F, Merlini L, Vattemi G, et al. Persistent Dystrophin Protein Restoration 90 Days after a Course of Intraperitoneally Administered Naked 2'OMePS AON and ZM2 NP-AON Complexes in mdx Mice. J Biomed Biotechnol. 2012;2012: 1–8. doi:10.1155/2012/897076
5. Betts C, Saleh AF, Arzumanov AA, Hammond SM, Godfrey C, Coursindel T, et al. Pip6-PMO, A New Generation of Peptide-oligonucleotide Conjugates With Improved Cardiac Exon Skipping Activity for DMD Treatment. Mol Ther — Nucleic Acids. 2012;1: e38. doi:10.1038/mtna.2012.30
6. Betts CA, Saleh AF, Carr CA, Hammond SM, Coenen-Stass AML, Godfrey C, et al. Prevention of exercised induced cardiomyopathy following Pip-PMO treatment in dystrophic mdx mice. Sci Rep. England; 2015;5: 8986. doi:10.1038/srep08986
7. Betts CA, Saleh AF, Carr CA, Muses S, Wells KE, Hammond SM, et al. Implications for Cardiac Function Following Rescue of the Dystrophic Diaphragm in a Mouse Model of Duchenne Muscular Dystrophy. Sci Rep. Nature Publishing Group; 2015;5: 11632. doi:10.1038/srep11632
8. Bish LT, Sleeper MM, Forbes SC, Wang B, Reynolds C, Singletary GE, et al. Long-term Restoration of Cardiac Dystrophin Expression in Golden Retriever Muscular Dystrophy Following rAAV6-mediated Exon Skipping. Mol Ther. 2012;20: 580–589. doi:10.1038/mt.2011.264
9. Bremmer-Bout M, Aartsma-Rus A, de Meijer EJ, Kaman WE, Janson AA, Vossen RH, et al. Targeted Exon Skipping in Transgenic hDMD Mice: A Model for Direct Preclinical Screening of Human-Specific Antisense Oligonucleotides. Mol Ther. 2004;10: 232–240. doi:10.1016/j.ymthe.2004.05.031
10. Burki U, Keane J, Blain A, O'Donovan L, Gait MJ, Laval SH, et al. Development and Application of an Ultrasensitive Hybridization-Based ELISA Method for the Determination of Peptide-Conjugated Phosphorodiamidate Morpholino Oligonucleotides. Nucleic Acid Ther. 2015;25: 275–284. doi:10.1089/nat.2014.0528
11. Chiper M, Tounsi N, Kole R, Kichler A, Zuber G. Self-aggregating 1.8kDa polyethylenimines with dissolution switch at endosomal acidic pH are delivery carriers for plasmid DNA, mRNA, siRNA and exon-skipping oligonucleotides. J Control Release. Elsevier B.V.; 2017;246: 60–70. doi:10.1016/j.jconrel.2016.12.005
12. Coady TH, Baughan TD, Shababi M, Passini MA, Lorson CL. Development of a Single Vector System that Enhances Trans-Splicing of SMN2 Transcripts. Valcarcel J, editor. PLoS One. 2008;3: e3468. doi:10.1371/journal.pone.0003468
13. Coenen-Stass AML, McClorey G, Manzano R, Betts CA, Blain A, Saleh AF, et al. Identification of novel, therapy-responsive protein biomarkers in a mouse model of Duchenne muscular dystrophy by aptamer-based serum proteomics. Sci Rep. 2015;5: 17014. doi:10.1038/srep17014
14. Crisp A, Yin H, Goyenvalle A, Betts C, Moulton HM, Seow Y, et al. Diaphragm rescue alone prevents heart dysfunction in dystrophic mice. Hum Mol Genet. 2011;20: 413–421. doi:10.1093/hmg/ddq477

15. Dabertrand F, Mironneau J, Henaff M, Macrez N, Morel J-L. Comparison between gentamycin and exon skipping treatments to restore ryanodine receptor subtype 2 functions in mdx mouse duodenum myocytes. Eur J Pharmacol. 2010;628: 36–41. doi:10.1016/j.ejphar.2009.11.034
16. Dal Mas A, Rogalska ME, Bussani E, Pagani F. Improvement of SMN2 pre-mRNA processing mediated by exon-specific U1 small nuclear RNA. Am J Hum Genet. United States; 2015;96: 93–103. doi:10.1016/j.ajhg.2014.12.009
17. Dallérac G, Perronnet C, Chagneau C, Leblanc-Veyrac P, Samson-Desvignes N, Peltekian E, et al. Rescue of a dystrophin-like protein by exon skipping normalizes synaptic plasticity in the hippocampus of the mdx mouse. Neurobiol Dis. Elsevier Inc.; 2011;43: 635–641. doi:10.1016/j.nbd.2011.05.012
18. Denti MA, Rosa A, D'Antona G, Sthandier O, De Angelis FG, Nicoletti C, et al. Body-wide gene therapy of Duchenne muscular dystrophy in the mdx mouse model. Proc Natl Acad Sci. 2006;103: 3758–3763. doi:10.1073/pnas.0508917103
19. Denti MA, Rosa A, D'Antona G, Sthandier O, De Angelis FG, Nicoletti C, et al. Chimeric adeno-associated virus/antisense U1 small nuclear RNA effectively rescues dystrophin synthesis and muscle function by local treatment of mdx mice. Hum Gene Ther. United States; 2006;17: 565–574. doi:10.1089/hum.2006.17.565
20. Denti MA, Incitti T, Sthandier O, Nicoletti C, Angelis FG De, Rizzuto E, et al. Long-Term Benefit of Adeno-Associated Virus/Antisense-Mediated Exon Skipping in Dystrophic Mice. Hum Gene Ther. 2008;19: 601–608. doi:10.1089/hum.2008.012
21. Doran P, Wilton SD, Fletcher S, Ohlendieck K. Proteomic profiling of antisense-induced exon skipping reveals reversal of pathobiochemical abnormalities in dystrophic mdx diaphragm. Proteomics. 2009;9: 671–685. doi:10.1002/pmic.200800441
22. Echigoya Y, Aoki Y, Miskew B, Panesar D, Touznik A, Nagata T, et al. Long-Term Efficacy of Systemic Multiexon Skipping Targeting Dystrophin Exons 45–55 With a Cocktail of Vivo-Morpholinos in Mdx52 Mice. Mol Ther — Nucleic Acids. 2015;4: e225. doi:10.1038/mtna.2014.76
23. Echigoya Y, Nakamura A, Nagata T, Urasawa N, Lim KRQ, Trieu N, et al. Effects of systemic multiexon skipping with peptide-conjugated morpholinos in the heart of a dog model of Duchenne muscular dystrophy. Proc Natl Acad Sci. 2017;114: 4213–4218. doi:10.1073/pnas.1613203114
24. Eckenfelder A, Tordo J, Babbs A, Davies KE, Goyenvalle A, Danos O. The Cellular Processing Capacity Limits the Amounts of Chimeric U7 snRNA Available for Antisense Delivery. Mol Ther — Nucleic Acids. 2012;1: e31. doi:10.1038/mtna.2012.24
25. Falzarano MS, Passarelli C, Bassi E, Fabris M, Perrone D, Sabatelli P, et al. Biodistribution and Molecular Studies on Orally Administered Nanoparticle-AON Complexes Encapsulated with Alginate Aiming at Inducing Dystrophin Rescue in mdx Mice. Biomed Res Int. 2013;2013: 1–13. doi:10.1155/2013/527418
26. Ferlini A, Sabatelli P, Fabris M, Bassi E, Falzarano S, Vattemi G, et al. Dystrophin restoration in skeletal, heart and skin arrector pili smooth muscle of mdx mice by ZM2 NP–AON complexes. Gene Ther. England; 2010;17: 432–438. doi:10.1038/gt.2009.145
27. Fletcher S, Honeyman K, Fall AM, Harding PL, Johnsen RD, Wilton SD. Dystrophin expression in the mdx mouse after localised and systemic administration of a morpholino antisense oligonucleotide. J Gene Med. 2006;8: 207–216. doi:10.1002/jgm.838
28. Fletcher S, Honeyman K, Fall AM, Harding PL, Johnsen RD, Steinhaus JP, et al. Morpholino Oligomer–Mediated Exon Skipping Averts the Onset of Dystrophic Pathology in the mdx Mouse. Mol Ther. 2007;15: 1587–1592. doi:10.1038/sj.mt.6300245
29. Gao X, Zhao J, Han G, Zhang Y, Dong X, Cao L, et al. Effective Dystrophin Restoration by a Novel Muscle-Homing Peptide–Morpholino Conjugate in Dystrophin-Deficient mdx Mice. Mol Ther.

2014;22: 1333–1341. doi:10.1038/mt.2014.63
30. Gebski BL, Mann CJ, Fletcher S, Wilton SD. Morpholino antisense oligonucleotide induced dystrophin exon 23 skipping in mdx mouse muscle. Hum Mol Genet. 2003;12: 1801–1811. doi:10.1093/hmg/ddg196
31. Godfrey C, Muses S, McClorey G, Wells KE, Coursindel T, Terry RL, et al. How much dystrophin is enough: the physiological consequences of different levels of dystrophin in the mdx mouse. Hum Mol Genet. 2015;24: 4225–4237. doi:10.1093/hmg/ddv155
32. Goyenvalle A, Vulin A, Fougerousse F, Leturcq F, Kaplan J-C, Garcia L, et al. Rescue of Dystrophic Muscle Through U7 snRNA-Mediated Exon Skipping. Science (80-). 2004;306: 1796–1799. doi:10.1126/science.1104297
33. Goyenvalle A, Babbs A, van Ommen G-JB, Garcia L, Davies KE. Enhanced Exon-skipping Induced by U7 snRNA Carrying a Splicing Silencer Sequence: Promising Tool for DMD Therapy. Mol Ther. 2009;17: 1234–1240. doi:10.1038/mt.2009.113
34. Goyenvalle A, Babbs A, Powell D, Kole R, Fletcher S, Wilton SD, et al. Prevention of Dystrophic Pathology in Severely Affected Dystrophin/Utrophin-deficient Mice by Morpholino-oligomer-mediated Exon-skipping. Mol Ther. Nature Publishing Group; 2010;18: 198–205. doi:10.1038/mt.2009.248
35. Goyenvalle A, Babbs A, Wright J, Wilkins V, Powell D, Garcia L, et al. Rescue of severely affected dystrophin/utrophin-deficient mice through scAAV-U7snRNA-mediated exon skipping. Hum Mol Genet. 2012;21: 2559–2571. doi:10.1093/hmg/dds082
36. Goyenvalle A, Wright J, Babbs A, Wilkins V, Garcia L, Davies KE. Engineering Multiple U7snRNA Constructs to Induce Single and Multiexon-skipping for Duchenne Muscular Dystrophy. Mol Ther. Nature Publishing Group; 2012;20: 1212–1221. doi:10.1038/mt.2012.26
37. Graham IR, Hill VJ, Manoharan M, Inamati GB, Dickson G. Towards a therapeutic inhibition of dystrophin exon 23 splicing inmdx mouse muscle induced by antisense oligoribonucleotides (splicomers): target sequence optimisation using oligonucleotide arrays. J Gene Med. 2004;6: 1149–1158. doi:10.1002/jgm.603
38. Hammond SM, Hazell G, Shabanpoor F, Saleh AF, Bowerman M, Sleigh JN, et al. Systemic peptide-mediated oligonucleotide therapy improves long-term survival in spinal muscular atrophy. Proc Natl Acad Sci. 2016;113: 10962–10967. doi:10.1073/pnas.1605731113
39. Han G, Gu B, Cao L, Gao X, Wang Q, Seow Y, et al. Hexose enhances oligonucleotide delivery and exon skipping in dystrophin-deficient mdx mice. Nat Commun. Nature Publishing Group; 2016;7: 10981. doi:10.1038/ncomms10981
40. Hoen PA ’t, van der Wees CG, Aartsma-Rus A, Turk R, Goyenvalle A, Danos O, et al. Gene expression profiling to monitor therapeutic and adverse effects of antisense therapies for Duchenne muscular dystrophy. Pharmacogenomics. 2006;7: 281–297. doi:10.2217/14622416.7.3.281
41. Ivanova GD, Arzumanov A, Abes R, Yin H, Wood MJA, Lebleu B, et al. Improved cell-penetrating peptide-PNA conjugates for splicing redirection in HeLa cells and exon skipping in mdx mouse muscle. Nucleic Acids Res. 2008;36: 6418–6428. doi:10.1093/nar/gkn671
42. Jearawiriyapaisarn N, Moulton HM, Buckley B, Roberts J, Sazani P, Fucharoen S, et al. Sustained Dystrophin Expression Induced by Peptide-conjugated Morpholino Oligomers in the Muscles of mdx Mice. Mol Ther. 2008;16: 1624–1629. doi:10.1038/mt.2008.120
43. Jearawiriyapaisarn N, Moulton HM, Sazani P, Kole R, Willis MS. Long-term improvement in mdx cardiomyopathy after therapy with peptide-conjugated morpholino oligomers. Cardiovasc Res. 2010;85: 444–453. doi:10.1093/cvr/cvp335
44. Jirka SMG, Heemskerk H, Tanganyika-de Winter CL, Muilwijk D, Pang KH, de Visser PC, et al. Peptide conjugation of 2’-O-methyl phosphorothioate antisense oligonucleotides enhances

cardiac uptake and exon skipping in mdx mice. Nucleic Acid Ther. United States; 2014;24: 25–36. doi:10.1089/nat.2013.0448
45. Kim Y, Tewari M, Pajerowski JD, Cai S, Sen S, Williams J, et al. Polymersome delivery of siRNA and antisense oligonucleotides. J Control Release. 2009;134: 132–140. doi:10.1016/j.jconrel.2008.10.020
46. Koebis M, Kiyatake T, Yamaura H, Nagano K, Higashihara M, Sonoo M, et al. Ultrasound-enhanced delivery of morpholino with Bubble liposomes ameliorates the myotonia of myotonic dystrophy model mice. Sci Rep. 2013;3: 2242. doi:10.1038/srep02242
47. Lee HJ, Boado RJ, Braasch DA, Corey DR, Pardridge WM. Imaging gene expression in the brain in vivo in a transgenic mouse model of Huntington's disease with an antisense radiopharmaceutical and drug-targeting technology. J Nucl Med. United States; 2002;43: 948–956.
48. Leger AJ, Mosquea LM, Clayton NP, Wu I-H, Weeden T, Nelson C a, et al. Systemic Delivery of a Peptide-Linked Morpholino Oligonucleotide Neutralizes Mutant RNA Toxicity in a Mouse Model of Myotonic Dystrophy. Nucleic Acid Ther. 2013;23: 109–117. doi:10.1089/nat.2012.0404
49. Lu QL, Mann CJ, Lou F, Bou-Gharios G, Morris GE, Xue S, et al. Functional amounts of dystrophin produced by skipping the mutated exon in the mdx dystrophic mouse. Nat Med. 2003;9: 1009–1014. doi:10.1038/nm897
50. Lu QL, Rabinowitz A, Chen YC, Yokota T, Yin H, Alter J, et al. Systemic delivery of antisense oligoribonucleotide restores dystrophin expression in body-wide skeletal muscles. Proc Natl Acad Sci. 2005;102: 198–203. doi:10.1073/pnas.0406700102
51. Lu-Nguyen NB, Jarmin SA, Saleh AF, Popplewell L, Gait MJ, Dickson G. Combination Antisense Treatment for Destructive Exon Skipping of Myostatin and Open Reading Frame Rescue of Dystrophin in Neonatal mdx Mice. Mol Ther. 2015;23: 1341–1348. doi:10.1038/mt.2015.88
52. Malerba A, Kang JK, McClorey G, Saleh AF, Popplewell L, Gait MJ, et al. Dual Myostatin and Dystrophin Exon Skipping by Morpholino Nucleic Acid Oligomers Conjugated to a Cell-penetrating Peptide Is a Promising Therapeutic Strategy for the Treatment of Duchenne Muscular Dystrophy. Mol Ther — Nucleic Acids. 2012;1: e62. doi:10.1038/mtna.2012.54
53. Mann CJ, Honeyman K, Cheng AJ, Ly T, Lloyd F, Fletcher S, et al. Antisense-induced exon skipping and synthesis of dystrophin in the mdx mouse. Proc Natl Acad Sci. 2001;98: 42–47. doi:10.1073/pnas.011408598
54. Mitrpant C, Fletcher S, Iversen PL, Wilton SD. By-passing the nonsense mutation in the 4 CV mouse model of muscular dystrophy by induced exon skipping. J Gene Med. 2009;11: 46–56. doi:10.1002/jgm.1265
55. Morcos P, Li Y, Jiang S. Vivo-Morpholinos: A non-peptide transporter delivers Morpholinos into a wide array of mouse tissues. Biotechniques. 2008;45: 613–623. doi:10.2144/000113005
56. Nakamura M, Ando Y, Nagahara S, Sano A, Ochiya T, Maeda S, et al. Targeted conversion of the transthyretin gene in vitro and in vivo. Gene Ther. 2004;11: 838–846. doi:10.1038/sj.gt.3302228
57. Negishi Y, Ishii Y, Shiono H, Akiyama S, Sekine S, Kojima T, et al. Bubble Liposomes and Ultrasound Exposure Improve Localized Morpholino Oligomer Delivery into the Skeletal Muscles of Dystrophic mdx Mice. Mol Pharm. 2014;11: 1053–1061. doi:10.1021/mp4004755
58. Nizzardo M, Simone C, Salani S, Ruepp M-D, Rizzo F, Ruggieri M, et al. Effect of Combined Systemic and Local Morpholino Treatment on the Spinal Muscular Atrophy Delta 7 Mouse Model Phenotype. Clin Ther. 2014;36: 340–356. doi:10.1016/j.clinthera.2014.02.004
59. Pandey SN, Lee Y-C, Yokota T, Chen Y-W. Morpholino Treatment Improves Muscle Function and Pathology of Pitx1 Transgenic Mice. Mol Ther. 2014;22: 390–396. doi:10.1038/mt.2013.263
60. Peccate C, Mollard A, Le Hir M, Julien L, McClorey G, Jarmin S, et al. Antisense pre-treatment increases gene therapy efficacy in dystrophic muscles. Hum Mol Genet. 2016;25: 3555–3563. doi:10.1093/hmg/ddw201

61. Rimessi P, Sabatelli P, Fabris M, Braghetta P, Bassi E, Spitali P, et al. Cationic PMMA Nanoparticles Bind and Deliver Antisense Oligoribonucleotides Allowing Restoration of Dystrophin Expression in the mdx Mouse. Mol Ther. United States; 2009;17: 820–827. doi:10.1038/mt.2009.8
62. Roberts TC, Blomberg KEM, McClorey G, Andaloussi S EL, Godfrey C, Betts C, et al. Expression Analysis in Multiple Muscle Groups and Serum Reveals Complexity in the MicroRNA Transcriptome of the mdx Mouse with Implications for Therapy. Mol Ther — Nucleic Acids. 2012;1: e39. doi:10.1038/mtna.2012.26
63. Rouillon J, Poupiot J, Zocevic A, Amor F, Léger T, Garcia C, et al. Serum proteomic profiling reveals fragments of MYOM3 as potential biomarkers for monitoring the outcome of therapeutic interventions in muscular dystrophies. Hum Mol Genet. 2015;24: 4916–4932. doi:10.1093/hmg/ddv214
64. Shababi M, Glascock J, Lorson CL. Combination of SMN Trans -Splicing and a Neurotrophic Factor Increases the Life Span and Body Mass in a Severe Model of Spinal Muscular Atrophy. Hum Gene Ther. 2011;22: 135–144. doi:10.1089/hum.2010.114
65. Shababi M, Lorson CL. Optimization of SMN Trans-Splicing Through the Analysis of SMN Introns. J Mol Neurosci. 2012;46: 459–469. doi:10.1007/s12031-011-9614-3
66. Shabanpoor F, McClorey G, Saleh AF, Jarver P, Wood MJA, Gait MJ. Bi-specific splice-switching PMO oligonucleotides conjugated via a single peptide active in a mouse model of Duchenne muscular dystrophy. Nucleic Acids Res. 2015;43: 29–39. doi:10.1093/nar/gku1256
67. Shabanpoor F, Hammond SM, Abendroth F, Hazell G, Wood MJA, Gait MJ. Identification of a Peptide for Systemic Brain Delivery of a Morpholino Oligonucleotide in Mouse Models of Spinal Muscular Atrophy. Nucleic Acid Ther. 2017;0: nat.2016.0652. doi:10.1089/nat.2016.0652
68. Sirsi SR, Schray RC, Guan X, Lykens NM, Williams JH, Erney ML, et al. Functionalized PEG–PEI Copolymers Complexed to Exon-Skipping Oligonucleotides Improve Dystrophin Expression in mdx Mice. Hum Gene Ther. 2008;19: 795–806. doi:10.1089/hum.2007.129
69. Sirsi SR, Schray RC, Wheatley MA, Lutz GJ. Formulation of polylactide-co-glycolic acid nanospheres for encapsulation and sustained release of poly(ethylene imine)-poly(ethylene glycol) copolymers complexed to oligonucleotides. J Nanobiotechnology. 2009;7: 1. doi:10.1186/1477-3155-7-1
70. Taniguchi-Ikeda M, Kobayashi K, Kanagawa M, Yu C, Mori K, Oda T, et al. Pathogenic exon-trapping by SVA retrotransposon and rescue in Fukuyama muscular dystrophy. Nature. 2011;478: 127–131. doi:10.1038/nature10456
71. Vaillend C, Perronnet C, Ros C, Gruszczynski C, Goyenvalle A, Laroche S, et al. Rescue of a Dystrophin-like Protein by Exon Skipping In Vivo Restores GABAA-receptor Clustering in the Hippocampus of the mdx Mouse. Mol Ther. Nature Publishing Group; 2010;18: 1683–1688. doi:10.1038/mt.2010.134
72. Vulin A, Barthélémy I, Goyenvalle A, Thibaud J-L, Beley C, Griffith G, et al. Muscle Function Recovery in Golden Retriever Muscular Dystrophy After AAV1-U7 Exon Skipping. Mol Ther. 2012;20: 2120–2133. doi:10.1038/mt.2012.181
73. Wang Q, Yin H, Camelliti P, Betts C, Moulton H, Lee H, et al. In vitro evaluation of novel antisense oligonucleotides is predictive of in vivo exon skipping activity for Duchenne muscular dystrophy. J Gene Med. 2010;12: 354–364. doi:10.1002/jgm.1446
74. Wang M, Wu B, Lu P, Cloer C, Tucker JD, Lu Q. Polyethylenimine-modified Pluronics (PCMs) Improve Morpholino Oligomer Delivery in Cell Culture and Dystrophic mdx Mice. Mol Ther. 2013;21: 210–216. doi:10.1038/mt.2012.236
75. Wang M, Wu B, Lu P, Tucker JD, Milazi S, Shah SN, et al. Pluronic-PEI copolymers enhance exon-skipping of 2'-O-methyl phosphorothioate oligonucleotide in cell culture and dystrophic mdx mice. Gene Ther. England; 2014;21: 52–59. doi:10.1038/gt.2013.57

76. Wang M, Wu B, Tucker JD, Lu P, Cloer C, Lu QL. Evaluation of Tris[2-(Acryloyloxy)Ethyl]Isocyanurate Cross-Linked Polyethylenimine as Antisense Morpholino Oligomer Delivery Vehicle in Cell Culture and Dystrophic mdx Mice. Hum Gene Ther. 2014;25: 419–427. doi:10.1089/hum.2013.156
77. Wang M, Wu B, Tucker JD, Lu P, Bollinger LE, Lu Q. Tween 85 grafted PEIs enhanced delivery of antisense 2′-O-methyl phosphorothioate oligonucleotides in vitro and in dystrophic mdx mice. J Mater Chem B. 2015;3: 5330–5340. doi:10.1039/C5TB00139K
78. Wang M, Wu B, Tucker JD, Lu P, Lu Q. Cationic polyelectrolyte-mediated delivery of antisense morpholino oligonucleotides for exon-skipping in vitro and in mdx mice. Int J Nanomedicine. 2015;10: 5635–5646. doi:10.2147/IJN.S89910
79. Wang M, Wu B, Tucker JD, Bollinger LE, Lu P, Lu Q. Poly(ester amine) Composed of Polyethylenimine and Pluronic Enhance Delivery of Antisense Oligonucleotides In Vitro and in Dystrophic mdx Mice. Mol Ther - Nucleic Acids. 2016;5: e341. doi:10.1038/mtna.2016.51
80. Widrick JJ, Jiang S, Choi SJ, Knuth ST, Morcos PA. An octaguanidine-morpholino oligo conjugate improves muscle function of mdx mice. Muscle Nerve. 2011;44: 563–570. doi:10.1002/mus.22126
81. Williams JH, Sirsi SR, Latta DR, Lutz GJ. Induction of Dystrophin Expression by Exon Skipping in mdx Mice Following Intramuscular Injection of Antisense Oligonucleotides Complexed with PEG–PEI Copolymers. Mol Ther. 2006;14: 88–96. doi:10.1016/j.ymthe.2005.11.025
82. Williams JH, Schray RC, Sirsi SR, Lutz GJ. Nanopolymers improve delivery of exon skipping oligonucleotides and concomitant dystrophin expression in skeletal muscle of mdx mice. BMC Biotechnol. 2008;8: 35. doi:10.1186/1472-6750-8-35
83. Wu B, Moulton HM, Iversen PL, Jiang J, Li J, Li J, et al. Effective rescue of dystrophin improves cardiac function in dystrophin-deficient mice by a modified morpholino oligomer. Proc Natl Acad Sci. 2008;105: 14814–14819. doi:10.1073/pnas.0805676105
84. Wu B, Li Y, Morcos PA, Doran TJ, Lu P, Lu QL. Octa-guanidine Morpholino Restores Dystrophin Expression in Cardiac and Skeletal Muscles and Ameliorates Pathology in Dystrophic mdx Mice. Mol Ther. 2009;17: 864–871. doi:10.1038/mt.2009.38
85. Wu B, Lu P, Cloer C, Shaban M, Grewal S, Milazi S, et al. Long-Term Rescue of Dystrophin Expression and Improvement in Muscle Pathology and Function in Dystrophic mdx Mice by Peptide-Conjugated Morpholino. Am J Pathol. 2012;181: 392–400. doi:10.1016/j.ajpath.2012.04.006
86. Wu B, Cloer C, Lu P, Milazi S, Shaban M, Shah SN, et al. Exon skipping restores dystrophin expression, but fails to prevent disease progression in later stage dystrophic dko mice. Gene Ther. 2014;21: 785–793. doi:10.1038/gt.2014.53
87. Yin H, Lu Q, Wood M. Effective Exon Skipping and Restoration of Dystrophin Expression by Peptide Nucleic Acid Antisense Oligonucleotides in mdx Mice. Mol Ther. 2008;16: 38–45. doi:10.1038/sj.mt.6300329
88. Yin H, Moulton HM, Seow Y, Boyd C, Boutilier J, Iverson P, et al. Cell-penetrating peptide-conjugated antisense oligonucleotides restore systemic muscle and cardiac dystrophin expression and function. Hum Mol Genet. 2008;17: 3909–3918. doi:10.1093/hmg/ddn293
89. Yin H, Moulton HM, Betts C, Seow Y, Boutilier J, Iverson PL, et al. A fusion peptide directs enhanced systemic dystrophin exon skipping and functional restoration in dystrophin-deficient mdx mice. Hum Mol Genet. 2009;18: 4405–4414. doi:10.1093/hmg/ddp395
90. Yin H, Moulton HM, Betts C, Merritt T, Seow Y, Ashraf S, et al. Functional rescue of dystrophin-deficient mdx mice by a chimeric peptide-PMO. Mol Ther. United States; 2010;18: 1822–1829. doi:10.1038/mt.2010.151
91. Yin H, Betts C, Saleh AF, Ivanova GD, Lee H, Seow Y, et al. Optimization of peptide nucleic acid

antisense oligonucleotides for local and systemic dystrophin splice correction in the mdx mouse. Mol Ther. United States; 2010;18: 819–827. doi:10.1038/mt.2009.310

92. Yin H, Saleh AF, Betts C, Camelliti P, Seow Y, Ashraf S, et al. Pip5 transduction peptides direct high efficiency oligonucleotide-mediated dystrophin exon skipping in heart and phenotypic correction in mdx mice. Mol Ther. United States; 2011;19: 1295–1303. doi:10.1038/mt.2011.79
93. Yin H, Boisguerin P, Moulton HM, Betts C, Seow Y, Boutilier J, et al. Context Dependent Effects of Chimeric Peptide Morpholino Conjugates Contribute to Dystrophin Exon-skipping Efficiency. Mol Ther — Nucleic Acids. 2013;2: e124. doi:10.1038/mtna.2013.51
94. Yokota T, Nakamura A, Nagata T, Saito T, Kobayashi M, Aoki Y, et al. Extensive and prolonged restoration of dystrophin expression with vivo-morpholino-mediated multiple exon skipping in dystrophic dogs. Nucleic Acid Ther. 2012;22: 306–315. doi:10.1089/nat.2012.0368
95. Zhou H, Janghra N, Mitrpant C, Dickinson RL, Anthony K, Price L, et al. A Novel Morpholino Oligomer Targeting ISS-N1 Improves Rescue of Severe Spinal Muscular Atrophy Transgenic Mice. Hum Gene Ther. 2013;24: 331–342. doi:10.1089/hum.2012.211

File S2. PRISMA checklist.

PRISMA 2009 Checklist

Section/topic	#	Checklist item	Reported on page #
**TITLE**			
Title	1	Identify the report as a systematic review, meta-analysis, or both.	1
**ABSTRACT**			
Structured summary	2	Provide a structured summary including, as applicable: background; objectives; data sources; study eligibility criteria, participants, and interventions; study appraisal and synthesis methods; results; limitations; conclusions and implications of key findings; systematic review registration number.	2
**INTRODUCTION**			
Rationale	3	Describe the rationale for the review in the context of what is already known.	3-7
Objectives	4	Provide an explicit statement of questions being addressed with reference to participants, interventions, comparisons, outcomes, and study design (PICOS).	7
**METHODS**			
Protocol and registration	5	Indicate if a review protocol exists, if and where it can be accessed (e.g., Web address), and, if available, provide registration information including registration number.	24
Eligibility criteria	6	Specify study characteristics (e.g., PICOS, length of follow-up) and report characteristics (e.g., years considered, language, publication status) used as criteria for eligibility, giving rationale.	24
Information sources	7	Describe all information sources (e.g., databases with dates of coverage, contact with study authors to identify additional studies) in the search and date last searched.	24
Search	8	Present full electronic search strategy for at least one database, including any limits used, such that it could be repeated.	Supplementary File S3
Study selection	9	State the process for selecting studies (i.e., screening, eligibility, included in systematic review, and, if applicable, included in the meta-analysis).	24-25
Data collection process	10	Describe method of data extraction from reports (e.g., piloted forms, independently, in duplicate) and any processes for obtaining and confirming data from investigators.	25
Data items	11	List and define all variables for which data were sought (e.g., PICOS, funding sources) and any assumptions and simplifications made.	25-27; Supplementary Table S1
Risk of bias in individual studies	12	Describe methods used for assessing risk of bias of individual studies (including specification of whether this was done at the study or outcome level), and how this information is to be used in any data synthesis.	25; Supplementary Table S2
Summary measures	13	State the principal summary measures (e.g., risk ratio, difference in means).	26-27
Synthesis of results	14	Describe the methods of handling data and combining results of studies, if done, including measures of consistency (e.g., I^2) for each meta-analysis.	27

PRISMA 2009 Checklist

Section/topic	#	Checklist item	Reported on page #
Risk of bias across studies	15	Specify any assessment of risk of bias that may affect the cumulative evidence (e.g., publication bias, selective reporting within studies).	25
Additional analyses	16	Describe methods of additional analyses (e.g., sensitivity or subgroup analyses, meta-regression), if done, indicating which were pre-specified.	-
**RESULTS**			
Study selection	17	Give numbers of studies screened, assessed for eligibility, and included in the review, with reasons for exclusions at each stage, ideally with a flow diagram.	8; Fig 1
Study characteristics	18	For each study, present characteristics for which data were extracted (e.g., study size, PICOS, follow-up period) and provide the citations.	8-14; Supplementary Table S1
Risk of bias within studies	19	Present data on risk of bias of each study and, if available, any outcome level assessment (see item 12).	15; Supplementary Table S2
Results of individual studies	20	For all outcomes considered (benefits or harms), present, for each study: (a) simple summary data for each intervention group (b) effect estimates and confidence intervals, ideally with a forest plot.	15-16; Fig 6
Synthesis of results	21	Present results of each meta-analysis done, including confidence intervals and measures of consistency.	15-16
Risk of bias across studies	22	Present results of any assessment of risk of bias across studies (see Item 15).	15
Additional analysis	23	Give results of additional analyses, if done (e.g., sensitivity or subgroup analyses, meta-regression [see Item 16]).	-
**DISCUSSION**			
Summary of evidence	24	Summarize the main findings including the strength of evidence for each main outcome; consider their relevance to key groups (e.g., healthcare providers, users, and policy makers).	17-22
Limitations	25	Discuss limitations at study and outcome level (e.g., risk of bias), and at review-level (e.g., incomplete retrieval of identified research, reporting bias).	17-22
Conclusions	26	Provide a general interpretation of the results in the context of other evidence, and implications for future research.	23
**FUNDING**			
Funding	27	Describe sources of funding for the systematic review and other support (e.g., supply of data); role of funders for the systematic review.	36

From: Moher D, Liberati A, Tetzlaff J, Altman DG, The PRISMA Group (2009). Preferred Reporting Items for Systematic Reviews and Meta-Analyses: The PRISMA Statement. PLoS Med 6(7): e1000097. doi:10.1371/journal.pmed1000097

For more information, visit: **www.prisma-statement.org**.

File S3. Full search strategies for Embase, Pubmed and Web of Science

1. Embase

Search component #1: Intervention

((cell penetrating peptide/ OR exp drug carrier/ OR liposomal delivery/ OR exp nanoparticle/ OR nanopharmaceutics/) OR (AAV OR Adeno-associated OR Cationic lipid OR Cationic lipids OR Cell-penetrating peptide OR Cell-penetrating peptides OR Chitosan OR Conjugate OR Conjugated OR Conjugates OR Conjugation OR Copolymer OR Co-polymer OR Co-polymers OR Copolymers OR CPP OR CPPs OR Dendrimer OR Dendrimers OR Dendriplex OR Dendriplexes OR Excipient OR Excipients OR Formulation OR Formulations OR Lipid OR Lipids OR Lipofectamine OR Lipofection OR Lipoplex OR Lipoplexes OR liposome OR liposomes OR Nanoballs OR Nanocapsule OR Nanocapsules OR Nanocarrier OR Nanocarriers OR Nanocomposite OR Nanocomposites OR Nanoconjugate OR Nanoconjugates OR Nanocrystal OR Nanocrystals OR nanoparticle OR nanoparticles OR Nanoparticulate OR Nanorod OR Nanorods OR Nanotube OR Nanotubes OR Nano-ball OR Nano-balls OR Nano-capsule OR Nano-capsules OR Nano-carrier OR Nano-carriers OR Nano-composite OR Nano-composites OR Nano-conjugate OR Nano-conjugates OR Nano-crystal OR Nano-crystals OR Nano-particle OR Nano-particles OR Nano-particulate OR Nano-rod OR Nano-rods OR Nano-tube OR Nano-tubes OR Oligoplex OR Oligoplexes OR PEI OR peptide OR peptide-linked OR peptides OR PLGA OR Polyelectrolyte OR Polyelectrolytes OR Polyethylenimine OR polylactic-co-glycolic acid OR Polymer OR Polymers OR Polymersomes OR Polyplex OR Polyplexes OR Protamine OR Silica OR Thiomer OR Thiomers OR Transfection OR Vector OR Vectorized OR Vectors OR Viral OR Virosome OR Virosomes OR Virus OR Viruses OR Virus-like particle OR Virus-like particles OR VLP OR VLPs).ti,ab,kw.) AND ((exp antisense oligonucleotide/ OR locked nucleic acid/ OR morpholino oligonucleotide/ OR oligonucleotide phosphorothioate/ OR peptide nucleic acid/) OR (antisense OR AON OR AONs OR ASO OR ASOs OR LNA OR LNAs OR Locked nucleic acid OR Locked nucleic acids OR MORF OR MORFs OR Morpholino OR Morpholinos OR Peptide nucleic acid OR Peptide nucleic acids OR Phosphorothioate OR Phosphorothioates OR PMO OR PMOs OR PNA OR PNAs OR SCO OR SCOs OR splice-correcting oligonucleotide OR splice-correcting oligonucleotides OR splice-switching oligonucleotide OR splice-switching oligonucleotides OR SSO OR SSOs).ti,ab,kw.)

Search component #2: Disease

(adrenoleukodystrophy/ OR Alexander disease/ OR Alstrom syndrome/ OR Alzheimer Disease/ OR amyloid neuropathy/ OR amyotrophic lateral sclerosis/ OR Ataxia Telangiectasia/ OR becker muscular dystrophy/ OR bulbar paralysis/ OR Canavan disease/ OR central core disease/ OR cerebellum dyssynergia/ OR chronic progressive external ophthalmoplegia/ OR Cockayne syndrome/ OR congenital analgesia/ OR diffuse Lewy body disease/ OR distal myopathy/ OR dysautonomia/ OR fatal familial insomnia/ OR Friedreich ataxia/ OR frontotemporal dementia/ OR Gerstmann Straussler Scheinker syndrome/ OR giant axonal neuropathy/ OR Gilles de la Tourette syndrome/ OR globoid cell leukodystrophy/ OR glycogen storage disease type 7/ OR hereditary motor sensory neuropathy/ OR hereditary optic atrophy/ OR Huntington chorea/ OR hypokalemic periodic paralysis/ OR inclusion body myositis/ OR Kearns Sayre syndrome/ OR Kennedy disease/ OR Luft disease/ OR Machado Joseph disease/ OR MELAS syndrome/ OR MERRF syndrome/ OR metachromatic leukodystrophy/ OR mitochondrial encephalomyopathy/ OR mitochondrial myopathy/ OR Motor Neuron Disease/ OR muscular dystrophy/ OR Myasthenia Gravis/ OR myoclonus epilepsy/ OR myokymia/ OR Myositis/ OR myotonic dystrophy/ OR nemaline myopathy/ OR neural ceroid lipofuscinosis/ OR neurodegeneration with brain

iron accumulation/ OR neurofibromatosis/ OR olivopontocerebellar atrophy/ OR ossifying myositis/ OR Parkinson Disease/ OR Pelizaeus Merzbacher disease/ OR periodic paralysis/ OR peripheral neuropathy/ OR Pick presenile dementia/ OR polyneuropathy/ OR progressive nonfluent aphasia/ OR Refsum disease/ OR sarcoglycanopathy/ OR Shy Drager syndrome/ OR spinal muscular atrophy/ OR spinocerebellar degeneration/ OR striatonigral degeneration/ OR Tangier disease/ OR tauopathy/ OR TDP 43 proteinopathy/ OR Thomsen disease/ OR torsion dystonia/ OR tuberous sclerosis/ OR Wilson disease/ OR Wolfram syndrome/ OR congenital myasthenic syndrome/) OR (((autosomal OR congenital OR dominant OR familial OR genealogical OR genetic OR hereditary OR heritable OR inborn OR inheritable OR inheritance OR inherited OR recessive OR single-gene OR X-linked).ti,ab,kw.) AND (((degenerative AND nervous system) OR (degenerative AND neurologic) OR (degenerative AND neurological) OR (degenerative AND spinal cord) OR fasciculation OR neurodegenerative OR neuromuscular).ti,ab,kw.)) OR (("Adenoma Sebaceum" OR "adreno leucodystrophy" OR "adreno leukodystrophy" OR "adrenoleucodystrophy" OR "Adrenoleukodystrophy" OR "adrenoleukomyeloneuropathy" OR "adrenomyeloneuropathy" OR "Alexander Disease" OR "alexander syndrome" OR "Alexanders Disease" OR "Alexander's Disease" OR "Alstrom Hallgren Syndrome" OR "Alstrom Syndrome" OR "Alstrom-Hallgren Syndrome" OR "Alstroms Syndrome" OR "Alstrom's Syndrome" OR "Alzeimer's" OR "Alzheimer" OR "Alzheimers" OR "amaurotic familial idiocy" OR "amaurotic idiocy" OR "Aminoacylase 2 Deficiency" OR "amyloid neuropathies" OR "Amyloid Neuropathy" OR "Amyloid Polyneuropathies" OR "amyloid polyneuropathy" OR "amyloidotic neuropathy" OR "amyloidotic polyneuropathy" OR "Amyotrophic Lateral Sclerosis" OR "analgesia congenita" OR "Analphalipoproteinaemia" OR "Analphalipoproteinemia" OR "Anterior Horn Cell Disease" OR "ARSA Deficiency" OR "Arylsulfatase A Deficiencies" OR "Arylsulfatase A Deficiency" OR "ASP Deficiency" OR "ASPA Deficiency" OR "Aspartoacylase Deficiency" OR "Ataxia Telangiectasia Syndrome" OR "Ataxia Telangiectasia" OR "ataxia telangiectatica" OR "ataxia teleangiectasia" OR "Autosomal Dominant Cerebellar Ataxia, Type II" OR "Autosomal Dominant Optic Atrophy" OR "Autosomal Dominant Spastic Paraplegia" OR "Autosomal Dominant Striatonigral Degeneration" OR "Autosomal Recessive Spastic Paraplegia" OR "Azorean Disease" OR "balo disease" OR "Baltic Myoclonus" OR "bar syndrome" OR "Batten" OR "Batten-Turner" OR "Becker Disease" OR "Becker Muscular Dystrophy" OR "Becker's Muscular Dystrophy" OR "beta-Galactosidase Deficiencies" OR "beta-Galactosidase Deficiency" OR "Bilateral Acoustic Neurinoma" OR "Bilateral Acoustic Neurinomas" OR "Bilateral Acoustic Neuroma" OR "Bilateral Acoustic Neuromas" OR "Bilateral Acoustic Schwannoma" OR "Bilateral Acoustic Schwannomas" OR "bourneville disease" OR "Bourneville Phakomatosis" OR "bourneville pringle syndrome" OR "Bourneville Pringle's Disease" OR "bourneville syndrome" OR "bourneville tuberous sclerosis" OR "Bourneville-Pringle Disease" OR "Bourneville-Pringles Disease" OR "Bourneville-Pringle's Disease" OR "Bourneville's Disease" OR "Bourneville's Syndrome" OR "brain sclerosis" OR "bulbar and spinal muscular atrophy" OR "bulbar dysfunction" OR "bulbar muscle weakness" OR "Bulbar palsies" OR "Bulbar Palsy" OR "bulbar paralysis" OR "bulbar sign" OR "bulbar symptom" OR "bulbar syndrome" OR "bulbar weakness" OR "Bulbospinal atrophy" OR "Bulbo-Spinal Atrophy" OR "bulbospinal muscular atrophy" OR "bulbo-spinal muscular atrophy" OR "Bulbospinal Neuronopathy" OR "Canavan Disease" OR "canavan syndrome" OR "Canavan's disease" OR "Canavan-van Bogaert-Bertrand Disease" OR "Central core disease" OR "Central Core Diseases" OR "Central Core Myopathies" OR "Central Core Myopathy" OR "Cerebellar Degeneration with Slow Eye Movements" OR "cerebellar dyssynergia" OR "Cerebral Scleroses" OR "cerebral sclerosis" OR "Cerebroside Sulphatase Deficiency" OR "Ceroid Storage Disease" OR "Ceroid Storage Diseases" OR "Channelopathy-Associated Insensitivity To Pain" OR "Charcot Disease" OR "charcot marie disease" OR "Charcot Marie Tooth" OR "Childhood Torsion Disease" OR "Cholesterol Thesaurismosis" OR "chorea major" OR "chronic progressive chorea" OR "Chronic Progressive External Ophthalmoplegia" OR "Chronic Progressive Hereditary Chorea" OR "Circumscribed Lobar Atrophy of the Brain" OR "Cockayne Syndrome" OR "Cockayne" OR "Cockayne's" OR "concentric sclerosis" OR "congenital anaesthesia" OR "congenital analgesia" OR "congenital anesthesia" OR "congenital atonic sclerotic muscular dystrophy" OR "congenital generalized analgesia" OR "congenital indifference to pain" OR "congenital insensitivity to pain" OR "Congenital Myopathy" OR "Congenital Myotonia" OR "congenital pain indifference" OR "Congenital Pain Insensitivity" OR

"congenital paramyotonia" OR "Continuous Muscle Activity Syndrome" OR "Copper Storage Disease" OR "CPEO with Ragged Red Fibers" OR "cranial nerve paralysis" OR "DDPAC" OR "degeneratio hepato lenticularis" OR "dejerine sottas" OR "Dejerine-Thomas" OR "dementia with Lewy bodies" OR "dementia with Lewy body" OR "Dementia with Lobar Atrophy and Neuronal Cytoplasmic Inclusions" OR "DIDMOAD" OR "Dilated Cardiomyopathy" OR "Disinhibition-Dementia-Parkinsonism-Amyotrophy" OR "Distal 1 Myopathy" OR "Distal Muscular Dystrophies" OR "Distal Muscular Dystrophy" OR "Distal Myopathies" OR "Distal Myopathy" OR "Dominant Hereditary Sensory Neuropathy" OR "Dominant Optic Atrophies" OR "dominant optic atrophy" OR "Duchenne" OR "dysantonomia" OR "Dysmyelinogenic Leukodystrophy" OR "Dyssynergia Cerebellaris " OR "Dystonia Musculorum Deformans" OR "dystrophia myotonica" OR "dystrophic myotonia" OR "Emery-Dreifuss" OR "encephalitis periaxialis" OR "epiloia" OR "Eulenburg" OR "Facioscapulohumeral" OR "Facio-Scapulo-Humeral" OR "Facioscapuloperoneal" OR "familial autonomic dysfunction" OR "Familial dysautonomia" OR "familial dysautonomy" OR "Familial Fatal Insomnia" OR "Familial Periodic Paralyses" OR "Familial Periodic Paralysis" OR "Familial Polyneuropathies" OR "Familial Polyneuropathy" OR "familial spastic paralysis" OR "familial spastic paraparesis" OR "familial spastic paraplegia" OR "Fatal Familial Insomnia" OR "Fazio-Londe Disease" OR "Fazio-Londe Syndrome" OR "Fibrinoid Degeneration of Astrocytes" OR "Fibrodysplasia Ossificans" OR "Friedreich Ataxia" OR "Friedreich" OR "Friedreich’s" OR "Friedreichs" OR "frontal dementia" OR "frontal lobe dementia" OR "Frontotemporal Dementia" OR "frontotemporal dementias" OR "Frontotemporal Lobar Degeneration" OR "Frontotemporal Lobe Dementia" OR "FTD" OR "FTDP" OR "FTLD" OR "Fukuhara disease" OR "Galactocerebrosidase Deficiency" OR "Galactosylceramidase Deficiency" OR "galactosylceramide lipidosis" OR "GALC Deficiency" OR "Gehrig Disease" OR "Gehrigs Disease" OR "Gehrig's Disease" OR "Generalized Myotonia" OR "Generalized Myotonias" OR "Gerstmann-Straussler" OR "Gerstmann-Straussler-Scheinker Disease" OR "Giant Axonal Neuropathy" OR "globoid cell dystrophy" OR "globoid cell leucodystrophy" OR "Globoid Cell Leukodystrophy" OR "Globoid Leukodystrophies" OR "Globoid Leukodystrophy" OR "Glycogen Storage Disease Type VII" OR "Glycogen Storage Disease VII" OR "Graefe's Disease" OR "Greenfield Disease" OR "Greenfield's Disease" OR "GSD VII" OR "GSS" OR "Guam Disease" OR "Hallervorden Spatz" OR "Hallervorden-Spatz" OR "Hauptmann-Thannhauser" OR "HDDD1" OR "HDDD2" OR "hemimegalencephaly" OR "hepatocerebral degeneration" OR "Hepatocerebral Degenerations" OR "Hepatolenticular Degeneration" OR "hepatolenticular syndrome" OR "Hereditary and Autonomic Neuropathy" OR "Hereditary Areflexic Dystasia" OR "hereditary atactic polyneuritis" OR "hereditary chorea" OR "Hereditary Dysphasic Disinhibition Dementia" OR "hereditary motor and sensory neuropathies" OR "Hereditary Motor and Sensory Neuropathy " OR "Hereditary Motor and Sensory Neuropathy 5" OR "Hereditary Motor and Sensory Neuropathy Type IV" OR "Hereditary Motor And Sensory Neuropathy V" OR "Hereditary Motor Neuronopathies" OR "Hereditary Motor Neuronopathy" OR "hereditary motor sensory neuropathy type IV" OR "hereditary motor sensory neuropathy" OR "hereditary myotonia" OR "Hereditary Optic Atrophies" OR "Hereditary Optic Atrophy" OR "hereditary optic nerve atrophy" OR "Hereditary Optic Neuroretinopathy" OR "hereditary sensorimotor neuropathy" OR "Hereditary Sensory and Autonomic Neuropathy" OR "hereditary sensory and motor neuropathy" OR "Hereditary Sensory Neuropathy" OR "hereditary spastic paralysis" OR "hereditary spastic paraparesis" OR "Hereditary Spastic Paraplegia" OR "Hereditary Spastic Paraplegias" OR "hereditary spinal ataxia" OR "Hereditary Spinal Sclerosis" OR "Hereditary Type I Motor and Sensory Neuropathy" OR "heredopathia atactica polyneuritiformis" OR "High Density Lipoprotein Deficiency" OR "HMSN IV" OR "HMSN Type V" OR "HMSN V" OR "HMSN" OR "HOKPP" OR "HSAN III" OR "HSAN Type III" OR "HSAN3" OR "HSN-III" OR "Huntington disease" OR "Huntington" OR "Huntington's" OR "Hyperkalemic Periodic Paralysis" OR "hypokalaemic periodic paralysis" OR "Hypokalemic Periodic Paralysis" OR "HYPOKPP" OR "hypomyelinating Leukodystrophy" OR "HYPOPP" OR "Inclusion Body Myopathy" OR "Inclusion Body Myositides" OR "Inclusion Body Myositis" OR "Infantile Muscular Atrophies" OR "Infantile Muscular Atrophy" OR "Inherited Polyneuropathies" OR "Inherited Polyneuropathy" OR "Inherited Spongiform Encephalopathy" OR "Insomnia Familial Fatal" OR "Isaac Syndrome" OR "Isaacs Mertens Syndrome" OR "Isaacs Syndrome" OR "Isaacs' Syndrome" OR "Isaacs-Mertens Syndrome" OR "Jansky-Bielschowsky" OR "Joseph Azorean Disease" OR "joseph disease" OR "juvenile amaurotic family idiocy" OR "juvenile amaurotic idiocy" OR "Juvenile Cerebroretinal

Degeneration" OR "juvenile chorea" OR "Juvenile Muscular Atrophies" OR "Juvenile Muscular Atrophy" OR "Juvenile Optic Atrophy" OR "Juvenile-Onset Neuroaxonal Dystrophy" OR "Kearn Syndrome" OR "Kearne-Sayre" OR "Kearns syndrome" OR "Kearns' Syndrome" OR "Kearn-Sayre" OR "Kearns-Sayer" OR "Kearns-Sayre Syndrome" OR "Kearns-Sayre" OR "Kennedy Disease" OR "Kennedy Syndrome" OR "Kennedys Disease" OR "Kennedy's Disease" OR "Kennedy's syndrome" OR "Kjer Optic Atrophy" OR "Kjers Optic Atrophy" OR "Kjer's Optic Atrophy" OR "Kjer-Type Optic Atrophy" OR "Krabbe" OR "Krabbes" OR "Krabbe's" OR "Kuf Disease" OR "Kufs Disease" OR "Kuf's Disease" OR "Kugelberg-Welander" OR "Lafora Disease" OR "Lafora" OR "Landouzy-Dejerine" OR "Lateral Scleroses" OR "Lateral Sclerosis" OR "Leber Disease" OR "Leber Hereditary Optic Atrophy" OR "Leber Hereditary optic atrophy" OR "Leber Hereditary optic neuropathy" OR "Leber optic atrophy" OR "Leber Optic Neuropathy" OR "Lebers Disease" OR "Leber's Disease" OR "Leber's Diseases" OR "Leber's Hereditary Optic Atrophy" OR "Leber's Hereditary Optic Neuropathy" OR "Leber's Optic Atrophy" OR "Lebers Optic Neuropathy" OR "Leber's Optic Neuropathy" OR "Leukodystrophy with Rosenthal Fibers" OR "Lewy body dementia" OR "Lewy body dementias" OR "Lewy Body Disease" OR "Lewy body diseases" OR "Limb-Girdle" OR "Lipofuscin Storage Disease" OR "Lobar Atrophy Of Brain" OR "Lobar Atrophy of the Brain" OR "Luft Disease" OR "Lufts Disease" OR "Luft's Disease" OR "lundborg syndrome" OR "machado disease" OR "Machado-Joseph Disease" OR "Machado-Joseph" OR "macrencephaly" OR "Megaconial Myopathies" OR "Megaconial Myopathy" OR "megalencephalic leukodystrophy" OR "megalencephaly" OR "melanodermic leukodystrophy" OR "MELAS Syndrome" OR "MELAS" OR "Menzel Type OPCA" OR "MERRF Syndrome" OR "MERRF" OR "metachromatic leucodystrophy" OR "metachromatic leucoencephalopathy" OR "Metachromatic Leukodystrophies" OR "Metachromatic Leukodystrophy" OR "metachromatic leukoencephalopathy" OR "Mitochondrial Encephalomyopathies" OR "Mitochondrial Encephalomyopathy" OR "Mitochondrial Myopathies" OR "Mitochondrial Myopathy" OR "Mitochondrial Ocular Myopathy" OR "Mitochondrial-Encephalopathy-Lactic Acidosis-Stroke" OR "Molluscum Fibrosum" OR "morbus wilson" OR "motoneuron disease" OR "Motor Neuron Disease" OR "Motor Neuron Diseases" OR "motor neurone disease" OR "motor system disease" OR "Motor System Diseases" OR "multiple mucosal neuroma" OR "multiple neurofibroma" OR "Multiple Neurofibromas" OR "multiple neuroma" OR "Multiple System Atrophies" OR "Multiple System Atrophy" OR "Multiple System Tauopathy with Presenile Dementia" OR "Multisystem Atrophies" OR "Multisystem Atrophy" OR "Multisystemic Atrophies" OR "Multisystemic Atrophy" OR "muscle dystrophia" OR "muscle dystrophy" OR "Muscle Phosphofructokinase Deficiency" OR "muscular dystrophia" OR "Muscular Dystrophies" OR "Muscular Dystrophy" OR "Myelopathic Muscular Atrophy" OR "Myoclonic Cerebellar Dyssynergia" OR "myoclonic epilepsies" OR "myoclonic epilepsy" OR "Myoclonus Epilepsies" OR "Myoclonus epilepsy" OR "myodystrophia" OR "Myodystrophies" OR "myodystrophy" OR "Myokymia" OR "Myokymias" OR "myositis calcificans" OR "Myositis Ossificans" OR "myotonia atrophica" OR "Myotonia Congenita" OR "myotonia dystrophica" OR "myotonia hereditaria" OR "Myotonia Levior" OR "myotonic atrophy" OR "myotonic dystrophia" OR "Myotonic Dystrophies" OR "Myotonic Dystrophy" OR "myotonic muscle dystrophy" OR "myotonic muscular dystrophy" OR "NBIA" OR "Nemaline Myopathies" OR "Nemaline Myopathy" OR "Nemaline Rod Disease" OR "neurodegeneration with brain iron accumulation" OR "neurofibromatoses" OR "Neurofibromatosis" OR "neurogenic amyotrophy" OR "neurogenic atrophy" OR "neurogenic muscle atrophy" OR "neurogenic muscular atrophy" OR "neurogenic osteoarthropathy" OR "neurogenic para osteoarthropathy" OR "neurogenic paraosteoarthropathy" OR "Neurogenic Scapuloperoneal Amyotrophy" OR "neuroinomatosis" OR "neuromatosis" OR "neuromyotonia" OR "Neuronal Ceroid-Lipofuscinoses" OR "Neuronal Ceroid-Lipofuscinosis" OR "neuronal ceroidosis" OR "Neuropathic Amyloidosis" OR "Nigrospinodentatal Degeneration" OR "Non fluent Primary Progressive Aphasia" OR "Non-fluent Primary Progressive Aphasia" OR "Nonfluent progressive aphasia" OR "Non-fluent progressive aphasia" OR "Normokalemic Periodic Paralysis" OR "Ocular Muscular Dystrophy" OR "oculocraniosomatic disease" OR "oculocraniosomatic syndrome" OR "Oculocraniosomatic Syndromes" OR "Oculopharyngeal" OR "olivocerebellar atrophy" OR "Olivopontocerebellar Atrophies" OR "Olivopontocerebellar Atrophy" OR "Olivopontocerebellar" OR "Olivo-Ponto-Cerebellar" OR "OPCA with Retinal Degeneration" OR "Ophthalmoplegia Plus Syndrome" OR "Ophthalmoplegia Plus Syndromes" OR "Optic Atrophy 1" OR "optic atrophy type 1" OR "ossifying fibrodysplasia" OR "Ossifying myositis" OR

"osteoplastic myopathy" OR "Pantothenate Kinase Associated Neurodegeneration" OR "Pantothenate Kinase-Associated Neurodegeneration" OR "paralysis agitans" OR "paramyotonia congenita" OR "Parkinson" OR "Parkinson's" OR "Pelizaeus Merzbacher" OR "Pelizaeus-Merzbacher Disease" OR "periodic hypokalaemic paralysis" OR "periodic hypokalemic paralysis" OR "Periodic Paralysis Hypokalemic" OR "Periodic Paralysis- Hypokalemic" OR "peroneal atrophy" OR "peroneal muscle atrophy" OR "Peroneal Muscular Atrophies" OR "Peroneal Muscular Atrophy with Pyramidal Features" OR "peroneal muscular atrophy" OR "peroneal progressive muscular atrophy" OR "Pfkm Deficiency" OR "Phytanic Acid Oxidase Deficiency" OR "phytanic acid storage disease" OR "Pick complex" OR "Pick dementia" OR "Pick Disease" OR "Pick presenile dementia" OR "Pick syndrome" OR "Pick's complex" OR "Picks Disease" OR "Pick's disease" OR "Pigmentary Pallidal Degeneration" OR "PKAN Neuroaxonal Dystrophy" OR "Pleoconial Myopathy" OR "PNFA" OR "Polyneuritic Amyloidosis" OR "PPA Syndrome" OR "PPA Syndromes" OR "Presenile Dementia" OR "Primary Adhalinopathies" OR "Primary Adhalinopathy" OR "primary dysautonomia" OR "primary dysautonomias" OR "Primary Parkinsonism" OR "Primary Progressive Nonfluent Aphasia" OR "Primary Senile Degenerative Dementia" OR "pringle bourneville disease" OR "pringle disease" OR "pringle syndrome" OR "Progeria-Like Syndrome" OR "Progeria-Like Syndromes" OR "progressive external ophthalmoplegia" OR "progressive lenticular degeneration" OR "Progressive Muscular Atrophies" OR "Progressive Muscular Atrophy" OR "Progressive nonfluent aphasia" OR "Progressive non-fluent aphasia" OR "PROMM" OR "Proximal Myotonic Myopathies" OR "proximal myotonic myopathy" OR "Pseudohypertrophic Muscular Dystrophy" OR "Pseudomyotonia" OR "Pseudosclerosis" OR "Psychosine Lipidosis" OR "Quantal Squander" OR "Ramsay Hunt " OR "Recklinghausen disease" OR "Recklinghausens Disease" OR "Recklinghausen's Disease" OR "Refsum Disease" OR "Refsum" OR "Refsum’s" OR "Refsums" OR "Ricker" OR "Riley-Day Syndrome" OR "Rod Myopathies" OR "Rod Myopathy" OR "Rod-Body Myopathy" OR "Roussy Levy" OR "Santavuori-Haltia" OR "Sarcoglycanopathies" OR "Sarcoglycanopathy" OR "SBMA" OR "SCA1" OR "SCA1s" OR "SCA2" OR "SCA3" OR "SCA4" OR "SCA5" OR "SCA6" OR "SCA7" OR "Scapulo-ilio-peroneal" OR "Scapuloperoneal" OR "Sclerosis Tuberosa" OR "sebaceous adenoma" OR "Semantic Dementia" OR "Semantic Dementias" OR "Senile Dementia" OR "Shy Drager syndrome" OR "Spastic Paraplegia 2" OR "Spastic Paraplegia Type 2" OR "Spielmeyer-Sjogren" OR "Spielmeyer-Vogt" OR "Spinal Amyotrophies" OR "Spinal Amyotrophy" OR "spinal and bulbar muscular atrophy" OR "spinal heredoataxia" OR "spinal muscle atrophy" OR "Spinal Muscular Atrophy" OR "spine muscle atrophy" OR "spinobulbar muscular atrophy" OR "Spinocerebellar Ataxia" OR "Spinocerebellar Ataxias" OR "Spinocerebellar Atrophies" OR "spinocerebellar atrophy" OR "Spinocerebellar Degeneration" OR "spinocerebellar degenerations" OR "Spongiform Leukodystrophy" OR "Spongy Degeneration" OR "Spongy Disease " OR "Steinert" OR "Steinerts" OR "Steinert's" OR "Struempell Lorrain" OR "Struempell" OR "Strumpel" OR "Strumpell Lorrain" OR "Strumpell" OR "Strumpell's" OR "Strumpel's" OR "sulfatide lipidosis" OR "sulfatidosis" OR "sulphatide lipidosis" OR "Syndrome of Continuous Muscle Activity" OR "Tangier Disease" OR "tarui disease" OR "Taruis Disease" OR "Tarui's Disease" OR "TDP 43 pathology" OR "TDP 43 proteinopathies" OR "TDP-43 Proteinopathy" OR "Thomsen Disease" OR "Thomsen myotonia" OR "Thomsens Disease" OR "Thomsen's Disease" OR "Thomsens myotonia" OR "Thomsen's myotonia" OR "Tibial Muscular Dystrophy" OR "Torsion dystonia" OR "Torsion Dystonias" OR "torsion spasm" OR "Tourette Syndrome" OR "Tourette" OR "Tourette’s" OR "Tourettes" OR "tuberose sclerosis" OR "Tuberous Sclerosis" OR "Udd Myopathy" OR "Unverricht Disease" OR "Unverricht Lundborg Syndrome" OR "Unverricht-Lundborg Disease" OR "Unverricht-Lundborg Syndrome" OR "unverricht-lundborg" OR "Van Bogaert-Bertrand Syndrome" OR "Vogt-Spielmeyer Disease" OR "Wadia Swami Syndrome" OR "Wadia-Swami Syndrome" OR "Watson Syndrome" OR "Werdnig Hoffman" OR "Werdnig-Hoffmann" OR "Westphall Disease" OR "Westphal-Strumpell" OR "Wilhelmsen-Lynch Disease" OR "Wilson disease" OR "Wilson diseases" OR "wilson syndrome" OR "Wilsons Disease" OR "Wilson's disease" OR "Wolfram Syndrome" OR "X ALD" OR "ziehen oppenheim" OR (Divry AND bogaert) OR (Motor AND "Vocal Tic") OR (Myoencephalopathy AND "lactic acidosis" AND stroke) OR (Schilder AND Addison) OR (Siemerling AND Creutzfeldt) OR "Transthyretin amyloidosis" OR "TTR amyloidosis").ti,ab,kw.)

Search component #3: Animal population [1]

exp animal experiment/ or exp animal model/ or exp experimental animal/ or exp transgenic animal/ or exp male animal/ or exp female animal/ or exp juvenile animal/ OR animal/ OR chordata/ OR vertebrate/ OR tetrapod/ OR exp fish/ OR amniote/ OR exp amphibia/ OR mammal/ OR exp reptile/ OR exp sauropsid/ OR therian/OR exp monotremate/ OR placental mammals/ OR exp marsupial/ OR Euarchontoglires/ OR exp Afrotheria/ OR exp Boreoeutheria/ OR exp Laurasiatheria/ OR exp Xenarthra/ OR primate/ OR exp Dermoptera/ OR exp Glires/ OR exp Scandentia/ OR Haplorhini/ OR exp prosimian/ OR simian/ OR exp tarsiiform/ OR Catarrhini/ OR exp Platyrrhini/ OR ape/ OR exp Cercopithecidae/ OR hominid/ OR exp hylobatidae/ OR exp chimpanzee/ OR exp gorilla/ OR exp orang utan/ OR (animal OR animals OR pisces OR fish OR fishes OR catfish OR catfishes OR sheatfish OR silurus OR arius OR heteropneustes OR clarias OR gariepinus OR fathead minnow OR fathead minnows OR pimephales OR promelas OR cichlidae OR trout OR trouts OR char OR chars OR salvelinus OR salmo OR oncorhynchus OR guppy OR guppies OR millionfish OR poecilia OR goldfish OR goldfishes OR carassius OR auratus OR mullet OR mullets OR mugil OR curema OR shark OR sharks OR cod OR cods OR gadus OR morhua OR carp OR carps OR cyprinus OR carpio OR killifish OR eel OR eels OR anguilla OR zander OR sander OR lucioperca OR stizostedion OR turbot OR turbots OR psetta OR flatfish OR flatfishes OR plaice OR pleuronectes OR platessa OR tilapia OR tilapias OR oreochromis OR sarotherodon OR common sole OR dover sole OR solea OR zebrafish OR zebrafishes OR danio OR rerio OR seabass OR dicentrarchus OR labrax OR morone OR lamprey OR lampreys OR petromyzon OR pumpkinseed OR pumpkinseeds OR lepomis OR gibbosus OR herring OR clupea OR harengus OR amphibia OR amphibian OR amphibians OR anura OR salientia OR frog OR frogs OR rana OR toad OR toads OR bufo OR xenopus OR laevis OR bombina OR epidalea OR calamita OR salamander OR salamanders OR newt OR newts OR triturus OR reptilia OR reptile OR reptiles OR bearded dragon OR pogona OR vitticeps OR iguana OR iguanas OR lizard OR lizards OR anguis fragilis OR turtle OR turtles OR snakes OR snake OR aves OR bird OR birds OR quail OR quails OR coturnix OR bobwhite OR colinus OR virginianus OR poultry OR poultries OR fowl OR fowls OR chicken OR chickens OR gallus OR zebra finch OR taeniopygia OR guttata OR canary OR canaries OR serinus OR canaria OR parakeet OR parakeets OR grasskeet OR parrot OR parrots OR psittacine OR psittacines OR shelduck OR tadorna OR goose OR geese OR branta OR leucopsis OR woodlark OR lullula OR flycatcher OR ficedula OR hypoleuca OR dove OR doves OR geopelia OR cuneata OR duck OR ducks OR greylag OR graylag OR anser OR harrier OR circus pygargus OR red knot OR great knot OR calidris OR canutus OR godwit OR limosa OR lapponica OR meleagris OR gallopavo OR jackdaw OR corvus OR monedula OR ruff OR philomachus OR pugnax OR lapwing OR peewit OR plover OR vanellus OR swan OR cygnus OR columbianus OR bewickii OR gull OR chroicocephalus OR ridibundus OR albifrons OR great tit OR parus OR aythya OR fuligula OR streptopelia OR risoria OR spoonbill OR platalea OR leucorodia OR blackbird OR turdus OR merula OR blue tit OR cyanistes OR pigeon OR pigeons OR columba OR pintail OR anas OR starling OR sturnus OR owl OR athene noctua OR pochard OR ferina OR cockatiel OR nymphicus OR hollandicus OR skylark OR alauda OR tern OR sterna OR teal OR crecca OR oystercatcher OR haematopus OR ostralegus OR shrew OR shrews OR sorex OR araneus OR crocidura OR russula OR european mole OR talpa OR chiroptera OR bat OR bats OR eptesicus OR serotinus OR myotis OR dasycneme OR daubentonii OR pipistrelle OR pipistrellus OR cat OR cats OR felis OR catus OR feline OR dog OR dogs OR canis OR canine OR canines OR otter OR otters OR lutra OR badger OR badgers OR meles OR fitchew OR fitch OR foumart or foulmart OR ferrets OR ferret OR polecat OR polecats OR mustela OR putorius OR weasel OR weasels OR fox OR foxes OR vulpes OR common seal OR phoca OR vitulina OR grey seal OR halichoerus OR horse OR horses OR equus OR equine OR equidae OR donkey OR donkeys OR mule OR mules OR pig OR pigs OR swine OR swines OR hog OR hogs OR boar OR boars OR porcine OR piglet OR piglets OR sus OR scrofa OR llama OR llamas OR lama OR glama OR deer OR deers OR cervus OR elaphus OR cow OR cows OR bos taurus OR bos indicus OR bovine OR bull OR bulls OR cattle OR bison OR bisons OR sheep OR sheeps OR ovis aries OR ovine

OR lamb OR lambs OR mouflon OR mouflons OR goat OR goats OR capra OR caprine OR chamois OR rupicapra OR leporidae OR lagomorpha OR lagomorph OR rabbit OR rabbits OR oryctolagus OR cuniculus OR laprine OR hares OR lepus OR rodentia OR rodent OR rodents OR murinae OR mouse OR mice OR mus OR musculus OR murine OR woodmouse OR apodemus OR rat OR rats OR rattus OR norvegicus OR guinea pig OR guinea pigs OR cavia OR porcellus OR hamster OR hamsters OR mesocricetus OR cricetulus OR cricetus OR gerbil OR gerbils OR jird OR jirds OR meriones OR unguiculatus OR jerboa OR jerboas OR jaculus OR chinchilla OR chinchillas OR beaver OR beavers OR castor fiber OR castor canadensis OR sciuridae OR squirrel OR squirrels OR sciurus OR chipmunk OR chipmunks OR marmot OR marmots OR marmota OR suslik OR susliks OR spermophilus OR cynomys OR cottonrat OR cottonrats OR sigmodon OR vole OR voles OR microtus OR myodes OR glareolus OR primate OR primates OR prosimian OR prosimians OR lemur OR lemurs OR lemuridae OR loris OR bush baby OR bush babies OR bushbaby OR bushbabies OR galago OR galagos OR anthropoidea OR anthropoids OR simian OR simians OR monkey OR monkeys OR marmoset OR marmosets OR callithrix OR cebuella OR tamarin OR tamarins OR saguinus OR leontopithecus OR squirrel monkey OR squirrel monkeys OR saimiri OR night monkey OR night monkeys OR owl monkey OR owl monkeys OR douroucoulis OR aotus OR spider monkey OR spider monkeys OR ateles OR baboon OR baboons OR papio OR rhesus monkey OR macaque OR macaca OR mulatta OR cynomolgus OR fascicularis OR green monkey OR green monkeys OR chlorocebus OR vervet OR vervets OR pygerythrus OR hominoidea OR ape OR apes OR hylobatidae OR gibbon OR gibbons OR siamang OR siamangs OR nomascus OR symphalangus OR hominidae OR orangutan OR orangutans OR pongo OR chimpanzee OR chimpanzees OR pan troglodytes OR bonobo OR bonobos OR pan paniscus OR gorilla OR gorillas OR troglodytes).ti,ab.

Embase Search Strategy: ((#1 AND #2) AND #3))

2. PubMed

Search component #1': Intervention

((Biological transport [mesh] OR cell-penetrating peptides [mesh] OR drug carriers [mesh] OR drug delivery systems [mesh] OR liposomes [mesh] OR nanoparticles [mesh] OR peptides [mesh:noexp]) OR (AAV [tiab] OR Adeno-associated [tiab] OR Cationic lipid [tiab] OR Cationic lipids [tiab] OR "Cell-penetrating peptide" [tiab] OR "Cell-penetrating peptides" [tiab] OR Chitosan [tiab] OR Conjugate [tiab] OR Conjugated [tiab] OR Conjugates [tiab] OR Conjugation [tiab] OR Copolymer[tiab] OR Co-polymer[tiab] OR Co-polymers[tiab] OR Copolymers[tiab] OR CPP [tiab] OR CPPs [tiab] OR Dendrimer [tiab] OR Dendrimers [tiab] OR Dendriplex [tiab] OR Dendriplexes [tiab] OR Excipient [tiab] OR Excipients [tiab] OR Formulation [tiab] OR Formulations [tiab] OR Lipofectamine [tiab] OR Lipofection [tiab] OR Lipoplex [tiab] OR Lipoplexes [tiab] OR liposome [tiab] OR liposomes [tiab] OR Nanoballs [tiab] OR Nanocapsule [tiab] OR Nanocapsules [tiab] OR Nanocarrier [tiab] OR Nanocarriers [tiab] OR Nanocomposite [tiab] OR Nanocomposites [tiab] OR Nanoconjugate [tiab] OR Nanoconjugates [tiab] OR Nanocrystal [tiab] OR Nanocrystals [tiab] OR nanoparticle [tiab] OR nanoparticles [tiab] OR nanoparticulate [tiab] OR Nanorod [tiab] OR Nanorods [tiab] OR Nanotube [tiab] OR Nanotubes [tiab] OR Nano-balls [tiab] OR Nano-capsule [tiab] OR Nano-capsules [tiab] OR Nano-carrier [tiab] OR Nano-carriers [tiab] OR Nano-composite [tiab] OR Nano-composites [tiab] OR Nano-conjugate [tiab] OR Nano-conjugates [tiab] OR Nano-crystal [tiab] OR Nano-crystals [tiab] OR Nano-particle [tiab] OR Nano-particles [tiab] OR nano-particulate [tiab] OR Nano-rod [tiab] OR Nano-rods [tiab] OR Nano-tube [tiab] OR Nano-tubes [tiab] OR Oligoplex [tiab] OR Oligoplexes [tiab] OR PEI [tiab] OR peptide-linked [tiab] OR PLGA [tiab] OR Polyelectrolyte [tiab] OR Polyelectrolytes [tiab] OR Polyethylenimine [tiab] OR polylactic-co-glycolic acid [tiab] OR Polymersomes [tiab] OR Polyplex [tiab] OR Polyplexes [tiab] OR Protamine [tiab] OR Silica [tiab] OR Thiomer [tiab] OR Thiomers [tiab] OR Vector [tiab] OR Vectorized [tiab] OR Vectors [tiab] OR Viral [tiab] OR Virosome [tiab] OR Virosomes [tiab] OR Virus [tiab] OR Viruses [tiab] OR Virus-like particle [tiab] OR Virus-like particles [tiab] OR VLP [tiab] OR VLPs [tiab])) AND ((Morpholinos [mesh] OR oligonucleotides [mesh:noexp] OR Oligonucleotides, Antisense [mesh] OR Oligoribonucleotides [mesh] OR Peptide Nucleic Acids [mesh] OR Phosphorothioate Oligonucleotides [mesh]) OR (antisense [tiab] OR AON [tiab] OR AONs [tiab] OR ASO [tiab] OR ASOs [tiab] OR LNA [tiab] OR LNAs [tiab] OR Locked nucleic acid [tiab] OR Locked nucleic acids [tiab] OR MORF [tiab] OR MORFs [tiab] OR Morpholino [tiab] OR Morpholinos [tiab] OR Peptide nucleic acid [tiab] OR Peptide nucleic acids [tiab] OR Phosphorothioate [tiab] OR Phosphorothioates [tiab] OR PMO [tiab] OR PMOs [tiab] OR PNA [tiab] OR PNAs [tiab] OR SCO [tiab] OR SCOs [tiab] OR splice-correcting oligonucleotide [tiab] OR splice-correcting oligonucleotides [tiab] OR splice-switching oligonucleotide [tiab] OR splice-switching oligonucleotides [tiab] OR SSO [tiab] OR SSOs [tiab]))

Search component #2': Disease

((((degenerative [tiab] AND nervous system [tiab]) OR (degenerative [tiab] AND neurologic [tiab]) OR (degenerative [tiab] AND neurological [tiab]) OR (degenerative [tiab] AND spinal cord [tiab]) OR fasciculation [tiab] OR neurodegenerative [tiab] OR neuromuscular [tiab]) AND (autosomal [tiab] OR congenital [tiab] OR dominant [tiab] OR familial [tiab] OR genealogical [tiab] OR genetic [tiab] OR hereditary [tiab] OR heritable [tiab] OR inborn [tiab] OR inheritable [tiab] OR inheritance [tiab] OR inherited [tiab] OR recessive [tiab] OR single-gene [tiab] OR X-linked [tiab]) OR ((Siemerling[tiab] AND Creutzfeldt[tiab]) OR "Adenoma Sebaceum"[tiab] OR "adreno leucodystrophy"[tiab] OR "adreno leukodystrophy"[tiab] OR "adrenoleucodystrophy"[tiab] OR "Adrenoleukodystrophy"[tiab] OR "adrenoleukomyeloneuropathy"[tiab] OR "adrenomyeloneuropathy"[tiab] OR "Alexander Disease"[tiab] OR "alexander syndrome"[tiab] OR "Alexanders Disease"[tiab] OR "Alexander's Disease"[tiab] OR "Alstrom Hallgren Syndrome"[tiab] OR "Alstrom Syndrome"[tiab] OR "Alström Syndrome"[tiab] OR "Alstrom-Hallgren Syndrome"[tiab] OR "Alstrom's Syndrome"[tiab] OR "Alzeimer's"[tiab] OR "Alzheimer"[tiab] OR "Alzheimers"[tiab] OR "amaurotic familial idiocy"[tiab] OR "amaurotic idiocy"[tiab] OR

"amyloid neuropathies"[tiab] OR "Amyloid Neuropathy"[tiab] OR "Amyloid Polyneuropathies"[tiab] OR "amyloid polyneuropathy"[tiab] OR "amyloidotic neuropathy"[tiab] OR "amyloidotic polyneuropathy"[tiab] OR "Amyotrophic Lateral Sclerosis"[tiab] OR "analgesia congenita"[tiab] OR "Analphalipoproteinemia"[tiab] OR "Anterior Horn Cell Disease"[tiab] OR "ARSA Deficiency"[tiab] OR "Arylsulfatase A Deficiencies"[tiab] OR "Arylsulfatase A Deficiency"[tiab] OR "ASP Deficiency"[tiab] OR "ASPA Deficiency"[tiab] OR "Aspartoacylase Deficiency"[tiab] OR "Ataxia Telangiectasia Syndrome"[tiab] OR "Ataxia Telangiectasia"[tiab] OR "ataxia teleangiectasia"[tiab] OR "Autosomal Dominant Optic Atrophy"[tiab] OR "Autosomal Dominant Spastic Paraplegia"[tiab] OR "Autosomal Dominant Striatonigral Degeneration"[tiab] OR "Autosomal Recessive Spastic Paraplegia"[tiab] OR "Azorean Disease"[tiab] OR "balo disease"[tiab] OR "Baltic Myoclonus"[tiab] OR "bar syndrome"[tiab] OR "Batten"[tiab] OR "Becker Disease"[tiab] OR "Becker Muscular Dystrophy"[tiab] OR "Becker's Muscular Dystrophy"[tiab] OR "beta-Galactosidase Deficiencies"[tiab] OR "beta-Galactosidase Deficiency"[tiab] OR "Bilateral Acoustic Neurinoma"[tiab] OR "Bilateral Acoustic Neurinomas"[tiab] OR "Bilateral Acoustic Neuroma"[tiab] OR "Bilateral Acoustic Neuromas"[tiab] OR "Bilateral Acoustic Schwannoma"[tiab] OR "Bilateral Acoustic Schwannomas"[tiab] OR "bourneville disease"[tiab] OR "Bourneville Phakomatosis"[tiab] OR "bourneville pringle syndrome"[tiab] OR "Bourneville Pringle's Disease"[tiab] OR "bourneville syndrome"[tiab] OR "bourneville tuberous sclerosis"[tiab] OR "Bourneville-Pringle Disease"[tiab] OR "Bourneville-Pringle's Disease"[tiab] OR "Bourneville's Disease"[tiab] OR "Bourneville's Syndrome"[tiab] OR "brain sclerosis"[tiab] OR "bulbar dysfunction"[tiab] OR "bulbar muscle weakness"[tiab] OR "Bulbar palsies"[tiab] OR "Bulbar Palsy"[tiab] OR "bulbar paralysis"[tiab] OR "bulbar sign"[tiab] OR "bulbar symptom"[tiab] OR "bulbar syndrome"[tiab] OR "bulbar weakness"[tiab] OR "Bulbospinal atrophy"[tiab] OR "bulbospinal muscular atrophy"[tiab] OR "bulbo-spinal muscular atrophy"[tiab] OR "Bulbospinal Neuronopathy"[tiab] OR "Canavan Disease"[tiab] OR "canavan syndrome"[tiab] OR "Canavan's disease"[tiab] OR "Canavan-van Bogaert-Bertrand Disease"[tiab] OR "Central core disease"[tiab] OR "Central Core Diseases"[tiab] OR "Central Core Myopathies"[tiab] OR "Central Core Myopathy"[tiab] OR "Cerebellar Degeneration with Slow Eye Movements"[tiab] OR "cerebellar dyssynergia"[tiab] OR "Cerebral Scleroses"[tiab] OR "cerebral sclerosis"[tiab] OR "Cerebroside Sulphatase Deficiency"[tiab] OR "Ceroid Storage Disease"[tiab] OR "Ceroid Storage Diseases"[tiab] OR "Channelopathy-Associated Insensitivity To Pain"[tiab] OR "Charcot Disease"[tiab] OR "charcot marie disease"[tiab] OR "Charcot Marie Tooth"[tiab] OR "Cholesterol Thesaurismosis"[tiab] OR "chronic progressive chorea"[tiab] OR "Chronic Progressive External Ophthalmoplegia"[tiab] OR "Chronic Progressive Hereditary Chorea"[tiab] OR "Circumscribed Lobar Atrophy of the Brain"[tiab] OR "Cockayne Syndrome"[tiab] OR "Cockayne"[tiab] OR "Cockayne's"[tiab] OR "concentric sclerosis"[tiab] OR "congenital analgesia"[tiab] OR "congenital atonic sclerotic muscular dystrophy"[tiab] OR "congenital generalized analgesia"[tiab] OR "congenital indifference to pain"[tiab] OR "congenital insensitivity to pain"[tiab] OR "Congenital Myopathy"[tiab] OR "Congenital Myotonia"[tiab] OR "Congenital Pain Insensitivity"[tiab] OR "congenital paramyotonia"[tiab] OR "Continuous Muscle Activity Syndrome"[tiab] OR "Copper Storage Disease"[tiab] OR "CPEO with Ragged Red Fibers"[tiab] OR "cranial nerve paralysis"[tiab] OR "DDPAC"[tiab] OR "dejerine sottas"[tiab] OR "Dejerine-Thomas"[tiab] OR "dementia with Lewy bodies"[tiab] OR "dementia with Lewy body"[tiab] OR "Dementia with Lobar Atrophy and Neuronal Cytoplasmic Inclusions"[tiab] OR "DIDMOAD"[tiab] OR "Dilated Cardiomyopathy"[tiab] OR "Disinhibition-Dementia-Parkinsonism-Amyotrophy"[tiab] OR "Distal 1 Myopathy"[tiab] OR "Distal Muscular Dystrophies"[tiab] OR "Distal Muscular Dystrophy"[tiab] OR "Distal Myopathies"[tiab] OR "Distal Myopathy"[tiab] OR "Dominant Hereditary Sensory Neuropathy"[tiab] OR "Dominant Optic Atrophies"[tiab] OR "dominant optic atrophy"[tiab] OR "Duchenne"[tiab] OR "dysantonomia"[tiab] OR "Dysmyelinogenic Leukodystrophy"[tiab] OR "Dystonia Musculorum Deformans"[tiab] OR "dystrophia myotonica"[tiab] OR "dystrophic myotonia"[tiab] OR "Emery-Dreifuss"[tiab] OR "encephalitis periaxialis"[tiab] OR "epiloia"[tiab] OR "Eulenburg"[tiab] OR "Facioscapulohumeral"[tiab] OR "Facio-Scapulo-Humeral"[tiab] OR "Facioscapuloperoneal"[tiab] OR "familial autonomic dysfunction"[tiab] OR "Familial dysautonomia"[tiab] OR "familial dysautonomy"[tiab] OR "Familial Fatal Insomnia"[tiab] OR "Familial Periodic Paralyses"[tiab] OR "Familial Periodic Paralysis"[tiab] OR "Familial Polyneuropathies"[tiab] OR "Familial Polyneuropathy"[tiab] OR "familial spastic paralysis"[tiab] OR "familial spastic paraparesis"[tiab] OR "familial spastic paraplegia"[tiab] OR

"Fatal Familial Insomnia"[tiab] OR "Fazio-Londe Disease"[tiab] OR "Fazio-Londe Syndrome"[tiab] OR "Fibrinoid Degeneration of Astrocytes"[tiab] OR "Fibrodysplasia Ossificans"[tiab] OR "Friedreich Ataxia"[tiab] OR "Friedreich"[tiab] OR "Friedreich's"[tiab] OR "Friedreichs"[tiab] OR "frontal dementia"[tiab] OR "frontal lobe dementia"[tiab] OR "Frontotemporal Dementia"[tiab] OR "frontotemporal dementias"[tiab] OR "Frontotemporal Lobar Degeneration"[tiab] OR "Frontotemporal Lobe Dementia"[tiab] OR "FTD"[tiab] OR "FTDP"[tiab] OR "FTLD"[tiab] OR "Fukuhara disease"[tiab] OR "Galactocerebrosidase Deficiency"[tiab] OR "Galactosylceramidase Deficiency"[tiab] OR "galactosylceramide lipidosis"[tiab] OR "GALC Deficiency"[tiab] OR "Gehrig Disease"[tiab] OR "Gehrig's Disease"[tiab] OR "Generalized Myotonia"[tiab] OR "Generalized Myotonias"[tiab] OR "Gerstmann-Straussler"[tiab] OR "Gerstmann-Straussler-Scheinker Disease"[tiab] OR "Giant Axonal Neuropathy"[tiab] OR "globoid cell leucodystrophy"[tiab] OR "Globoid Cell Leukodystrophy"[tiab] OR "Globoid Leukodystrophies"[tiab] OR "Globoid Leukodystrophy"[tiab] OR "Glycogen Storage Disease Type VII"[tiab] OR "Glycogen Storage Disease VII"[tiab] OR "Graefe's Disease"[tiab] OR "Greenfield Disease"[tiab] OR "Greenfield's Disease"[tiab] OR "GSD VII"[tiab] OR "GSS"[tiab] OR "Guam Disease"[tiab] OR "Hallervorden Spatz"[tiab] OR "Hallervorden-Spatz"[tiab] OR "Hauptmann-Thannhauser"[tiab] OR "HDDD1"[tiab] OR "HDDD2"[tiab] OR "hemimegalencephaly"[tiab] OR "hepatocerebral degeneration"[tiab] OR "Hepatolenticular Degeneration"[tiab] OR "hepatolenticular syndrome"[tiab] OR "Hereditary Areflexic Dystasia"[tiab] OR "hereditary chorea"[tiab] OR "Hereditary Dysphasic Disinhibition Dementia"[tiab] OR "hereditary motor and sensory neuropathies"[tiab] OR "Hereditary Motor and Sensory Neuropathy "[tiab] OR "Hereditary Motor And Sensory Neuropathy V"[tiab] OR "Hereditary Motor Neuronopathies"[tiab] OR "Hereditary Motor Neuronopathy"[tiab] OR "hereditary motor sensory neuropathy"[tiab] OR "hereditary myotonia"[tiab] OR "Hereditary Optic Atrophies"[tiab] OR "Hereditary Optic Atrophy"[tiab] OR "hereditary optic nerve atrophy"[tiab] OR "Hereditary Optic Neuroretinopathy"[tiab] OR "hereditary sensorimotor neuropathy"[tiab] OR "Hereditary Sensory and Autonomic Neuropathy"[tiab] OR "hereditary sensory and motor neuropathy"[tiab] OR "Hereditary Sensory Neuropathy"[tiab] OR "hereditary spastic paralysis"[tiab] OR "hereditary spastic paraparesis"[tiab] OR "Hereditary Spastic Paraplegia"[tiab] OR "Hereditary Spastic Paraplegias"[tiab] OR "hereditary spinal ataxia"[tiab] OR "Hereditary Spinal Sclerosis"[tiab] OR "heredopathia atactica polyneuritiformis"[tiab] OR "High Density Lipoprotein Deficiency"[tiab] OR "HMSN Type V"[tiab] OR "HMSN V"[tiab] OR "HMSN"[tiab] OR "HOKPP"[tiab] OR "HSAN III"[tiab] OR "HSAN Type III"[tiab] OR "HSAN3"[tiab] OR "HSN-III"[tiab] OR "Huntington disease"[tiab] OR "Huntington"[tiab] OR "Huntington's"[tiab] OR "Hyperkalemic Periodic Paralysis"[tiab] OR "hypokalaemic periodic paralysis"[tiab] OR "Hypokalemic Periodic Paralysis"[tiab] OR "HYPOKPP"[tiab] OR "hypomyelinating Leukodystrophy"[tiab] OR "HYPOPP"[tiab] OR "Inclusion Body Myopathy"[tiab] OR "Inclusion Body Myositides"[tiab] OR "Inclusion Body Myositis"[tiab] OR "Infantile Muscular Atrophies"[tiab] OR "Infantile Muscular Atrophy"[tiab] OR "Inherited Polyneuropathies"[tiab] OR "Inherited Polyneuropathy"[tiab] OR "Isaac Syndrome"[tiab] OR "Isaacs Mertens Syndrome"[tiab] OR "Isaacs Syndrome"[tiab] OR "Isaacs' Syndrome"[tiab] OR "Isaacs-Mertens Syndrome"[tiab] OR "Jansky-Bielschowsky"[tiab] OR "Joseph Azorean Disease"[tiab] OR "joseph disease"[tiab] OR "juvenile amaurotic family idiocy"[tiab] OR "juvenile amaurotic idiocy"[tiab] OR "Juvenile Cerebroretinal Degeneration"[tiab] OR "Juvenile Muscular Atrophies"[tiab] OR "Juvenile Muscular Atrophy"[tiab] OR "Juvenile Optic Atrophy"[tiab] OR "Juvenile-Onset Neuroaxonal Dystrophy"[tiab] OR "Kearn Syndrome"[tiab] OR "Kearne-Sayre"[tiab] OR "Kearns syndrome"[tiab] OR "Kearns' Syndrome"[tiab] OR "Kearn-Sayre"[tiab] OR "Kearns-Sayer"[tiab] OR "Kearns-Sayre Syndrome"[tiab] OR "Kearns-Sayre"[tiab] OR "Kennedy Disease"[tiab] OR "Kennedy Syndrome"[tiab] OR "Kennedys Disease"[tiab] OR "Kennedy's Disease"[tiab] OR "Kennedy's syndrome"[tiab] OR "Kjer Optic Atrophy"[tiab] OR "Kjer's Optic Atrophy"[tiab] OR "Kjer-Type Optic Atrophy"[tiab] OR "Krabbe"[tiab] OR "Krabbes"[tiab] OR "Krabbe's"[tiab] OR "Kuf Disease"[tiab] OR "Kufs Disease"[tiab] OR "Kuf's Disease"[tiab] OR "Kugelberg-Welander"[tiab] OR "Lafora Disease"[tiab] OR "Lafora"[tiab] OR "Landouzy-Dejerine"[tiab] OR "Lateral Scleroses"[tiab] OR "Lateral Sclerosis"[tiab] OR "Leber Disease"[tiab] OR "Leber Hereditary Optic Atrophy"[tiab] OR "Leber Hereditary optic neuropathy"[tiab] OR "Leber optic atrophy"[tiab] OR "Leber Optic Neuropathy"[tiab] OR "Leber's Disease"[tiab] OR "Leber's Diseases"[tiab] OR "Leber's Hereditary Optic Atrophy"[tiab] OR "Leber's Hereditary Optic Neuropathy"[tiab] OR "Leber's Optic Atrophy"[tiab] OR "Leber's Optic Neuropathy"[tiab]

OR "Leukodystrophy with Rosenthal Fibers"[tiab] OR "Lewy body dementia"[tiab] OR "Lewy body dementias"[tiab] OR "Lewy Body Disease"[tiab] OR "Lewy body diseases"[tiab] OR "Limb-Girdle"[tiab] OR "Lipofuscin Storage Disease"[tiab] OR "Lobar Atrophy Of Brain"[tiab] OR "Lobar Atrophy of the Brain"[tiab] OR "Luft Disease"[tiab] OR "Luft's Disease"[tiab] OR "lundborg syndrome"[tiab] OR "machado disease"[tiab] OR "Machado-Joseph Disease"[tiab] OR "Machado-Joseph"[tiab] OR "macrencephaly"[tiab] OR "Megaconial Myopathy"[tiab] OR "megalencephalic leukodystrophy"[tiab] OR "megalencephaly"[tiab] OR "melanodermic leukodystrophy"[tiab] OR "MELAS Syndrome"[tiab] OR "MELAS"[tiab] OR "Menzel Type OPCA"[tiab] OR "MERRF Syndrome"[tiab] OR "MERRF"[tiab] OR "metachromatic leucodystrophy"[tiab] OR "metachromatic leucoencephalopathy"[tiab] OR "Metachromatic Leukodystrophies"[tiab] OR "Metachromatic Leukodystrophy"[tiab] OR "metachromatic leukoencephalopathy"[tiab] OR "Mitochondrial Encephalomyopathies"[tiab] OR "Mitochondrial Encephalomyopathy"[tiab] OR "Mitochondrial Myopathies"[tiab] OR "Mitochondrial Myopathy"[tiab] OR "Mitochondrial Ocular Myopathy"[tiab] OR "Mitochondrial-Encephalopathy-Lactic Acidosis-Stroke"[tiab] OR "Molluscum Fibrosum"[tiab] OR "morbus wilson"[tiab] OR "motoneuron disease"[tiab] OR "Motor Neuron Disease"[tiab] OR "Motor Neuron Diseases"[tiab] OR "motor neurone disease"[tiab] OR "motor system disease"[tiab] OR "Motor System Diseases"[tiab] OR "multiple mucosal neuroma"[tiab] OR "multiple neurofibroma"[tiab] OR "Multiple Neurofibromas"[tiab] OR "multiple neuroma"[tiab] OR "Multiple System Atrophies"[tiab] OR "Multiple System Atrophy"[tiab] OR "Multiple System Tauopathy with Presenile Dementia"[tiab] OR "Multisystem Atrophies"[tiab] OR "Multisystem Atrophy"[tiab] OR "Multisystemic Atrophies"[tiab] OR "Multisystemic Atrophy"[tiab] OR "muscle dystrophy"[tiab] OR "Muscle Phosphofructokinase Deficiency"[tiab] OR "muscular dystrophia"[tiab] OR "Muscular Dystrophies"[tiab] OR "Muscular Dystrophy"[tiab] OR "Myelopathic Muscular Atrophy"[tiab] OR "Myoclonic Cerebellar Dyssynergia"[tiab] OR "myoclonic epilepsies"[tiab] OR "myoclonic epilepsy"[tiab] OR "Myoclonus Epilepsies"[tiab] OR "Myoclonus epilepsy"[tiab] OR "myodystrophia"[tiab] OR "Myodystrophies"[tiab] OR "myodystrophy"[tiab] OR "Myokymia"[tiab] OR "Myokymias"[tiab] OR "Myositis Ossificans"[tiab] OR "myotonia atrophica"[tiab] OR "Myotonia Congenita"[tiab] OR "myotonia dystrophica"[tiab] OR "myotonia hereditaria"[tiab] OR "Myotonia Levior"[tiab] OR "myotonic atrophy"[tiab] OR "myotonic dystrophia"[tiab] OR "Myotonic Dystrophies"[tiab] OR "Myotonic Dystrophy"[tiab] OR "myotonic muscular dystrophy"[tiab] OR "NBIA"[tiab] OR "Nemaline Myopathies"[tiab] OR "Nemaline Myopathy"[tiab] OR "Nemaline Rod Disease"[tiab] OR "neurodegeneration with brain iron accumulation"[tiab] OR "neurofibromatoses"[tiab] OR "Neurofibromatosis"[tiab] OR "neurogenic amyotrophy"[tiab] OR "neurogenic atrophy"[tiab] OR "neurogenic muscle atrophy"[tiab] OR "neurogenic muscular atrophy"[tiab] OR "neurogenic osteoarthropathy"[tiab] OR "neurogenic paraosteoarthropathy"[tiab] OR "neuroinomatosis"[tiab] OR "neuromatosis"[tiab] OR "neuromyotonia"[tiab] OR "Neuronal Ceroid-Lipofuscinoses"[tiab] OR "Neuronal Ceroid-Lipofuscinosis"[tiab] OR "neuronal ceroidosis"[tiab] OR "Neuropathic Amyloidosis"[tiab] OR "Nigrospinodentatal Degeneration"[tiab] OR "Non fluent Primary Progressive Aphasia"[tiab] OR "Non-fluent Primary Progressive Aphasia"[tiab] OR "Nonfluent progressive aphasia"[tiab] OR "Non-fluent progressive aphasia"[tiab] OR "Normokalemic Periodic Paralysis"[tiab] OR "Ocular Muscular Dystrophy"[tiab] OR "oculocraniosomatic disease"[tiab] OR "oculocraniosomatic syndrome"[tiab] OR "Oculocraniosomatic Syndromes"[tiab] OR "Oculopharyngeal"[tiab] OR "olivocerebellar atrophy"[tiab] OR "Olivopontocerebellar Atrophies"[tiab] OR "Olivopontocerebellar Atrophy"[tiab] OR "Olivopontocerebellar"[tiab] OR "Olivo-Ponto-Cerebellar"[tiab] OR "OPCA with Retinal Degeneration"[tiab] OR "Ophthalmoplegia Plus Syndrome"[tiab] OR "Ophthalmoplegia Plus Syndromes"[tiab] OR "Optic Atrophy 1"[tiab] OR "optic atrophy type 1"[tiab] OR "ossifying fibrodysplasia"[tiab] OR "Ossifying myositis"[tiab] OR "Pantothenate Kinase Associated Neurodegeneration"[tiab] OR "Pantothenate Kinase-Associated Neurodegeneration"[tiab] OR "paralysis agitans"[tiab] OR "paramyotonia congenita"[tiab] OR "Parkinson"[tiab] OR "Parkinson's"[tiab] OR "Pelizaeus Merzbacher"[tiab] OR "Pelizaeus-Merzbacher Disease"[tiab] OR "periodic hypokalaemic paralysis"[tiab] OR "periodic hypokalemic paralysis"[tiab] OR "Periodic Paralysis Hypokalemic"[tiab] OR "Periodic Paralysis- Hypokalemic"[tiab] OR "peroneal atrophy"[tiab] OR "peroneal muscle atrophy"[tiab] OR "Peroneal Muscular Atrophies"[tiab] OR "peroneal muscular atrophy"[tiab] OR "Pfkm Deficiency"[tiab] OR "Phytanic Acid Oxidase Deficiency"[tiab] OR "phytanic acid storage disease"[tiab] OR "Pick complex"[tiab] OR "Pick dementia"[tiab] OR "Pick

Disease"[tiab] OR "Pick syndrome"[tiab] OR "Pick's complex"[tiab] OR "Picks Disease"[tiab] OR "Pick's disease"[tiab] OR "Pleoconial Myopathy"[tiab] OR "PNFA"[tiab] OR "PPA Syndrome"[tiab] OR "PPA Syndromes"[tiab] OR "Presenile Dementia"[tiab] OR "Primary Adhalinopathies"[tiab] OR "Primary Adhalinopathy"[tiab] OR "primary dysautonomia"[tiab] OR "primary dysautonomias"[tiab] OR "Primary Parkinsonism"[tiab] OR "Primary Progressive Nonfluent Aphasia"[tiab] OR "pringle bourneville disease"[tiab] OR "pringle disease"[tiab] OR "pringle syndrome"[tiab] OR "Progeria-Like Syndrome"[tiab] OR "Progeria-Like Syndromes"[tiab] OR "progressive external ophthalmoplegia"[tiab] OR "progressive lenticular degeneration"[tiab] OR "Progressive Muscular Atrophies"[tiab] OR "Progressive Muscular Atrophy"[tiab] OR "Progressive nonfluent aphasia"[tiab] OR "Progressive non-fluent aphasia"[tiab] OR "PROMM"[tiab] OR "Proximal Myotonic Myopathies"[tiab] OR "proximal myotonic myopathy"[tiab] OR "Pseudohypertrophic Muscular Dystrophy"[tiab] OR "Pseudomyotonia"[tiab] OR "Pseudosclerosis"[tiab] OR "Psychosine Lipidosis"[tiab] OR "Quantal Squander"[tiab] OR "Ramsay Hunt "[tiab] OR "Recklinghausen disease"[tiab] OR "Recklinghausens Disease"[tiab] OR "Recklinghausen's Disease"[tiab] OR "Refsum Disease"[tiab] OR "Refsum"[tiab] OR "Refsum's"[tiab] OR "Refsums"[tiab] OR "Ricker"[tiab] OR "Riley-Day Syndrome"[tiab] OR "Rod Myopathies"[tiab] OR "Rod Myopathy"[tiab] OR "Rod-Body Myopathy"[tiab] OR "Roussy Levy"[tiab] OR "Santavuori-Haltia"[tiab] OR "Sarcoglycanopathies"[tiab] OR "Sarcoglycanopathy"[tiab] OR "SBMA"[tiab] OR "SCA1"[tiab] OR "SCA1s"[tiab] OR "SCA2"[tiab] OR "SCA3"[tiab] OR "SCA4"[tiab] OR "SCA5"[tiab] OR "SCA6"[tiab] OR "SCA7"[tiab] OR "Scapulo-ilio-peroneal"[tiab] OR "Scapuloperoneal"[tiab] OR "Sclerosis Tuberosa"[tiab] OR "sebaceous adenoma"[tiab] OR "Semantic Dementia"[tiab] OR "Senile Dementia"[tiab] OR "Shy Drager syndrome"[tiab] OR "Spastic Paraplegia 2"[tiab] OR "Spastic Paraplegia Type 2"[tiab] OR "Spielmeyer-Sjogren"[tiab] OR "Spielmeyer-Vogt"[tiab] OR "Spinal Amyotrophies"[tiab] OR "Spinal Amyotrophy"[tiab] OR "spinal and bulbar muscular atrophy"[tiab] OR "spinal muscle atrophy"[tiab] OR "Spinal Muscular Atrophy"[tiab] OR "spinobulbar muscular atrophy"[tiab] OR "Spinocerebellar Ataxia"[tiab] OR "Spinocerebellar Ataxias"[tiab] OR "Spinocerebellar Atrophies"[tiab] OR "spinocerebellar atrophy"[tiab] OR "Spinocerebellar Degeneration"[tiab] OR "spinocerebellar degenerations"[tiab] OR "Spongiform Leukodystrophy"[tiab] OR "Spongy Degeneration"[tiab] OR "Spongy Disease "[tiab] OR "Steinert"[tiab] OR "Steinerts"[tiab] OR "Steinert's"[tiab] OR "Struempell Lorrain"[tiab] OR "Struempell"[tiab] OR "Strumpel"[tiab] OR "Strumpell Lorrain"[tiab] OR "Strumpell"[tiab] OR "Strumpell's"[tiab] OR "Strumpel's"[tiab] OR "sulfatide lipidosis"[tiab] OR "sulfatidosis"[tiab] OR "sulphatide lipidosis"[tiab] OR "Syndrome of Continuous Muscle Activity"[tiab] OR "Tangier Disease"[tiab] OR "tarui disease"[tiab] OR "Tarui's Disease"[tiab] OR "TDP 43 pathology"[tiab] OR "TDP 43 proteinopathies"[tiab] OR "TDP-43 Proteinopathy"[tiab] OR "Thomsen Disease"[tiab] OR "Thomsen myotonia"[tiab] OR "Thomsen's Disease"[tiab] OR "Thomsen's myotonia"[tiab] OR "Tibial Muscular Dystrophy"[tiab] OR "Torsion dystonia"[tiab] OR "Torsion Dystonias"[tiab] OR "torsion spasm"[tiab] OR "Tourette Syndrome"[tiab] OR "Tourette"[tiab] OR "Tourette's"[tiab] OR "Tourettes"[tiab] OR "tuberose sclerosis"[tiab] OR "Tuberous Sclerosis"[tiab] OR "Udd Myopathy"[tiab] OR "Unverricht Disease"[tiab] OR "Unverricht Lundborg Syndrome"[tiab] OR "Unverricht-Lundborg Disease"[tiab] OR "Unverricht-Lundborg Syndrome"[tiab] OR "unverricht-lundborg"[tiab] OR "Van Bogaert-Bertrand Syndrome"[tiab] OR "Vogt-Spielmeyer Disease"[tiab] OR "Wadia Swami Syndrome"[tiab] OR "Wadia-Swami Syndrome"[tiab] OR "Watson Syndrome"[tiab] OR "Werdnig Hoffman"[tiab] OR "Werdnig-Hoffmann"[tiab] OR "Westphall Disease"[tiab] OR "Westphal-Strumpell"[tiab] OR "Wilhelmsen-Lynch Disease"[tiab] OR "Wilson disease"[tiab] OR "Wilson diseases"[tiab] OR "wilson syndrome"[tiab] OR "Wilsons Disease"[tiab] OR "Wilson's disease"[tiab] OR "Wolfram Syndrome"[tiab] OR "X ALD"[tiab] OR "ziehen oppenheim"[tiab] OR (Divry[tiab] AND bogaert[tiab]) OR (Motor[tiab] AND "Vocal Tic"[tiab]) OR (Myoencephalopathy[tiab] AND "lactic acidosis"[tiab] AND stroke[tiab]) OR (Schilder[tiab] AND Addison[tiab]) OR "Transthyretin amyloidosis"[tiab] OR "TTR amyloidosis"[tiab])) OR (Alzheimer Disease [mesh] OR Ataxia Telangiectasia [mesh] OR Heredodegenerative Disorders, Nervous System [mesh] OR Insomnia, Fatal Familial [mesh] OR Isaacs Syndrome [mesh] OR Lewy Body Disease [mesh] OR Mitochondrial Myopathies [mesh] OR Motor Neuron Disease [mesh] OR Multiple System Atrophy [mesh] OR Muscular Disorders, Atrophic [mesh] OR Myasthenia Gravis [mesh] OR Myasthenic Syndromes, Congenital [mesh] OR Myopathies, Structural, Congenital [mesh] OR Myositis [mesh] OR Myotonic Disorders [mesh] OR Pain Insensitivity,

Congenital [mesh] OR Paralyses, Familial Periodic [mesh] OR Parkinson Disease [mesh] OR Pick disease of the brain [mesh] OR Polyneuropathies [mesh] OR TDP-43 Proteinopathies [mesh]))

Search component #3’: Animal population [2]

(“animal experimentation”[MeSH Terms] OR “models, animal”[MeSH Terms] OR “invertebrates”[MeSH Terms] OR “Animals”[Mesh:noexp] OR “animal population groups”[MeSH Terms] OR “chordata”[MeSH Terms:noexp] OR “chordata, nonvertebrate”[MeSH Terms] OR “vertebrates”[MeSH Terms:noexp] OR “amphibians”[MeSH Terms] OR “birds”[MeSH Terms] OR “fishes”[MeSH Terms] OR “reptiles”[MeSH Terms] OR “mammals”[MeSH Terms:noexp] OR “primates”[MeSH Terms:noexp] OR “artiodactyla”[MeSH Terms] OR “carnivora”[MeSH Terms] OR “cetacea”[MeSH Terms] OR “chiroptera”[MeSH Terms] OR “elephants”[MeSH Terms] OR “hyraxes”[MeSH Terms] OR “insectivora”[MeSH Terms] OR “lagomorpha”[MeSH Terms] OR “marsupialia”[MeSH Terms] OR “monotremata”[MeSH Terms] OR “perissodactyla”[MeSH Terms] OR “rodentia”[MeSH Terms] OR “scandentia”[MeSH Terms] OR “sirenia”[MeSH Terms] OR “xenarthra”[MeSH Terms] OR “haplorhini”[MeSH Terms:noexp] OR “strepsirhini”[MeSH Terms] OR “platyrrhini”[MeSH Terms] OR “tarsii”[MeSH Terms] OR “catarrhini”[MeSH Terms:noexp] OR “cercopithecidae”[MeSH Terms] OR “hylobatidae”[MeSH Terms] OR “hominidae”[MeSH Terms:noexp] OR “gorilla gorilla”[MeSH Terms] OR “pan paniscus”[MeSH Terms] OR “pan troglodytes”[MeSH Terms] OR “pongo pygmaeus”[MeSH Terms]) OR ((animals[tiab] OR animal[tiab] OR mice[Tiab] OR mus[Tiab] OR mouse[Tiab] OR murine[Tiab] OR woodmouse[tiab] OR rats[Tiab] OR rat[Tiab] OR murinae[Tiab] OR muridae[Tiab] OR cottonrat[tiab] OR cottonrats[tiab] OR hamster[tiab] OR hamsters[tiab] OR cricetinae[tiab] OR rodentia[Tiab] OR rodent[Tiab] OR rodents[Tiab] OR pigs[Tiab] OR pig[Tiab] OR swine[tiab] OR swines[tiab] OR piglets[tiab] OR piglet[tiab] OR boar[tiab] OR boars[tiab] OR “sus scrofa”[tiab] OR ferrets[tiab] OR ferret[tiab] OR polecat[tiab] OR polecats[tiab] OR “mustela putorius”[tiab] OR “guinea pigs”[Tiab] OR “guinea pig”[Tiab] OR cavia[Tiab] OR callithrix[Tiab] OR marmoset[Tiab] OR marmosets[Tiab] OR cebuella[Tiab] OR hapale[Tiab] OR octodon[Tiab] OR chinchilla[Tiab] OR chinchillas[Tiab] OR gerbillinae[Tiab] OR gerbil[Tiab] OR gerbils[Tiab] OR jird[Tiab] OR jirds[Tiab] OR merione[Tiab] OR meriones[Tiab] OR rabbits[Tiab] OR rabbit[Tiab] OR hares[Tiab] OR hare[Tiab] OR diptera[Tiab] OR flies[Tiab] OR fly[Tiab] OR dipteral[Tiab] OR drosophila[Tiab] OR drosophilidae[Tiab] OR cats[Tiab] OR cat[Tiab] OR carus[Tiab] OR felis[Tiab] OR nematoda[Tiab] OR nematode[Tiab] OR nematodes[Tiab] OR sipunculida[Tiab] OR dogs[Tiab] OR dog[Tiab] OR canine[Tiab] OR canines[Tiab] OR canis[Tiab] OR sheep[Tiab] OR sheeps[Tiab] OR mouflon[Tiab] OR mouflons[Tiab] OR ovis[Tiab] OR goats[Tiab] OR goat[Tiab] OR capra[Tiab] OR capras[Tiab] OR rupicapra[Tiab] OR rupicapras[Tiab] OR chamois[Tiab] OR haplorhini[Tiab] OR monkey[Tiab] OR monkeys[Tiab] OR anthropoidea[Tiab] OR anthropoids[Tiab] OR saguinus[Tiab] OR tamarin[Tiab] OR tamarins[Tiab] OR leontopithecus[Tiab] OR hominidae[Tiab] OR ape[Tiab] OR apes[Tiab] OR “pan paniscus”[Tiab] OR bonobo[Tiab] OR bonobos[Tiab] OR “pan troglodytes”[Tiab] OR gibbon[Tiab] OR gibbons[Tiab] OR siamang[Tiab] OR siamangs[Tiab] OR nomascus[Tiab] OR symphalangus[Tiab] OR chimpanzee[Tiab] OR chimpanzees[Tiab] OR prosimian[Tiab] OR prosimians[Tiab] OR “bush baby”[Tiab] OR bush babies[Tiab] OR galagos[Tiab] OR galago[Tiab] OR pongidae[Tiab] OR gorilla[Tiab] OR gorillas[Tiab] OR “pongo pygmaeus”[Tiab] OR orangutan[Tiab] OR orangutans[Tiab] OR lemur[Tiab] OR lemurs[Tiab] OR lemuridae[Tiab] OR horse[Tiab] OR horses[Tiab] OR equus[Tiab] OR cow[Tiab] OR calf[Tiab] OR bull[Tiab] OR chicken[Tiab] OR chickens[Tiab] OR gallus[Tiab] OR quail[Tiab] OR bird[Tiab] OR birds[Tiab] OR quails[Tiab] OR poultry[Tiab] OR poultries[Tiab] OR fowl[Tiab] OR fowls[Tiab] OR reptile[Tiab] OR reptilia[Tiab] OR reptiles[Tiab] OR snakes[Tiab] OR snake[Tiab] OR lizard[Tiab] OR lizards[Tiab] OR alligator[Tiab] OR alligators[Tiab] OR crocodile[Tiab] OR crocodiles[Tiab] OR turtle[Tiab] OR turtles[Tiab] OR amphibian[Tiab] OR amphibians[Tiab] OR amphibia[Tiab] OR frog[Tiab] OR frogs[Tiab] OR bombina[Tiab] OR salientia[Tiab] OR toad[Tiab] OR toads[Tiab] OR “epidalea calamita”[Tiab] OR salamander[Tiab] OR salamanders[Tiab] OR eel[Tiab] OR eels[Tiab] OR fish[Tiab] OR fishes[Tiab] OR pisces[Tiab] OR catfish[Tiab] OR catfishes[Tiab] OR siluriformes[Tiab] OR arius[Tiab] OR heteropneustes[Tiab] OR sheatfish[Tiab] OR perch[Tiab] OR perches[Tiab] OR percidae[Tiab] OR perca[Tiab] OR trout[Tiab] OR trouts[Tiab] OR char[Tiab] OR chars[Tiab] OR salvelinus[Tiab] OR

minnow[Tiab] OR cyprinidae[Tiab] OR carps[Tiab] OR carp[Tiab] OR zebrafish[Tiab] OR zebrafishes[Tiab] OR goldfish[Tiab] OR goldfishes[Tiab] OR guppy[Tiab] OR guppies[Tiab] OR chub[Tiab] OR chubs[Tiab] OR tinca[Tiab] OR barbels[Tiab] OR barbus[Tiab] OR pimephales[Tiab] OR promelas[Tiab] OR "poecilia reticulata"[Tiab] OR mullet[Tiab] OR mullets[Tiab] OR eel[Tiab] OR eels[Tiab] OR seahorse[Tiab] OR seahorses[Tiab] OR mugil curema[Tiab] OR atlantic cod[Tiab] OR shark[Tiab] OR sharks[Tiab] OR catshark[Tiab] OR anguilla[Tiab] OR salmonid[Tiab] OR salmonids[Tiab] OR whitefish[Tiab] OR whitefishes[Tiab] OR salmon[Tiab] OR salmons[Tiab] OR sole[Tiab] OR solea[Tiab] OR lamprey[Tiab] OR lampreys[Tiab] OR pumpkinseed[Tiab] OR sunfish[Tiab] OR sunfishes[Tiab] OR tilapia[Tiab] OR tilapias[Tiab] OR turbot[Tiab] OR turbots[Tiab] OR flatfish[Tiab] OR flatfishes[Tiab] OR sciuridae[Tiab] OR squirrel[Tiab] OR squirrels[Tiab] OR chipmunk[Tiab] OR chipmunks[Tiab] OR suslik[Tiab] OR susliks[Tiab] OR vole[Tiab] OR voles[Tiab] OR lemming[Tiab] OR lemmings[Tiab] OR muskrat[Tiab] OR muskrats[Tiab] OR lemmus[Tiab] OR otter[Tiab] OR otters[Tiab] OR marten[Tiab] OR martens[Tiab] OR martes[Tiab] OR weasel[Tiab] OR badger[Tiab] OR badgers[Tiab] OR ermine[Tiab] OR mink[Tiab] OR minks[Tiab] OR sable[Tiab] OR sables[Tiab] OR gulo[Tiab] OR gulos[Tiab] OR wolverine[Tiab] OR wolverines[Tiab] OR mustela[Tiab] OR llama[Tiab] OR llamas[Tiab] OR alpaca[Tiab] OR alpacas[Tiab] OR camelid[Tiab] OR camelids[Tiab] OR guanaco[Tiab] OR guanacos[Tiab] OR chiroptera[Tiab] OR chiropteras[Tiab] OR bat[Tiab] OR bats[Tiab] OR fox[Tiab] OR foxes[Tiab] OR iguana[Tiab] OR iguanas[Tiab] OR xenopus laevis[Tiab] OR parakeet[Tiab] OR parakeets[Tiab] OR parrot[Tiab] OR parrots[Tiab] OR donkey[Tiab] OR donkeys[Tiab] OR mule[Tiab] OR mules[Tiab] OR zebra[Tiab] OR zebras[Tiab] OR shrew[Tiab] OR shrews[Tiab] OR bison[Tiab] OR bisons[Tiab] OR buffalo[Tiab] OR buffaloes[Tiab] OR deer[Tiab] OR deers[Tiab] OR bear[Tiab] OR bears[Tiab] OR panda[Tiab] OR pandas[Tiab] OR "wild hog"[Tiab] OR "wild boar"[Tiab] OR fitchew[Tiab] OR fitch[Tiab] OR beaver[Tiab] OR beavers[Tiab] OR jerboa[Tiab] OR jerboas[Tiab] OR capybara[Tiab] OR capybaras[Tiab]) NOT medline[sb])

PubMed Search Strategy: ((#1' AND #2') AND #3'))

3. Web of Science

Search component #1": Intervention

TS=(AAV OR Adeno-associated OR Cationic lipid OR Cationic lipids OR Cell-penetrating peptide OR Cell-penetrating peptides OR Chitosan OR Conjugate OR Conjugated OR Conjugates OR Conjugation OR Copolymer OR Co-polymer OR Co-polymers OR Copolymers OR CPP OR CPPs OR Dendrimer OR Dendrimers OR Dendriplex OR Dendriplexes OR Excipient OR Excipients OR Formulation OR Formulations OR Lipid OR Lipids OR Lipofectamine OR Lipofection OR Lipoplex OR Lipoplexes OR liposome OR liposomes OR Nanoballs OR Nanocapsule OR Nanocapsules OR Nanocarrier OR Nanocarriers OR Nanocomposite OR Nanocomposites OR Nanoconjugate OR Nanoconjugates OR Nanocrystal OR Nanocrystals OR nanoparticle OR nanoparticles OR Nano-particulate OR Nanorod OR Nanorods OR Nanotube OR Nanotubes OR Nano-ball OR Nano-balls OR Nano-capsule OR Nano-capsules OR Nano-carrier OR Nano-carriers OR Nano-composite OR Nano-composites OR Nano-conjugate OR Nano-conjugates OR Nano-crystal OR Nano-crystals OR Nano-particle OR Nano-particles OR Nano-particulate OR Nano-rod OR Nano-rods OR Nano-tube OR Nano-tubes OR Oligoplex OR Oligoplexes OR PEI OR peptide OR peptide-linked OR peptides OR PLGA OR Polyelectrolyte OR Polyelectrolytes OR Polyethylenimine OR polylactic-co-glycolic acid OR Polymer OR Polymers OR Polymersomes OR Polyplex OR Polyplexes OR Protamine OR Silica OR Thiomer OR Thiomers OR Transfection OR Vector OR Vectorized OR Vectors OR Viral OR Virosome OR Virosomes OR Virus OR Viruses OR Virus-like particle OR Virus-like particles OR VLP OR VLPs) AND TS=(antisense OR AON OR AONs OR ASO OR ASOs OR LNA OR LNAs OR Locked nucleic acid OR Locked nucleic acids OR MORF OR MORFs OR Morpholino OR Morpholinos OR Peptide nucleic acid OR Peptide nucleic acids OR Phosphorothioate OR Phosphorothioates OR PMO OR PMOs OR PNA OR PNAs OR SCO OR SCOs OR splice-correcting oligonucleotide OR splice-correcting oligonucleotides OR splice-switching oligonucleotide OR splice-switching oligonucleotides OR SSO OR SSOs)

Search component #2": Disease

TS=((degenerative AND nervous system) OR (degenerative AND neurologic) OR (degenerative AND neurological) OR (degenerative AND spinal cord) OR fasciculation OR neurodegenerative OR neuromuscular) AND TS=(autosomal OR congenital OR dominant OR familial OR genealogical OR genetic OR hereditary OR heritable OR inborn OR inheritable OR inheritance OR inherited OR recessive OR single-gene OR X-linked) OR TS=("Charcot Marie Tooth" OR "Childhood Torsion Disease" OR "X ALD" OR (Divry AND bogaert) OR (Myoencephalopathy AND "lactic acidosis" AND stroke) OR (Schilder AND Addison) OR (Siemerling AND Creutzfeldt) OR "Adenoma Sebaceum" OR "adreno leucodystrophy" OR "adreno leukodystrophy" OR "adrenoleucodystrophy" OR "Adrenoleukodystrophy" OR "adrenoleukomyeloneuropathy" OR "adrenomyeloneuropathy" OR "Alexander Disease" OR "alexander syndrome" OR "Alexanders Disease" OR "Alexander's Disease" OR "Alstrom Hallgren Syndrome" OR "Alstrom Syndrome" OR "Alström Syndrome" OR "Alstrom-Hallgren Syndrome" OR "Alstroms Syndrome" OR "Alstrom's Syndrome" OR "Alzeimer's" OR "Alzheimer" OR "Alzheimers" OR "amaurotic familial idiocy" OR "amaurotic idiocy" OR "amyloid neuropathies" OR "Amyloid Neuropathy" OR "Amyloid Polyneuropathies" OR "amyloid polyneuropathy" OR "amyloidotic neuropathy" OR "amyloidotic polyneuropathy" OR "Amyotrophic Lateral Sclerosis" OR "analgesia congenita" OR "Analphalipoproteinemia" OR "Anterior Horn Cell Disease" OR "ARSA Deficiency" OR "Arylsulfatase A Deficiencies" OR "Arylsulfatase A Deficiency" OR "ASP Deficiency" OR "ASPA Deficiency" OR "Aspartoacylase Deficiency" OR "Ataxia Telangiectasia Syndrome" OR "Ataxia Telangiectasia" OR "ataxia telangiectatica" OR "ataxia teleangiectasia" OR "Autosomal Dominant Cerebellar Ataxia, Type II" OR "Autosomal Dominant Optic Atrophy" OR "Autosomal Dominant Spastic Paraplegia" OR "Autosomal Dominant Striatonigral Degeneration" OR "Autosomal Recessive Spastic Paraplegia" OR "Azorean Disease" OR "balo disease" OR "Baltic Myoclonus" OR "bar syndrome" OR "Batten" OR "Becker Disease" OR "Becker Muscular Dystrophy" OR "Becker's Muscular Dystrophy" OR "beta-Galactosidase

Deficiencies" OR "beta-Galactosidase Deficiency" OR "Bilateral Acoustic Neurinoma" OR "Bilateral Acoustic Neurinomas" OR "Bilateral Acoustic Neuroma" OR "Bilateral Acoustic Neuromas" OR "Bilateral Acoustic Schwannoma" OR "Bilateral Acoustic Schwannomas" OR "bourneville disease" OR "Bourneville Phakomatosis" OR "bourneville pringle syndrome" OR "Bourneville Pringle's Disease" OR "bourneville syndrome" OR "bourneville tuberous sclerosis" OR "Bourneville-Pringle Disease" OR "Bourneville-Pringles Disease" OR "Bourneville-Pringle's Disease" OR "Bourneville's Disease" OR "Bourneville's Syndrome" OR "brain sclerosis" OR "bulbar and spinal muscular atrophy" OR "bulbar dysfunction" OR "bulbar muscle weakness" OR "Bulbar palsies" OR "Bulbar Palsy" OR "bulbar paralyses" OR "bulbar paralysis" OR "bulbar sign" OR "bulbar symptom" OR "bulbar syndrome" OR "bulbar weakness" OR "Bulbospinal atrophy" OR "Bulbo-Spinal Atrophy" OR "bulbospinal muscular atrophy" OR "bulbo-spinal muscular atrophy" OR "Bulbospinal Neuronopathy" OR "Canavan Disease" OR "canavan syndrome" OR "Canavan's disease" OR "Canavan-van Bogaert-Bertrand Disease" OR "Central core disease" OR "Central Core Diseases" OR "Central Core Myopathies" OR "Central Core Myopathy" OR "Cerebellar Degeneration with Slow Eye Movements" OR "cerebellar dyssynergia" OR "Cerebral Scleroses" OR "cerebral sclerosis" OR "Cerebroside Sulphatase Deficiency" OR "Ceroid Storage Disease" OR "Ceroid Storage Diseases" OR "Channelopathy-Associated Insensitivity To Pain" OR "Charcot Disease" OR "charcot marie disease" OR "Cholesterol Thesaurismosis" OR "chorea major" OR "chronic progressive chorea" OR "Chronic Progressive External Ophthalmoplegia" OR "Chronic Progressive Hereditary Chorea" OR "Circumscribed Lobar Atrophy of the Brain" OR "Cockayne Syndrome" OR "Cockayne" OR "Cockayne's" OR "concentric sclerosis" OR "congenital analgesia" OR "congenital anesthesia" OR "congenital atonic sclerotic muscular dystrophy" OR "congenital generalized analgesia" OR "congenital indifference to pain" OR "congenital insensitivity to pain" OR "Congenital Myopathy" OR "Congenital Myotonia" OR "Congenital Pain Insensitivity" OR "congenital paramyotonia" OR "Continuous Muscle Activity Syndrome" OR "Copper Storage Disease" OR "CPEO with Ragged Red Fibers" OR "cranial nerve paralysis" OR "DDPAC" OR "dejerine sottas" OR "Dejerine-Thomas" OR "dementia with Lewy bodies" OR "dementia with Lewy body" OR "Dementia with Lobar Atrophy and Neuronal Cytoplasmic Inclusions" OR "DIDMOAD" OR "Dilated Cardiomyopathy" OR "Disinhibition-Dementia-Parkinsonism-Amyotrophy" OR "Distal 1 Myopathy" OR "Distal Hereditary Myopathy" OR "Distal Muscular Dystrophies" OR "Distal Muscular Dystrophy" OR "Distal Myopathies" OR "Distal Myopathy" OR "Dominant Hereditary Sensory Neuropathy" OR "Dominant Optic Atrophies" OR "dominant optic atrophy" OR "Duchenne" OR "dysantonomia" OR "Dysmyelinogenic Leukodystrophy" OR "Dyssynergia Cerebellaris " OR "Dystonia Musculorum Deformans" OR "dystrophia myotonica" OR "dystrophic myotonia" OR "Emery-Dreifuss" OR "encephalitis periaxialis" OR "epiloia" OR "Eulenburg" OR "Facioscapulohumeral" OR "Facio-Scapulo-Humeral" OR "Facioscapuloperoneal" OR "Familial Acoustic Neuromas" OR "familial autonomic dysfunction" OR "Familial dysautonomia" OR "familial dysautonomy" OR "Familial Fatal Insomnia" OR "Familial Periodic Paralyses" OR "Familial Periodic Paralysis" OR "Familial Polyneuropathies" OR "Familial Polyneuropathy" OR "familial spastic paralysis" OR "familial spastic paraparesis" OR "familial spastic paraplegia" OR "Fatal Familial Insomnia" OR "Fazio-Londe Disease" OR "Fazio-Londe Syndrome" OR "Fibrinoid Degeneration of Astrocytes" OR "Fibrodysplasia Ossificans" OR "Friedreich Ataxia" OR "Friedreich" OR "Friedreich's" OR "Friedreichs" OR "frontal dementia" OR "frontal lobe dementia" OR "Frontotemporal Dementia" OR "frontotemporal dementias" OR "Frontotemporal Lobar Degeneration" OR "Frontotemporal Lobe Dementia" OR "FTD" OR "FTDP" OR "FTLD" OR "Fukuhara disease" OR "Galactocerebrosidase Deficiency" OR "Galactosylceramidase Deficiency" OR "galactosylceramide lipidosis" OR "GALC Deficiency" OR "Gehrig Disease" OR "Gehrigs Disease" OR "Gehrig's Disease" OR "Generalized Myotonia" OR "Generalized Myotonias" OR "Gerstmann-Straussler" OR "Gerstmann-Straussler-Scheinker Disease" OR "Giant Axonal Neuropathy" OR "globoid cell dystrophy" OR "globoid cell leucodystrophy" OR "Globoid Cell Leukodystrophy" OR "Globoid Leukodystrophies" OR "Globoid Leukodystrophy" OR "Glycogen Storage Disease Type VII" OR "Glycogen Storage Disease VII" OR "Graefe's Disease" OR "Greenfield Disease" OR "Greenfield's Disease" OR "GSD VII" OR "GSS" OR "Guam Disease" OR "Hallervorden Spatz" OR "Hallervorden-Spatz" OR "Hauptmann-Thannhauser" OR "HDDD1" OR "HDDD2" OR "hemimegalencephaly" OR "hepatocerebral degeneration" OR "Hepatolenticular Degeneration" OR "hepatolenticular syndrome" OR

"Hereditary Areflexic Dystasia" OR "hereditary chorea" OR "Hereditary Dysphasic Disinhibition Dementia" OR "hereditary motor and sensory neuropathies" OR "Hereditary Motor and Sensory Neuropathy " OR "Hereditary Motor And Sensory Neuropathy V" OR "Hereditary Motor Neuronopathies" OR "Hereditary Motor Neuronopathy" OR "hereditary motor sensory neuropathy type IV" OR "hereditary motor sensory neuropathy" OR "hereditary myotonia" OR "Hereditary Optic Atrophies" OR "Hereditary Optic Atrophy" OR "hereditary optic nerve atrophy" OR "Hereditary Optic Neuroretinopathy" OR "hereditary sensorimotor neuropathy" OR "Hereditary Sensory and Autonomic Neuropathy" OR "hereditary sensory and motor neuropathy" OR "Hereditary Sensory Neuropathy" OR "hereditary spastic paralysis" OR "hereditary spastic paraparesis" OR "Hereditary Spastic Paraplegia" OR "Hereditary Spastic Paraplegias" OR "hereditary spinal ataxia" OR "Hereditary Spinal Sclerosis" OR "Hereditary Type I Motor and Sensory Neuropathy" OR "heredopathia atactica polyneuritiformis" OR "High Density Lipoprotein Deficiency" OR "HMSN 5" OR "HMSN Type IV" OR "HMSN Type V" OR "HMSN V" OR "HMSN" OR "HOKPP" OR "HSAN III" OR "HSAN Type III" OR "HSAN3" OR "HSN-III" OR "Huntington disease" OR "Huntington" OR "Huntington's" OR "Hyperkalemic Periodic Paralysis" OR "hypokalaemic periodic paralysis" OR "Hypokalemic Periodic Paralysis" OR "HYPOKPP" OR "hypomyelinating Leukodystrophy" OR "HYPOPP" OR "Inclusion Body Myopathy" OR "Inclusion Body Myositides" OR "Inclusion Body Myositis" OR "Infantile Muscular Atrophies" OR "Infantile Muscular Atrophy" OR "Inherited Polyneuropathies" OR "Inherited Polyneuropathy" OR "Isaac Syndrome" OR "Isaacs Mertens Syndrome" OR "Isaacs Syndrome" OR "Isaacs' Syndrome" OR "Isaacs-Mertens Syndrome" OR "Jansky-Bielschowsky" OR "Joseph Azorean Disease" OR "joseph disease" OR "juvenile amaurotic family idiocy" OR "juvenile amaurotic idiocy" OR "Juvenile Cerebroretinal Degeneration" OR "juvenile chorea" OR "Juvenile Muscular Atrophies" OR "Juvenile Muscular Atrophy" OR "Juvenile Optic Atrophy" OR "Juvenile-Onset Neuroaxonal Dystrophy" OR "Kearn Syndrome" OR "Kearne-Sayre" OR "Kearns syndrome" OR "Kearns' Syndrome" OR "Kearn-Sayre" OR "Kearns-Sayer" OR "Kearns-Sayre Syndrome" OR "Kearns-Sayre" OR "Kennedy Disease" OR "Kennedy Syndrome" OR "Kennedys Disease" OR "Kennedy's Disease" OR "Kennedy's syndrome" OR "Kjer Optic Atrophy" OR "Kjer's Optic Atrophy" OR "Kjer-Type Optic Atrophy" OR "Krabbe" OR "Krabbes" OR "Krabbe's" OR "Kuf Disease" OR "Kufs Disease" OR "Kuf's Disease" OR "Kugelberg-Welander" OR "Lafora Disease" OR "Lafora" OR "Landouzy-Dejerine" OR "Lateral Scleroses" OR "Lateral Sclerosis" OR "Leber Disease" OR "Leber Hereditary Optic Atrophy" OR "Leber Hereditary optic atrophy" OR "Leber Hereditary optic neuropathy" OR "Leber optic atrophy" OR "Leber Optic Neuropathy" OR "Lebers Disease" OR "Leber's Disease" OR "Leber's Diseases" OR "Leber's Hereditary Optic Atrophy" OR "Leber's Hereditary Optic Neuropathy" OR "Leber's Optic Atrophy" OR "Lebers Optic Neuropathy" OR "Leber's Optic Neuropathy" OR "Leukodystrophy with Rosenthal Fibers" OR "Lewy body dementia" OR "Lewy body dementias" OR "Lewy Body Disease" OR "Lewy body diseases" OR "Limb-Girdle" OR "Lipofuscin Storage Disease" OR "Lobar Atrophy Of Brain" OR "Lobar Atrophy of the Brain" OR "Lobe Dementia, Frontotemporal" OR "Luft Disease" OR "Lufts Disease" OR "Luft's Disease" OR "lundborg syndrome" OR "machado disease" OR "Machado-Joseph Disease" OR "Machado-Joseph" OR "macrencephaly" OR "Megaconial Myopathy" OR "megalencephalic leukodystrophy" OR "megalencephaly" OR "melanodermic leukodystrophy" OR "MELAS Syndrome" OR "MELAS" OR "Menzel Type OPCA" OR "MERRF Syndrome" OR "MERRF" OR "metachromatic leucodystrophy" OR "metachromatic leucoencephalopathy" OR "Metachromatic Leukodystrophies" OR "Metachromatic Leukodystrophy" OR "metachromatic leukoencephalopathy" OR "Mitochondrial Encephalomyopathies" OR "Mitochondrial Encephalomyopathy" OR "Mitochondrial Myopathies" OR "Mitochondrial Myopathy" OR "Mitochondrial Ocular Myopathy" OR "Mitochondrial-Encephalopathy-Lactic Acidosis-Stroke" OR "Molluscum Fibrosum" OR "morbus wilson" OR "motoneuron disease" OR "Motor Neuron Disease" OR "Motor Neuron Diseases" OR "motor neurone disease" OR "motor system disease" OR "Motor System Diseases" OR "multiple mucosal neuroma" OR "multiple neurofibroma" OR "Multiple Neurofibromas" OR "multiple neuroma" OR "Multiple System Atrophies" OR "Multiple System Atrophy" OR "Multiple System Tauopathy with Presenile Dementia" OR "Multisystem Atrophies" OR "Multisystem Atrophy" OR "Multisystemic Atrophies" OR "Multisystemic Atrophy" OR "muscle dystrophy" OR "Muscle Phosphofructokinase Deficiency" OR "muscular dystrophia" OR "Muscular Dystrophies" OR "Muscular Dystrophy" OR "Myelopathic Muscular Atrophy" OR "Myoclonic Cerebellar Dyssynergia" OR "myoclonic

epilepsies" OR "myoclonic epilepsy" OR "Myoclonus Epilepsies" OR "Myoclonus epilepsy" OR "myodystrophia" OR "Myodystrophies" OR "myodystrophy" OR "Myokymia" OR "Myokymias" OR "Myositis Ossificans" OR "myotonia atrophica" OR "Myotonia Congenita" OR "myotonia dystrophica" OR "myotonia hereditaria" OR "Myotonia Levior" OR "myotonic atrophy" OR "myotonic dystrophia" OR "Myotonic Dystrophies" OR "Myotonic Dystrophy" OR "myotonic muscle dystrophy" OR "myotonic muscular dystrophy" OR "NBIA" OR "Nemaline Myopathies" OR "Nemaline Myopathy" OR "Nemaline Rod Disease" OR "neurodegeneration with brain iron accumulation" OR "neurofibromatoses" OR "Neurofibromatosis" OR "neurogenic amyotrophy" OR "neurogenic atrophy" OR "neurogenic muscle atrophy" OR "neurogenic muscular atrophy" OR "neurogenic osteoarthropathy" OR "neurogenic paraosteoarthropathy" OR "Neurogenic Scapuloperoneal Amyotrophy" OR "neuroinomatosis" OR "neuromatosis" OR "neuromyotonia" OR "Neuronal Ceroid-Lipofuscinoses" OR "Neuronal Ceroid-Lipofuscinosis" OR "neuronal ceroidosis" OR "Neuropathic Amyloidosis" OR "Nigrospinodentatal Degeneration" OR "Non fluent Primary Progressive Aphasia" OR "Non-fluent Primary Progressive Aphasia" OR "Nonfluent progressive aphasia" OR "Non-fluent progressive aphasia" OR "Normokalemic Periodic Paralysis" OR "Ocular Muscular Dystrophy" OR "oculocraniosomatic disease" OR "oculocraniosomatic syndrome" OR "Oculocraniosomatic Syndromes" OR "Oculopharyngeal" OR "olivocerebellar atrophy" OR "Olivopontocerebellar Atrophies" OR "Olivopontocerebellar Atrophy" OR "Olivopontocerebellar" OR "Olivo-Ponto-Cerebellar" OR "OPCA with Retinal Degeneration" OR "Ophthalmoplegia Plus Syndrome" OR "Ophthalmoplegia Plus Syndromes" OR "Optic Atrophy 1" OR "optic atrophy type 1" OR "ossifying fibrodysplasia" OR "Ossifying myositis" OR "Pantothenate Kinase Associated Neurodegeneration" OR "Pantothenate Kinase-Associated Neurodegeneration" OR "paralysis agitans" OR "paramyotonia congenita" OR "Parkinson" OR "Parkinson's" OR "Pelizaeus Merzbacher" OR "Pelizaeus-Merzbacher Disease" OR "periodic hypokalaemic paralysis" OR "periodic hypokalemic paralysis" OR "Periodic Paralysis Hypokalemic" OR "Periodic Paralysis- Hypokalemic" OR "peroneal atrophy" OR "peroneal muscle atrophy" OR "Peroneal Muscular Atrophies" OR "Peroneal Muscular Atrophy with Pyramidal Features" OR "peroneal muscular atrophy" OR "Pfkm Deficiency" OR "Phytanic Acid Oxidase Deficiency" OR "phytanic acid storage disease" OR "Pick complex" OR "Pick dementia" OR "Pick Disease" OR "Pick syndrome" OR "Pick's complex" OR "Picks Disease" OR "Pick's disease" OR "Pleoconial Myopathy" OR "PNFA" OR "Polyneuritic Amyloidosis" OR "PPA Syndrome" OR "PPA Syndromes" OR "Presenile Dementia" OR "Primary Adhalinopathies" OR "Primary Adhalinopathy" OR "primary dysautonomia" OR "primary dysautonomias" OR "Primary Parkinsonism" OR "Primary Progressive Nonfluent Aphasia" OR "Primary Senile Degenerative Dementia" OR "pringle bourneville disease" OR "pringle disease" OR "pringle syndrome" OR "Progeria-Like Syndrome" OR "Progeria-Like Syndromes" OR "progressive external ophthalmoplegia" OR "progressive lenticular degeneration" OR "Progressive Muscular Atrophies" OR "Progressive Muscular Atrophy" OR "Progressive nonfluent aphasia" OR "Progressive non-fluent aphasia" OR "Progressive Torsion Spasm" OR "PROMM" OR "Proximal Myotonic Myopathies" OR "proximal myotonic myopathy" OR "Pseudohypertrophic Muscular Dystrophy" OR "Pseudomyotonia" OR "Pseudosclerosis" OR "Psychosine Lipidosis" OR "Quantal Squander" OR "Ramsay Hunt " OR "Recklinghausen disease" OR "Recklinghausens Disease" OR "Recklinghausen's Disease" OR "Refsum Disease" OR "Refsum" OR "Refsum’s" OR "Refsums" OR "Ricker" OR "Riley-Day Syndrome" OR "Rod Myopathies" OR "Rod Myopathy" OR "Rod-Body Myopathy" OR "Roussy Levy" OR "Santavuori-Haltia" OR "Sarcoglycanopathies" OR "Sarcoglycanopathy" OR "sayre kearns syndrome" OR "SBMA" OR "SCA1" OR "SCA1s" OR "SCA2" OR "SCA3" OR "SCA4" OR "SCA5" OR "SCA6" OR "SCA7" OR "Scapulo-ilio-peroneal" OR "Scapuloperoneal" OR "Sclerosis Tuberosa" OR "sebaceous adenoma" OR "Semantic Dementia" OR "Senile Dementia" OR "Shy Drager syndrome" OR "spastic hereditary paraplegia" OR "Spastic Paraplegia 2" OR "Spastic Paraplegia Type 2" OR "Spielmeyer-Sjogren" OR "Spielmeyer-Vogt" OR "Spinal Amyotrophies" OR "Spinal Amyotrophy" OR "spinal and bulbar muscular atrophy" OR "spinal muscle atrophy" OR "Spinal Muscular Atrophy" OR "spine muscle atrophy" OR "spinobulbar muscular atrophy" OR "Spinocerebellar Ataxia" OR "Spinocerebellar Ataxias" OR "Spinocerebellar Atrophies" OR "spinocerebellar atrophy" OR "Spinocerebellar Degeneration" OR "spinocerebellar degenerations" OR "Spongiform Leukodystrophy" OR "Spongy Degeneration" OR "Spongy Disease " OR "Steinert" OR "Steinerts" OR "Steinert's" OR

"Struempell Lorrain" OR "Struempell" OR "Strumpel" OR "Strumpell Lorrain" OR "Strumpell" OR "Strumpell's" OR "Strumpel's" OR "sulfatide lipidosis" OR "sulfatidosis" OR "sulphatide lipidosis" OR "Syndrome of Continuous Muscle Activity" OR "Tangier Disease" OR "tarui disease" OR "Tarui's Disease" OR "TDP 43 pathology" OR "TDP 43 proteinopathies" OR "TDP-43 Proteinopathy" OR "Thomsen Disease" OR "Thomsen myotonia" OR "Thomsens Disease" OR "Thomsen's Disease" OR "Thomsens myotonia" OR "Thomsen's myotonia" OR "Tibial Muscular Dystrophy" OR "Torsion dystonia" OR "Torsion Dystonias" OR "torsion spasm" OR "Tourette Syndrome" OR "Tourette" OR "Tourette’s" OR "Tourettes" OR "tuberose sclerosis" OR "Tuberous Sclerosis" OR "Udd Myopathy" OR "Unverricht Disease" OR "Unverricht Lundborg Syndrome" OR "Unverricht-Lundborg Disease" OR "Unverricht-Lundborg Syndrome" OR "unverricht-lundborg" OR "Van Bogaert-Bertrand Syndrome" OR "Vogt-Spielmeyer Disease" OR "Wadia Swami Syndrome" OR "Wadia-Swami Syndrome" OR "Watson Syndrome" OR "Werdnig Hoffman" OR "Werdnig-Hoffmann" OR "Westphall Disease" OR "Westphal-Strumpell" OR "Wilhelmsen-Lynch Disease" OR "Wilson disease" OR "Wilson diseases" OR "wilson syndrome" OR "Wilsons Disease" OR "Wilson's disease" OR "Wolfram Syndrome" OR "ziehen oppenheim" OR (Motor AND "Vocal Tic") OR "Transthyretin amyloidosis" OR "TTR amyloidosis")

Search component #3’’: Animal population (courtesy of Alice Tillema, Radboudumc)

TS=(animal OR animals OR pisces OR fish OR fishes OR catfish OR catfishes OR sheatfish OR silurus OR arius OR heteropneustes OR clarias OR gariepinus OR fathead minnow OR fathead minnows OR pimephales OR promelas OR cichlidae OR trout OR trouts OR char OR chars OR salvelinus OR salmo OR oncorhynchus OR guppy OR guppies OR millionfish OR poecilia OR goldfish OR goldfishes OR carassius OR auratus OR mullet OR mullets OR mugil OR curema OR shark OR sharks OR cod OR cods OR gadus OR morhua OR carp OR carps OR cyprinus OR carpio OR killifish OR eel OR eels OR anguilla OR zander OR sander OR lucioperca OR stizostedion OR turbot OR turbots OR psetta OR flatfish OR flatfishes OR plaice OR pleuronectes OR platessa OR tilapia OR tilapias OR oreochromis OR sarotherodon OR common sole OR dover sole OR solea OR zebrafish OR zebrafishes OR danio OR rerio OR seabass OR dicentrarchus OR labrax OR morone OR lamprey OR lampreys OR petromyzon OR pumpkinseed OR pumpkinseeds OR lepomis OR gibbosus OR herring OR clupea OR harengus OR amphibia OR amphibian OR amphibians OR anura OR salientia OR frog OR frogs OR rana OR toad OR toads OR bufo OR xenopus OR laevis OR bombina OR epidalea OR calamita OR salamander OR salamanders OR newt OR newts OR triturus OR reptilia OR reptile OR reptiles OR bearded dragon OR pogona OR vitticeps OR iguana OR iguanas OR lizard OR lizards OR anguis fragilis OR turtle OR turtles OR snakes OR snake OR aves OR bird OR birds OR quail OR quails OR coturnix OR bobwhite OR colinus OR virginianus OR poultry OR poultries OR fowl OR fowls OR chicken OR chickens OR gallus OR zebra finch OR taeniopygia OR guttata OR canary OR canaries OR serinus OR canaria OR parakeet OR parakeets OR grasskeet OR parrot OR parrots OR psittacine OR psittacines OR shelduck OR tadorna OR goose OR geese OR branta OR leucopsis OR woodlark OR lullula OR flycatcher OR ficedula OR hypoleuca OR dove OR doves OR geopelia OR cuneata OR duck OR ducks OR greylag OR graylag OR anser OR harrier OR circus pygargus OR red knot OR great knot OR calidris OR canutus OR godwit OR limosa OR lapponica OR meleagris OR gallopavo OR jackdaw OR corvus OR monedula OR ruff OR philomachus OR pugnax OR lapwing OR peewit OR plover OR vanellus OR swan OR cygnus OR columbianus OR bewickii OR gull OR chroicocephalus OR ridibundus OR albifrons OR great tit OR parus OR aythya OR fuligula OR streptopelia OR risoria OR spoonbill OR platalea OR leucorodia OR blackbird OR turdus OR merula OR blue tit OR cyanistes OR pigeon OR pigeons OR columba OR pintail OR anas OR starling OR sturnus OR owl OR athene noctua OR pochard OR ferina OR cockatiel OR nymphicus OR hollandicus OR skylark OR alauda OR tern OR sterna OR teal OR crecca OR oystercatcher OR haematopus OR ostralegus OR shrew OR shrews OR sorex OR araneus OR crocidura OR russula OR european mole OR talpa OR chiroptera OR bat OR bats OR eptesicus OR serotinus OR myotis OR dasycneme OR daubentonii OR pipistrelle OR pipistrellus OR cat OR cats OR felis OR catus OR feline OR dog OR dogs OR canis OR canine OR canines OR otter OR otters OR lutra OR badger OR badgers OR meles OR fitchew OR fitch OR foumart OR foulmart OR ferrets OR ferret OR polecat OR polecats OR mustela OR putorius OR weasel OR weasels OR fox OR foxes OR vulpes OR common seal OR phoca OR vitulina OR grey seal OR halichoerus OR horse OR horses OR equus OR equine OR equidae OR

donkey OR donkeys OR mule OR mules OR pig OR pigs OR swine OR swines OR hog OR hogs OR boar OR boars OR porcine OR piglet OR piglets OR sus OR scrofa OR llama OR llamas OR lama OR glama OR deer OR deers OR cervus OR elaphus OR cow OR cows OR bos taurus OR bos indicus OR bovine OR bull OR bulls OR cattle OR bison OR bisons OR sheep OR sheeps OR ovis aries OR ovine OR lamb OR lambs OR mouflon OR mouflons OR goat OR goats OR capra OR caprine OR chamois OR rupicapra OR leporidae OR lagomorpha OR lagomorph OR rabbit OR rabbits OR oryctolagus OR cuniculus OR laprine OR hares OR lepus OR rodentia OR rodent OR rodents OR murinae OR mouse OR mice OR mus OR musculus OR murine OR woodmouse OR apodemus OR rat OR rats OR rattus OR norvegicus OR guinea pig OR guinea pigs OR cavia OR porcellus OR hamster OR hamsters OR mesocricetus OR cricetulus OR cricetus OR gerbil OR gerbils OR jird OR jirds OR meriones OR unguiculatus OR jerboa OR jerboas OR jaculus OR chinchilla OR chinchillas OR beaver OR beavers OR castor fiber OR castor canadensis OR sciuridae OR squirrel OR squirrels OR sciurus OR chipmunk OR chipmunks OR marmot OR marmots OR marmota OR suslik OR susliks OR spermophilus OR cynomys OR cottonrat OR cottonrats OR sigmodon OR vole OR voles OR microtus OR myodes OR glareolus OR primate OR primates OR prosimian OR prosimians OR lemur OR lemurs OR lemuridae OR loris OR bush baby OR bush babies OR bushbaby OR bushbabies OR galago OR galagos OR anthropoidea OR anthropoids OR simian OR simians OR monkey OR monkeys OR marmoset OR marmosets OR callithrix OR cebuella OR tamarin OR tamarins OR saguinus OR leontopithecus OR squirrel monkey OR squirrel monkeys OR saimiri OR night monkey OR night monkeys OR owl monkey OR owl monkeys OR douroucoulis OR aotus OR spider monkey OR spider monkeys OR ateles OR baboon OR baboons OR papio OR rhesus monkey OR macaque OR macaca OR mulatta OR cynomolgus OR fascicularis OR green monkey OR green monkeys OR chlorocebus OR vervet OR vervets OR pygerythrus OR hominoidea OR ape OR apes OR hylobatidae OR gibbon OR gibbons OR siamang OR siamangs OR nomascus OR symphalangus OR hominidae OR orangutan OR orangutans OR pongo OR chimpanzee OR chimpanzees OR pan troglodytes OR bonobo OR bonobos OR pan paniscus OR gorilla OR gorillas OR troglodytes)

Web of Science Search Strategy: ((#1’’ AND #2’’) AND #3’’))

References

1. de Vries RBM, Hooijmans CR, Tillema A, Leenaars M, Ritskes-Hoitinga M. Updated version of the Embase search filter for animal studies. Lab Anim. 2014;48: 88. doi:10.1177/0023677213494374

2. Hooijmans CR, Tillema A, Leenaars M, Ritskes-Hoitinga M. Enhancing search efficiency by means of a search filter for finding all studies on animal experimentation in PubMed. Lab Anim. 2010;44: 170–175. doi:10.1258/la.2010.009117
